# An efficient method to identify, date, and describe admixture events using haplotype information

**DOI:** 10.1101/gr.275994.121

**Published:** 2022-08

**Authors:** Pongsakorn Wangkumhang, Matthew Greenfield, Garrett Hellenthal

**Affiliations:** 1 University College London Genetics Institute (UGI), Department of Genetics, Evolution and Environment, University College London, London, WC1E 6BT, United Kingdom;; 2 National Biobank of Thailand, National Science and Technology Development Agency, Pathum Thani 12120, Thailand

## Abstract

We present fastGLOBETROTTER, an efficient new haplotype-based technique to identify, date, and describe admixture events using genome-wide autosomal data. With simulations, we show how fastGLOBETROTTER reduces computation time by an order of magnitude relative to the related technique GLOBETROTTER without suffering loss of accuracy. We apply fastGLOBETROTTER to a cohort of more than 6000 Europeans from 10 countries, revealing previously unreported admixture signals. In particular, we infer multiple periods of admixture related to East Asian or Siberian-like sources, starting >2000 yr ago, in people living in countries north of the Baltic Sea. In contrast, we infer admixture related to West Asian, North African, and/or Southern European sources in populations south of the Baltic Sea, including admixture dated to ∼300–700 CE, overlapping the fall of the Roman Empire, in people from Belgium, France, and parts of Germany. Our new approach scales to analyzing hundreds to thousands of individuals from a putatively admixed population and, hence, is applicable to emerging large-scale cohorts of genetically homogeneous populations.

In recent years, numerous techniques have emerged that exploit expected patterns of linkage disequilibrium (LD) among single-nucleotide-polymorphisms (SNPs) in admixed populations that descend from the intermixing of multiple ancestral sources in order to identify, describe, and date these admixture events. Many of these techniques assume a pulse(s) of instantaneous admixture between two or more sources, followed by random mating in the admixed population (Falush et al. 2003). Under this model, the probability of inheriting two DNA segments from the same ancestral source along the genome of an admixed individual decays exponentially, with a rate proportional to the date of admixture (in generations ago) and genetic distance between the segments (Hellenthal et al. 2014). This relationship is exploited by software, including ROLLOFF (Moorjani et al. 2011; Patterson et al. 2012), ALDER (Loh et al. 2013), MALDER (Pickrell et al. 2014), GLOBETROTTER (Hellenthal et al. 2014), and MOSAIC (Salter-Townshend and Myers 2019). These approaches can date such admixture events, as well as estimate the proportions of DNA contributed by each admixing source. In contrast to other admixture inference techniques (e.g., Pool and Nielsen 2009), an additional advantage is that they do not require accurately assigning each local segment of an admixed person's genome to one of the admixing sources, which can be challenging in cases in which admixing sources are genetically similar. These techniques and others have shown admixture occurring in the past ∼4000 yr to be ubiquitous among modern human populations (Loh et al. 2013; Hellenthal et al. 2014).

To infer admixture, each technique uses a set of sampled reference populations that act as surrogates to the admixing sources. Although ROLLOFF and ALDER identify a single best surrogate for each admixing source by finding the best model fit out of pairings of available surrogates, GLOBETROTTER and MOSAIC infer the genetic make-up of each source as a mixture of DNA from all surrogate groups, that is, without requiring one prespecified surrogate per source, giving these approaches more flexibility. Furthermore, although ROLLOFF, ALDER, and MALDER do not model fine-scale haplotype information, GLOBETROTTER and MOSAIC leverage this information when inferring the probabilities of descending from each admixing source for segments along an admixed individual's genome. Using such haplotype information can be more powerful for characterizing admixture signals when using densely genotyped or sequenced individuals (Hellenthal et al. 2014; Wangkumhang and Hellenthal 2018). Also, although MALDER, MOSAIC, and GLOBETROTTER can each infer multiple dates of admixture, presently among these, only GLOBETROTTER can infer multiple pulses of admixture involving the same surrogate groups.

However, a key drawback of GLOBETROTTER is its computational complexity. In particular, inferring and dating admixture in a target population of more than 100 individuals might take GLOBETROTTER over a month on a single computing node. Here, we present fastGLOBETROTTER, a new method that increases the speed of inferring admixture events, for example, performing the same analysis in less than a day, without sacrificing accuracy relative to GLOBETROTTER. We compare both methods using simulations of admixture events with a wide range of dates, admixture proportions, and varying degrees of genetic similarity among the admixing sources. We also assess fastGLOBETROTTER's sensitivity to demographic effects like strong bottlenecks. Finally, we apply fastGLOBETROTTER to a cohort of 6209 Europeans from 10 countries genotyped at 477,417 SNPs (Sawcer et al. 2011), inferring previously unreported admixture signals spanning Europe.

## Results

### Overview of the fastGLOBETROTTER approach

GLOBETROTTER attempts to identify, date, and describe admixture in a target population of putatively admixed individuals using a set of reference populations. To do so, first the genomes of all target and reference individuals are phased using available software (e.g., Browning and Browning 2009; Delaneau et al. 2012). Next, at each SNP of the phased haploid genome *X* of a given target or reference individual, ChromoPainter (Lawson et al. 2012) infers which of a set of “donor” haploids shares ancestry most recently with *X*, with the most recently related donor typically the same over a string of contiguous SNPs. In this way, ChromoPainter describes the two phased haploids of each target individual as a mosaic of DNA segments, with each segment matching to a single donor haploid. In practice, the set of “donor” haploids is often all phased haploids from the reference populations, but they can also be only partially overlapping or entirely distinct. GLOBETROTTER then uses the ChromoPainter results for all target and reference individuals to infer and date admixture in the target population while describing the genetic make-up of each admixing source as some mixture of the reference groups.

**Figure 1.**
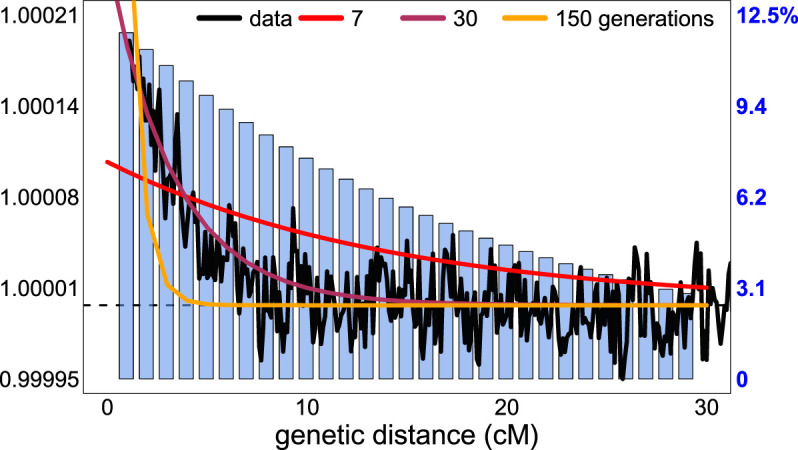
Sampling of DNA segment pairs in fastGLOBETROTTER relative to GLOBETROTTER. Scaled probability (black line), estimating Equation 4 in the Methods, that two segments separated by the given genetic distance are both inferred to share a most recent ancestor with an Irish reference individual, with a key at *left*. These probabilities are averaged across 20 simulated individuals with European (French) and South Asian (Brahui) admixture occurring 30 generations ago. Blue barplots give the proportion of segment pairs analyzed by fastGLOBETROTTER relative to those analyzed by GLOBETROTTER at each distance bin, with a key at *right*. To increase computation speed, fastGLOBETROTTER analyzes fewer segment pairs at each distance bin compared with GLOBETROTTER, but it preserves accuracy by analyzing a higher relative proportion of the more informative segment pairs separated by smaller distances. Expected scaled probabilities for three different admixture dates are shown for comparison (legend at *top*).

To do so, given the ChromoPainter-inferred donor assignment of a given DNA segment in the haploid genome of a target individual, GLOBETROTTER infers the probability that the segment is most recently related to each reference population by modeling the average proportion of genome-wide data for which each reference population shares an inferred most recent ancestor with that donor's group label (Hellenthal et al. 2014). Then, for each pair of reference populations *Y* and *Z*, GLOBETROTTER infers the probability that two DNA segments separated by genetic distance *g* have one segment most recently related to *Y* and the other segment most recently related to *Z*. After some scaling, this generates an “admixture probability curve” (also referred to as a “coancestry curve”) for *Y* and *Z* (Fig. 1, black line). GLOBETROTTER jointly analyzes the admixture probability curves for all pairwise combinations of reference populations, with the rate of change over *g* in all curves informative for the date of admixture, and the structure of the curves informative for the admixture proportions and genetic make-up of each of the admixing sources (Hellenthal et al. 2014).

By default, GLOBETROTTER considers all pairs of DNA segments separated by, for example, *g* < 50 cM when generating the admixture probability curve for *Y* and *Z*. However, for admixture events occurring more than 10 generations ago, the patterns in these curves that are attributable to admixture rapidly decline as *g* increases (Fig. 1, maroon and orange lines). Therefore, fastGLOBETROTTER uses a stochastic algorithm that preferentially selects DNA segments separated by short distances when generating admixture probability curves. Figure 1 illustrates this for a simulated example, with barplots comparing the relative proportions of segment pairs, separated by various distances, *g*’s, that fastGLOBETROTTER uses for inference relative to what GLOBETROTTER uses. Focusing on the most informative segment pairs enables fastGLOBETROTTER to ignore other less informative pairs when constructing probability curves. We show in our simulations how this increases computational speed without sacrificing accuracy.

Our new program fastGLOBETROTTER further improves upon GLOBETROTTER in a number of additional ways. First, fastGLOBETROTTER implements a technique analogous to that used in ALDER (Loh et al. 2013) to account for strong bottleneck effects in the target population potentially distorting the admixture probability curves. Second, fastGLOBETROTTER allows users to increase the memory used, by approximately the square of the number of donor populations, in order to further increase computational speed by approximately the number of chromosomes analyzed. Third, we have implemented an option to construct confidence intervals (CIs) for inferred dates by using a jackknifing approach (Busing et al. 1999), analogous to that used in ROLLOFF (Patterson et al. 2012), which thus generates CIs even when testing for admixture in single individuals. For more details of the fastGLOBETROTTER algorithm and features, see the Methods section.

### Simulations show fastGLOBETROTTER decreases computation time without sacrificing precision

**Figure 2.**
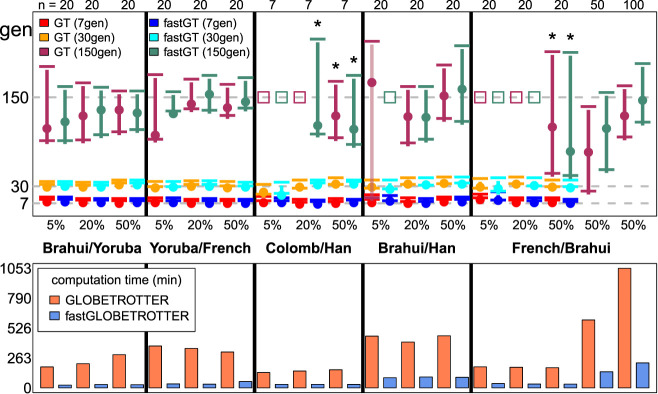
Simulations comparing inferred date accuracy and computational speed of fastGLOBETROTTER and GLOBETROTTER. (*Top*) Inferred admixture dates (+95% CI) for simulations mixing two groups (listed in *middle*), with the proportion of admixture from the second group given in the *x*-axis. For each combination of population and admixture proportion, results are given for GLOBETROTTER (GT) and fastGLOBETROTTER (fastGT) for true dates of seven, 30, and 150 generations ago (gray dashed horizontal lines). Sample size (*n*) for each simulation is given at *top*. Cases that conclude “no admixture” are depicted with an open square placed at the true date of admixture. Asterisks denote the use of a different grid for binning haplotype segments (see Methods). (*Bottom*) Computation times (in minutes, excluding bootstrapping used to generate CIs) of each approach for the scenarios above, averaging across the three different admixture dates where applicable.

We compared the performances of the GLOBETROTTER and fastGLOBETROTTER using admixed populations from Hellenthal et al. (2014) consisting of seven to 100 individuals simulated as mixtures of real or coalescent-simulated populations representing Africa, America, Central Asia, East Asia, and Europe (Fig. 2; Supplemental Fig. S1; Supplemental Tables S1, S2). As expected, typically performance in both is better when the admixing sources are more genetically different, when the admixture is more recent, and when the fraction of ancestry from the minority contributing source is higher. The accuracy and precision are similar between GLOBETROTTER and fastGLOBETROTTER in all scenarios. Inference with fastGLOBETROTTER is equally robust to GLOBETROTTER in the presence of strong bottlenecks in the target population (Supplemental Table S2). The decrease in computation time for fastGLOBETROTTER depends on the number of donors and reference populations inferred to contribute to the target. Across these simulations, fastGLOBETROTTER is about four to 12 times faster than GLOBETROTTER when inferring date point estimates and the sources and genetic make-up of each admixing population (Fig. 2), if using identical memory for each approach. However, it was about 20 times faster, with identical accuracy, if allocating more memory (in this case 1 GB) to speed up calculations (see Methods).

### Simulations illustrate limitations when inferring complicated admixture

To better understand our observed results when applying fastGLOBETROTTER to a large-scale European cohort, we also performed new simulations that mimic signals inferred in our European analysis. In these new simulations that each consider 50 admixed individuals, fastGLOBETROTTER accurately detects and describes a simple admixture event between a European source (consisting largely of people sampled from Denmark) and an East Asian (Evenk) or North African (Morocco) source occurring 100 or 200 generations ago (Table 1). However, CIs around the inferred date are wide for the 200-generation-old event involving North Africans.

**Table 1.**
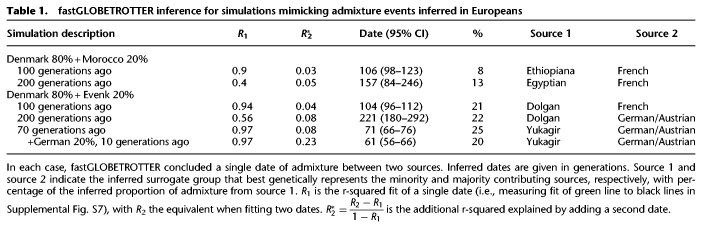
fastGLOBETROTTER inference for simulations mimicking admixture events inferred in Europeans

We also simulated an admixture event occurring 70 generations ago between European and East Asian sources, with and without an additional pulse of admixture 10 generations ago involving another European source (primarily consisting of people sampled from Germany) (Supplemental Fig. S2). However, fastGLOBETROTTER only detects a single admixture event in the case of two simulated pulses of admixture. This is not surprising, as two admixture pulses that occur relatively close in time are difficult in theory to disentangle from a single admixture event with a date between these two pulses or continuous admixture between the same sources (Hellenthal et al. 2014), with such scenarios somewhat analogous to isolation by distance models. However, the fit to the data when assuming two pulses of admixture increases by approximately threefold when we have simulated two pulses relative to only one pulse ($R_{2}^{*}$ in Table 1), which provides a potentially useful indicator of more than one admixture pulse. In addition, in the case of an additional pulse of recent admixture, the inferred date is more recent (61 generations ago; 95% CI: 56–66) relative to when there is no additional recent admixture (71 generations ago; 95% CI: 66–76). This suggests that, in the case of two pulses of admixture, the inferred event assuming one pulse lies somewhere between the initial and most recent admixture event.

### Admixture events in Europe spanning 50 BCE–1400 CE

We applied fastGLOBETROTTER to 6209 Europeans from 10 countries as previously described (Sawcer et al. 2011; Leslie et al. 2015). To account for putative structure within this cohort, we first used fineSTRUCTURE (Lawson et al. 2012) to cluster these Europeans into 86 genetically homogeneous groups, which ranged in sample size from nine to 212 individuals (Supplemental Table S3; Supplemental Fig. S3). We then applied fastGLOBETROTTER to each cluster separately, using 162 reference populations as potential surrogates for putative admixture events (Supplemental Table S4; Li et al. 2008; Behar et al. 2010; The International HapMap 3 Consortium 2010; Rasmussen et al. 2010; Chaubey et al. 2011; Henn et al. 2011; Metspalu et al. 2011; Sawcer et al. 2011; Hodoğlugil and Mahley 2012; Yunusbayev et al. 2012; Hellenthal et al. 2014; Busby et al. 2015). fastGLOBETROTTER detected admixture in 81 of the 86 clusters, inferring a simple event between two sources at a single date in 58 clusters, more than two sources admixing at around the same time in 18 clusters, multiple dates of admixture in three clusters, and an uncertain signal in two clusters (Supplemental Table S5). For populations with only one inferred date, estimated dates range from 18 to roughly 100 generations ago, which is ∼600–3000 yr ago assuming 28 yr per generation (Fenner 2005).

**Figure 3.**
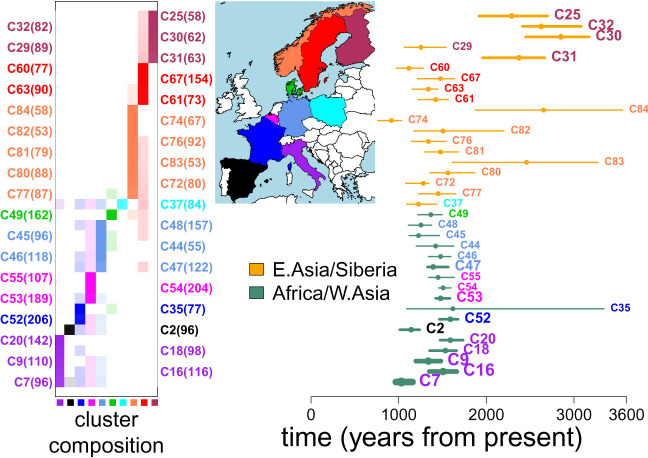
Inferred admixture events in 36 European clusters. (*Right*) Inferred admixture dates (+95% CIs) for the 36 European clusters that contain more than 50 individuals, have a single inferred date, and have a >2% inferred contribution from reference groups in East Asia/Siberia (orange CIs) or in Africa/West Asia (teal CIs). The thickness of the lines indicates the relative inferred contributions from these two regions: “Africa/W. Asia” = {West Africa, West Asia, South Middle East, North Africa, San, Central Africa, East Africa, Bantu, Ethiopian} and “E. Asia/Siberia” = {Siberia, Northeast Asia, Southeast Asia} as defined in Supplemental Table S4. (*Left*) The proportion of individuals in each cluster (row) from each country (column), with the clusters’ sample sizes in parentheses and the countries’ colors in the map. Each cluster's label at *left* and at *right* is colored according to the country most represented in that cluster.

Inferred sources of ancestry can be categorized broadly into two classes that are highly correlated with geography (Fig. 3; Supplemental Figs. S4, S5, S7–S17). The first involves ancestry related to West/Central Asia, North Africa, and/or sub-Saharan Africa, which is found to varying degrees (3%–36%), along with southern European-like ancestry, in most clusters predominantly composed of people from Belgium, Denmark, France, Germany, Spain, and Italy. The second is ancestry related to East Asians and Siberians (primarily Dolgan, Koryak, Nganasan, Oroqen, Selkup) (Supplemental Table S5), of which 2%–17% ancestry is found in nearly all clusters containing people from Finland, Norway, and Sweden, plus the cluster (C37) containing the majority of sampled Polish individuals.

Thirty-six clusters had more than 50 individuals, a single inferred admixture date, and >2% inferred ancestry from the above sources (Fig. 3). Among these 36, the highest degrees (>20%) of inferred ancestry related to West Asians and North/sub-Saharan Africans are found in three clusters that predominantly consist of Italians, with inferred dates covering a broad range spanning ∼1000–1500 yr ago (Fig. 3). Seven of these 36 clusters, consisting of Belgians, French, and Germans (clusters C44, C46, C47, C52–C55), have similar inferred amounts (5%–12%) of ancestry related to the North African and West Asian (Armenia, Morocco, Turkey) sources and similar admixture dates of ∼300–700 CE (Fig. 3; Supplemental Fig. S5).

In contrast, 18 of these 36 clusters primarily consisting of individuals from countries north of the Baltic (although with cluster C77 containing three Danish individuals) have inferred admixture events dated to ∼900–2800 yr ago involving a source related predominantly to present-day Russians and/or various groups living in Siberia. Of these 18 clusters, inferred dates are typically older in the predominantly Finnish clusters (C25, C30–C32) and are significantly more recent in nine of the 13 clusters primarily containing Swedes and Norwegians. Furthermore, 10 of these 18 clusters have some evidence of multiple pulses of admixture (i.e., $R_{2}^{*}>0.2$ in Supplemental Table S5).

## Discussion

### The fastGLOBETROTTER software

Here we introduce fastGLOBETROTTER, a new program to identify, date, and describe admixture events with four to 20 times or greater increased computational efficiency over GLOBETROTTER. Our simulation results suggest that this computational increase comes without a loss of accuracy and precision. Indeed, we see evidence of fastGLOBETROTTER outperforming GLOBETROTTER in some of the more challenging scenarios, such as when simulating admixture occurring 150 generations ago between the relatively genetically similar French and Brahui. In this scenario, fastGLOBETROTTER's point estimates are closer to the truth relative to GLOBETROTTER's when simulating 50 or 100 admixed individuals (Fig. 2). In principle, fastGLOBETROTTER's subsampling scheme, which down-weights pairs of distantly separated DNA segments when constructing the admixture probability curves, could increase accuracy over GLOBETROTTER's approach that does not down-weight such segments, particularly for older events. This is because distantly separated segments provide little to no information about the admixture event (Fig. 1) while being more susceptible to random noise owing to the smaller number of segments separated by such large distances. Although a potential concern is that this procedure may affect fastGLOBETROTTER's accuracy for dating recent admixture, for which segments separated by large distances are more informative, this does not appear to be the case in our simulations, which consider admixture as recent as seven generations ago (Fig. 2).

Caveats that apply to GLOBETROTTER apply also to fastGLOBETROTTER. For example, if inferred dates are more than 55 generations ago when using the default fastGLOBETROTTER settings that fit all DNA segment pairs separated by 30 cM, we found in simulations that better inference is achieved if rerunning to fit all DNA segment pairs separated by only 5 cM (see Methods). This is because for older events, most of the admixture signal has decayed by 5 cM, and thus, fitting segment pairs separated by larger distances can only increase noise in estimation.

As in other admixture detection methods, how well the surrogate populations reflect the true admixing sources can affect inference. In particular, using modern-day surrogates to older admixing source groups may not be ideal, because these present-day groups may have experienced drift and/or admixture not inherent in the original admixing source. Incorporating ancient DNA (aDNA) samples as surrogates could help in this regard, although it remains to be seen how much relevant aDNA can be acquired. Our simulations here (Fig. 2; Table 1; Supplemental Table S1) mimic a realistic scenario in which the true admixing source is not sampled. In these settings, fastGLOBETROTTER typically selects a close genetic match to the true admixing source as the most representative surrogate to that source. For example, the French and German/Austrian surrogates are chosen by fastGLOBETROTTER to represent the true Denmark admixing source in our European-like simulations (Table 1). These two surrogate populations have *F*_ST_ < 0.0052 with the true Denmark admixing source, which is among the lowest 10 *F*_ST_ values out of all 162 possible surrogates. These two surrogate groups have very different sample sizes, with 28 French and four German/Austrian samples, reflecting how surrogate sample size does not play a major role in inference. An exception is that sometimes surrogates with very small sample sizes (e.g., fewer than five individuals) may be favored in the model because population-specific drift may be inadequately captured by our approach (López et al. 2021).

The computational speed-ups described here are a step change over GLOBETROTTER. For example, when using its fastest option on a single computing node, fastGLOBETROTTER took just under 21 h, using ∼9 GB of RAM, to perform date/source inference and 100 bootstrap resamples using default values on European cluster C9 containing 110 individuals. In contrast, using the same input parameters, GLOBETROTTER completed the date/source inference step and only 30 bootstrap resamples after 30 d. These speed-ups enable a far more efficient analysis of larger data sets and, hence, detection of more subtle admixture signals. Nonetheless, future improvements are necessary to scale to, for example, many thousands of individuals. Furthermore, the computational gains described here apply only to the GLOBETROTTER inference step and not to the phasing and chromosome painting steps before applying GLOBETROTTER. However, we note that phasing algorithms are relatively fast, with current software able to phase tens to hundreds of thousands of individuals in a few hours on a computational cluster. ChromoPainter typically is computationally much slower than phasing but is parallelizable by both target individual and chromosome in a manner GLOBETROTTER and fastGLOBETROTTER are not, with alternative “chromosome painting” software existing that is considerably faster than ChromoPainter (e.g., Durbin 2014). Given the increasing ubiquity of large-scale genotype resources from relatively genetically homogenous populations (e.g., Chen et al. 2011; Bycroft et al. 2018), such computational speed-ups will become increasingly necessary to cope with present-day sample collections.

### Application to the European cohort

The 86 fineSTRUCTURE-inferred clusters of 6209 Europeans were largely consistent with country label (Supplemental Table S3) and likely reflect the high degree of geographic clustering previously observed in these data (Leslie et al. 2015) and using other data from these countries (Novembre et al. 2008; Humphreys et al. 2011; Kerminen et al. 2017; Van den Eynden et al. 2018; Bycroft et al. 2019; Raveane et al. 2019; Saint Pierre et al. 2020; Mattingsdal et al. 2021). However, we do not have access to any fine-scale geographic information beyond the country level, so we cannot assess whether our clusters have fine-scale geographic interpretability. Instead, we view them as convenient units meant to capture relatively genetically homogeneous groupings, although we acknowledge that cluster assignments are sometimes uncertain (Supplemental Fig. S3). Despite this, we observe broad geographic trends in inferred ancestry across these European clusters. In particular, clusters nearly exclusively containing people from countries north of the Baltic Sea (i.e., Finland, Norway, Sweden) show 2%–17% inferred ancestry from a source most closely related to East Asian and Siberian groups. In contrast, clusters containing primarily individuals from south of the Baltic (i.e., Belgium, Denmark, France, Germany, Italy, Spain), except the cluster containing the majority of Polish individuals, have <1% inferred ancestry related to East Asians and Siberians, instead showing 3%–36% inferred ancestry from sources related to Central/West Asia, North Africa, and/or sub-Saharan Africa, along with inferred ancestry related to southern Europe (Fig. 3; Supplemental Fig. S5; Supplemental Table S5).

The signals in Finland are consistent with Finns descending from an early intermixing between European and East Asian/Siberian-like sources. Plausibly related intermixing was previously reported by Saag et al. (2019), who found evidence of Siberian-like admixture appearing in aDNA samples from nearby Estonia in Late Bronze Age graves ∼2500 yr ago, in line with fastGLOBETROTTER's estimated dates. However, we note that our results do not preclude multiple episodes of intermixing involving an East Asian–like source present in the region by the Iron Age. In particular, our simulations illustrate how multiple dates of admixture between two sources, where a group genetically similar to one of the original admixing sources subsequently intermixes with the previously admixed group, may be inaccurately described by fastGLOBETROTTER as a single admixture event, sometimes with an inferred date somewhere between the dates of the two admixture events (Supplemental Fig. S2; Table 1). Supporting this, three Finnish clusters with a mean inferred date older than 200 BCE (C25, C30, C32) show some evidence of multiple pulses of admixture ($R_{2}^{*}>0.2$ in Supplemental Table S5) consistent with what we see in simulations with multiple admixture pulses (Table 1).

When moving geographically from Finland to Sweden and Norway, that is, east to west, clusters of people north of the Baltic show more recent inferred dates and decreasing proportions of inferred ancestry related to East Asia/Siberia (Fig. 3). These observations are consistent with a scenario in which a source related to northwest Europeans initially intermixed with a source related to East Asians around (or plausibly older than) 200 BCE, with this initial intermixing occurring geographically nearer to Finland than to Norway or Sweden. Subsequently, this admixed group could have intermixed with other unadmixed Europeans through migrations westward, a process that could lead to the decreased date estimates and decreased East Asian/Siberian proportions of ancestry we infer in Norway and Sweden (Fig. 3) mimicked by our simulations (Supplemental Fig. S2; Table 1). Larger sample sizes from these areas, which may allow fastGLOBETROTTER to correctly identify and date multiple pulses of admixture, and/or additional data from ancient human remains may shed light on whether this is indeed the case.

The wide range of inferred dates and ancestry proportions in Italy is consistent with multiple episodes of intermixing with sources related to present-day peoples from West/Central Asia, North Africa, and/or sub-Saharan Africa, as has been previously reported (Hellenthal et al. 2014; Busby et al. 2015; Raveane et al. 2019). In the one cluster (C2) consisting primarily of Spanish in Figure 3, the inferred date of ∼1200 yr ago involving sources with sub-Saharan African DNA matches previous reports of intermixing potentially related to the Muslim conquest of Spain (Bycroft et al. 2019).

The majority of clusters consisting of Belgians, French, and Germans (C43–C48, C51–C55) are inferred to have one date of admixture between two sources, with one genetically related to the modern-day British and Norwegians and the other genetically related to modern-day southern Europeans (Greeks, Italians), Cypriots, Moroccans, and/or Armenians (Supplemental Figs. S4, S5; Supplemental Table S5). The inferred point estimate dates of these events span a relatively small range of 400–650 CE (Supplemental Table S5), although slightly later (750–800 CE) in clusters C45 and C48. Although the historical events driving these signals are unclear, a plausible explanation is that it relates to the Roman Empire, which covered all of present-day Belgium, Germany, France, Turkey, North Africa, and elsewhere before its decline and eventual fall in 476 CE (Bengtsson 2014). In particular, individuals carrying ancestry recently related to that found in present-day people from North Africa, West Asia, and southern Europe could have moved across the empire during this time. A recent study of aDNA samples found in or near Rome spanning the time of the empire (27 BCE–300 CE) reported signals of ancestry from disparate sources genetically related to present-day people from the Near East, eastern Mediterranean, and North Africa (Antonio et al. 2019), suggesting the Empire facilitated migrants into Rome during this time. Our results, based on analyzing genetic variation data from present-day individuals, are consistent with this migration extending across the empire, with individuals carrying such ancestry intermixing with people living in or around present-day Belgium, France, and Germany either during or soon after the fall of the Roman Empire.

Our simulations suggest our inferred dates are biased to detect recent admixture (Table 1), indicating admixture we detect in these northwest Europeans may have begun before our inferred dates of ∼400–650 CE. However, a previous study reported genetic patterns in aDNA from Bavarians dated to ∼500 CE showed no clear genetic affinities to present-day southern Europeans, consistent with a lack of widespread intermixing between local and southern sources in Germany before our inferred admixture dates (Veeramah et al. 2018). This was despite that study reporting the presence of a presumed Roman soldier in Munich dated to ∼300 CE, which had strong genetic affinities to present-day southern Europeans (Veeramah et al. 2018). Additional aDNA samples from these northwest European regions may help clarify these signals.

Other German clusters (C38, C39, C41, C42) do not appear to have this South Europe/West Asian/African signal, instead showing inferred signals of ancestry sources related to eastern European groups such as Poland (Supplemental Table S5) and significantly more recent dates around 1100–1400 CE, perhaps reflecting geography.

Overall, these findings further illustrate the ability of genetic data to shed light on intermixing among genetically different groups that may relate to well-attested historical events.

## Methods

### Brief overview of GLOBETROTTER/fastGLOBETROTTER methodology

The theory behind fastGLOBETROTTER is analogous to that in GLOBETROTTER, which has been previously described (Hellenthal et al. 2014). In brief, consider an admixed population that descends from the mixture of two source groups, *A* and *B*, that contributed α and 1 − α of the DNA, respectively, and intermixed λ generations ago. Assuming random mating among admixed individuals since the time of admixture and the crossovers between any two loci occur at random (i.e., no crossover interference) (Falush et al. 2003), the probability *P*_*A*→*B*_(*g*|λ, α) that two loci separated by genetic distance *g* (in Morgans) along a chromosome within a haploid genome of an admixed individual, with one locus descending from an individual from *A* and the other from an individual in *B*, is

(1) $$P_{A\to B}(g|\lambda ,\alpha )=\alpha (1-\alpha )-\alpha (1-\alpha )\exp^{-g\lambda }.$$

The probability *P*_*A*→*A*_(*g*|λ, α) that the two loci both descend from individuals from *A* is

(2) $$P_{A\to A}(g|\lambda ,\alpha )=\alpha^{2}+\alpha (1-\alpha )\exp^{-g\lambda }.$$

(Analogous formulas can be derived for more than two admixing sources [Hellenthal et al. 2014].) Note that α^2^ is the marginal probability that two independent loci (e.g., separated by a large distance *g*) both derive from source *A*, with *α*(1 − *α*) similarly the marginal probability that two independent segments derive from *A* and *B*. Dividing Equations 1 and 2 by *α*(1 − *α*) and *α*^2^, respectively, gives

(3) $$\frac{P_{A\to B}(g|\lambda ,\alpha )}{\alpha (1-\alpha )}=1-\exp^{-g\lambda }\equiv 1+\delta_{AB}\exp^{-g\lambda },$$

and

(4) $$\frac{P_{A\to A}(g|\lambda ,\alpha )}{\alpha^{2}}=1+\frac{\left(1-\alpha \right)}{\alpha }\exp^{-g\lambda }\equiv 1+\delta_{AA}\exp^{-g\lambda }.$$

Importantly, δ_*AB*_ < 0 and δ_*AA*_ > 0, ensuring that Equation 3 is monotonically increasing with *g*, whereas Equation 4 is monotonically decreasing with *g*. As the true admixing sources *A* and *B* are unknown, we instead consider the probability that two segments separated by distance *g* are inferred to share most recent ancestry with reference populations *U* and *V*. If *U* and *V* are good surrogates for the same source (e.g., source *A*), then this scaled probability curve will be decreasing. In contrast, if *U* is a good surrogate for source *A* and *V* a good surrogate for *B*, this curve will be increasing. GLOBETROTTER and fastGLOBETROTTER exploit these relationships to infer *α* and the genetic make-up of each source group as mixtures of the reference populations, with the shape of the scaled probability curves used to infer λ, as described by Hellenthal et al. (2014). Briefly, for each {*U*, *V*}, we construct ${\hat{P}}_{U\to V}(g|\lambda ,\alpha )$, which we refer to as “admixture probability curves,” that are meant to reflect the left-hand side of Equations 3 and 4. We then find the values of λ, τ_*u*,*v*_, and δ_*u*,*v*_ for all *u*, *v* that minimize the following sum of squared errors:

(5) $$\underset{u,v}{\sum }\underset{g}{\sum }{\left({\hat{P}}_{U\to V}(g|\lambda ,\alpha )-\tau_{u,v}-\delta_{u,v}\exp^{-g\lambda }\right)}^{2}.$$

Note that τ_*u*,*v*_ is estimated here, rather than set to one as in the theoretical results 3 and 4, although it often is inferred to be close to one in practice. An example of ${\hat{P}}_{U\to V}(g|\lambda ,\alpha )$ that reflects Equation 4 is given for simulations in Figure 1, with examples of ${\hat{P}}_{U\to V}(g|\lambda ,\alpha )$ reflecting Equation 3 for real data given in Supplemental Figures S7 through S17.

### Details of DNA segment pair selection algorithm in fastGLOBETROTTER

These admixture probability curves are constructed as follows. We first use ChromoPainter (Lawson et al. 2012) to compose each phased haploid of individuals from a putatively admixed population as a sequence of nonoverlapping DNA segments, where each segment is inferred to share most recent ancestry with a donor individual (e.g., an individual from one of the reference populations). Technically, a DNA segment is defined as a contiguous set of SNPs in the target haploid that is inferred by ChromoPainter to share the most recent ancestor with the same donor haploid. In practice, ChromoPainter generates *s* (typically *s* = 10) inferred genome-wide sequences of these segments for each target haploid, giving 2*s* inferred sequences across an individuals’ two haploid genomes. Assume that these 2*s* sequences contain *C* DNA segments in total for the individual.

To construct admixture probability curves that reflect Equations 3 and 4, GLOBETROTTER uses all $\left(\begin{array}{c} C\\ 2 \end{array}\right)$ pairings of DNA segments within an individual that are (1) on the same chromosome and (2) separated by ≤*K* centimorgans (e.g., *K* = 30). This is among the most computationally intensive steps of GLOBETROTTER, with complexity squared in the maximum number of DNA segments matched to the same donor population across chromosomes. In contrast, fastGLOBETROTTER only uses a subset of all possible pairings of the *C* DNA segments.

The subsampling algorithm of fastGLOBETROTTER is as follows:

1. Divide the genome into *B* nonoverlapping bins of size *X* cM. *X* is the *bin.width* specified by the user; here, we use *X* = 0.1 unless otherwise noted.
2. Find which of the *C* total segments fall into bin *G*_*i*_ for all *i* ∈ [1, …, *B*]. A segment will be put into bin *G*_*i*_ if the midpoint of the segment falls within the range of bin *G*_*i*_. Let *N*_*i*_ be the number of segments within bin *G*_*i*_, where $\sum_{i=1}^{B}N_{i}=C$.
3. For each bin *G*_*i*_, the program will compare the segments in this bin to those in bin *G*_*i*+1_, where the distance *D*_*i*→*i*+1_ between *G*_*i*_ and *G*_*i*+1_ is *X* cM. The program then compares *G*_*i*_ with *G*_*i*+2_ (i.e., with distance *D*_*i*→*i*+2_ = 2*X* between them) and so on, until reaching the last bin *n* with *D*_*i*→*n*_ ≤ *K*, where *K* is the maximum allowed distance between segments. Here we use *K* = 30 cM, noting that segments on different chromosomes are never compared. Also note that segments within the same bin *G*_*i*_, which by definition have distance between them <*X* cM, will not be compared to each other. However, this seems desirable as segments at such short distances can be confounded by background LD unrelated to the admixture signal. The user can also specify to avoid fitting segments separated by less than some distance; here, we use the default value of not fitting segments separated by ≤1 cM.
4. To do the comparison in step 3, we do the following:
  - For each *i* and *j*, where *i* < *j*, calculate *Y*_*ij*_ = *N*_*i*_ × *N*_*j*_ × *M*_*ij*_, which is the number of samplings of segment pairs from bins *G*_*i*_ and *G*_*j*_ to be performed, that is, with one segment sampled from bin *G*_*i*_ and the other sampled from bin *G*_*j*_. *M*_*ij*_ is a scalar that is derived from a distribution that allows us to sample a different proportion of the total segment pairs in bins *G*_*i*_ and *G*_*j*_. For example, if *M*_*ij*_ = 1, a roughly equivalent number of segment pairs will be sampled as in the original GLOBETROTTER. Alternatively, one could make *M*_*ij*_ < 1 and set a higher value of *M*_*ij*_ for segment pairs with smaller *D*_*i*→*j*_, meaning closer pairs are preferentially sampled over more distant pairs. Here we use $M_{ij}=\exp^{-\gamma D_{i\to j}}/c$, with γ = 0.05 and *c* = 8, which performs well in simulations in terms of computation decrease while maintaining precision (Fig. 2). With these values, the number of segment pairs sampled is ∼6.5% of the total possible pairs separated by ≤30 cM.
  - To compare segments in *G*_*i*_ and *G*_*j*_, the program randomly samples *Y*_*ij*_ segment pairs without replacement, with one segment from *G*_*i*_ and the other from *G*_*j*_.
5. Repeat step 4 for all pairs of bins (*G*_*i*_, *G*_*j*_) across the chromosome separated by ≤*K* cM.

In addition, Hellenthal et al. (2014) described a “null individual” analysis that aims to eliminate LD decay signals in the admixture probability curves that are not attributable to admixture, hence providing more reliable date estimates. This is performed by building a “null” probability curve using segment pairs in which each segment is from the painting sample of a different target individual (using at most 100 individuals from the target population, for computational efficiency). This “null” probability curve should be unrelated to the admixture event because segment pairs on different individuals cannot fall within a single block of DNA inherited intact from an admixing source individual. GLOBETROTTER scales each admixture probability curve by this “null” probability curve before inferring dates and proportions of admixture, which can lead to more accurate inference, particularly in cases in which the target population has experienced a strong bottleneck (e.g., see Supplemental Table S2; Hellenthal et al. 2014). To implement this “null” individual protocol into fastGLOBETROTTER, we replace the following two steps in the above algorithm:

- Step 2. For *T* admixed target individuals, let *P*_*null*_ be a vector of size $\sum_{\;j=1}^{T}C_{j}$, with *C*_*j*_ as the total number of segments for target individual *j*, and where *P*_*null*_(*c*) stores the index of the admixed target individual to which segment *c* belongs.
- Step 4B. To compare segments in *G*_*i*_ and *G*_*j*_, the program randomly samples segment pairs, with one from *G*_*i*_ (call this segment *a*_*i*_) and the other from *G*_*j*_ (call this segment *b*_*j*_). When building the “null” probability curve, we only consider segment pairs where *P*_*null*_(*a*_*i*_) ≠ *P*_*null*_(*b*_*j*_), with segments randomly chosen until *Y*_*ij*_ pairs meet this criterion.

After constructing the “admixture probability curves” as described above, the inference steps in fastGLOBETROTTER are the same as those previously described (Hellenthal et al. 2014) for GLOBETROTTER, except the few changes detailed below. As in GLOBETROTTER, we use bootstrap resampling of individuals’ chromosomes to construct CIs around inferred dates. Also, as previously described (Hellenthal et al. 2014), if any of these bootstrap-inferred dates contains one or is ≥400, we conclude no evidence of admixture, because such inference is consistent with no signal in the admixture probability curves.

As a comparison, the computational time of the original GLOBETROTTER algorithm can be described as

$$O[TZ(R+Q)(sL+H^{2}I+H^{2}I_{h}^{2}+BH^{2}F^{2})+C{\left[\min(T,100)\right]}^{2}(L+I_{h}^{2})],$$

where *T* is the number of the target population individuals, *Z* is the number of chromosomes, *R* is the number of bootstrap resamples, *Q* is the number iterations of inferring dates and inferring source groups and admixture contributions, *s* is the number of painting samples, *L* is the maximum number of SNPs across chromosomes, *H* is the number of donor populations, *I* is the maximum number of chunks across chromosomes and individuals, *I*_*h*_ is the maximum number of chunks across chromosomes and individuals that are copied from a single donor population *h*, *B* is the number of bins, and *F* is the number of surrogates.

In contrast, the computational time of fastGLOBETROTTER is

$$O[(R+Q)(TZ(sL+H^{2}I+I_{h}^{2})+TBH^{2}F^{2})+C{\left[\min T,100\right]}^{2}(L+I_{h}^{2})].$$

### Details of approach to adjust for extensive LD within the target population in fastGLOBETROTTER

In admixed groups affected by strong bottlenecks, DNA segments matched to donors using ChromoPainter may be atypically long, reflecting high levels of within-population LD. To cope with this, GLOBETROTTER ignores any chunk pairs separated by ≤1 cM when generating admixture probability curves, implicitly assuming such within-population LD is unlikely to extend beyond 1 cM. However, this threshold is arbitrary; other methods like ALDER (Loh et al. 2013) attempt to automatically identify the threshold of minimal distance between segments to use when capturing admixture LD. A particular concern is that the presence of many atypically long chunks >1 cM can mimic patterns in the admixture probability curves that are similar to those expected under multiple distinct pulses of admixture involving different groups admixing at different times (see Hellenthal et al. 2014), hence leading to inaccurate admixture inference.

To cope with this issue, before model fitting, fastGLOBETROTTER automatically removes the left-end portions of the admixture probability curves that are believed to be affected by within-population LD. To do so, we first analyze the admixture probability curve of the surrogate group inferred to contribute the highest proportion of ancestry, which is likely to be the most informative and clear curve. This curve provides the scaled probability that two segments separated by distance *g* both match to this surrogate group, with *g* binned to the nearest (e.g.,) 0.1 cM. In the absence of within-population LD, this curve should be monotonically decreasing. Therefore, starting at the left-most distance grid-point *x*, we fit a simple linear regression of the scaled probability versus distance for a window of *W* adjacent distance bins, that is, fitting a linear regression from distance grid-points *x* to *x* + *W*. The value of *x* is user-supplied; in all analyses here, we use the grid-point corresponding to 1 cM, meaning that two segments separated by <1 cM are ignored. If the fitted slope of this regression is greater or equal to zero, we shift the window one distance bin to the right, that is, now fitting a linear regression from distance grid-points *x* + 1 to *x* + *W* + 1. We repeat this process until the fitted slope is less than zero. Letting *x*_*l*_ be the left-most distance grid-point in this *W*-length window where the regression slope was less than zero for the first time, we record *x*_*w*_ = *x*_*l*_ + ceiling(*W*/2) as the right endpoint of the region to remove. We repeat this protocol for windows of size *W* = {3, 5, 7, 9, 11, 13}, and use the maximum value of *x*_*w*_ across all *W* as the left-end portion we eliminate from all admixture probability curves before inferring admixture. In its current implementation, at most half of the total fitted distance specified by the user can be removed. We note that considering only the admixture probability curve of the maximally contributing surrogate group may not be sufficient to adjust for within-population LD effects in all admixture probability curves, although this approach worked well in practice. In this paper, we used this default LD-removal step for the analysis of all European populations and the simulated European populations.

### Details of memory and speed trade-offs in fastGLOBETROTTER

One of the steps of GLOBETROTTER, unaffected by the speed-ups mentioned above, has computational cost that is squared in the number of donor groups included in the ChromoPainter analysis. This calculation is performed once per individual per chromosome in GLOBETROTTER. In contrast, fastGLOBETROTTER gives the option of performing this calculation once per individual for all chromosomes, which in practice we found to be about two times faster than the standard fastGLOBETROTTER while giving the same results. However, this approach incurs a memory increase equal to the square in the number of donor groups divided by the square of the number of surrogate groups that contribute more than a user-specified minimum amount of ancestry. Here we only include surrogate groups that contribute >0.5% of ancestry. We have implemented an option in fastGLOBETROTTER that does a quick estimate of the amount of memory increase necessary to perform this new step, enabling users to decide whether the speed-up is worth the memory increase.

Because of the above, reducing the number of donor groups used by fastGLOBETROTTER can alleviate both the computational and memory constraints. Here we have also implemented an option to combine donor groups that share a similar genetic background. To do so, we first find the average proportion of genome-wide DNA that individuals from each surrogate group match to haploids from each donor group, standardizing this to sum to 1.0 in each surrogate. Thus, we describe each surrogate by a vector of length equal to the number of donor groups. Conversely, each donor group can be defined by a vector containing the amount they contribute to each surrogate group. For all pairs of donor groups, we find the Pearson's correlation of these donor vectors. For donor vectors with a correlation >0.95, we merged their values within each surrogate group by averaging those donors’ contributions to that surrogate group. However, for the applications considered here, we found little additional reduction in computation time or memory when using this approach relative to that described in the previous paragraph, cautioning that it may not be worth the potential loss in power from joining donor groups.

### Details of jackknifing procedure to infer CIs for dates in fastGLOBETROTTER

The algorithm GLOBETROTTER uses bootstrap resampling of individuals’ chromosomes to construct CIs for inferred admixture dates. However, bootstrap resampling is not possible when inferring admixture in a single individual. Therefore, following a method previously described (Patterson et al. 2012), fastGLOBETROTTER also includes an alternative jackknifing procedure that instead drops one chromosome at a time and estimates the dates using data from the other 21 chromosomes. This process provides 22 estimated date values, which can then be used to give CIs for the inferred admixture date using previously derived weighted jackknifing formulas (e.g., Busing et al. 1999). Following the method of Busing et al. (1999), for *Z* = 22 chromosomes, here we calculate the jackknife standard error (σ_JK_) as

$$\sigma_{\mathrm{JK}}={\left(\frac{1}{Z}\overset{Z}{\underset{i=1}{\sum }}\frac{1}{h_{i}-1}{\left[h_{i}\lambda -(h_{i}-1)\lambda_{i}-Z\lambda +\overset{Z}{\underset{\;j=1}{\sum }}\left(1-\frac{w_{j}}{Z}\right)\lambda_{j}\right]}^{2}\right)}^{\left(1/2\right)},$$

where λ is the inferred date using all SNPs, λ_*i*_ is the inferred date after removing chromosome *i*, *w*_*i*_ is the number of SNPs in chromosome *i*, and $h_{i}=\left[\sum_{\;j=1}^{Z}w_{j}\right]/w_{i}$.

We provide a comparison of standard deviations calculated from bootstrapping versus jackknifing in Supplemental Figure S6 for the European clusters. The values are notably correlated, although jackknifing gives larger values overall as expected (Efron and Gong 1983), suggesting this should only be used when testing for admixture in populations with small sample sizes. In particular, this will be useful in cases in which there is only a single target sample, in which case bootstrapping is not possible.

### Simulations from Hellenthal et al. (2014)

To compare fastGLOBETROTTER to GLOBETROTTER, we used two sets of simulated data sets described by Hellenthal et al. (2014). In the first case, each simulated haploid genome of the admixed individuals was generated as a mosaic of DNA segments from the phased genomes of individuals from the two admixing source populations, using the technique described by Price et al. (2009) and real sampled individuals for the source populations. The following combinations of populations were used as the two admixing sources: Yoruba (Africa) versus French (Europe), French versus Brahui (West Asia), Brahui versus Han (East Asia), Brahui versus Yoruba, and Colombian (America) versus Han. For each of these population pairs, seven (Colombian vs Han) or 20 (all others) individuals were simulated for each combination of admixture date at seven, 30, and 150 generations ago and fraction of admixture from the second source of 0.05, 0.2, and 0.5 (Fig. 2; Supplemental Table S1). In particular, the size (in Morgans) of each DNA segment for an admixed haploid was sampled randomly from an exponential distribution with rate equal to the date of admixture, and this segment was copied intact from a source haploid chosen randomly from an admixing source population with probability equal to the desired admixture fraction from that source population. Here, we also created new simulations of 100 admixed individuals in this manner for the case of admixture between French and Brahui at 150 generations ago and an admixture fraction of 0.5. In each case, we inferred admixture using the 91 other sampled populations from Hellenthal et al. (2014) as reference populations and using the ChromoPainter procedure described in that paper.

The other set of simulations from Hellenthal et al. (2014) first used the program MaCS (Chen et al. 2009) to simulate 11 populations meant to mimic the demographic history of Africa (Pops 1–4), West Eurasia (Pops 5–7), and East Asia (Pops 8–11) (see Supplemental Fig. S1). Next, an admixed population was generated by randomly sampling 100 haploid genomes from a population composed of 150 and 100 simulated haploid genomes from populations 2 and 8, respectively. Thus, this admixed population mimics a scenario with 60% and 40% ancestry inherited from Africa and East Asia, respectively, with its small population size (50 individuals) mimicking the effects of a strong bottleneck. This admixed population was then simulated forward-in-time by randomly selecting parents in the current generation to construct each offspring's two haploid genomes in the next generation, mixing the parental genomes according to recombination probabilities from the HapMap Phase 2 genetic map and generating 50 offspring in total. Separate simulations ran this forward-in-time procedure for 10, 20, and 45 nonoverlapping generations, mimicking different dates of admixture. To infer the admixture, we used populations 2, 4, 9–11, and six other admixed populations (PopA–PopF in Hellenthal et al. 2014) as reference populations using the procedure described in that paper.

### Simulations mimicking European admixture results

Using the approach of Price et al. (2009), we also generated new simulated populations to mimic scenarios that might explain patterns observed in our new analysis of European populations. We used four populations as admixing sources.

- “Danish”: 200 individuals generated using European cluster C49, where C49 contains 162 Europeans, primarily Danish (Supplemental Table S3; Sawcer et al. 2011).
- “German”: the 134 individuals, primarily German, from European cluster C38 (Sawcer et al. 2011).
- “Moroccans”: 25 individuals from Morocco (Behar et al. 2010; Hellenthal et al. 2014).
- “Evenk”: 12 Evenks from northern Asia (Rasmussen et al. 2010).

The “Danish” source consisted of a population of 200 individuals generated by intermixing C49 individuals using the approach of Price et al. (2009), with segment sizes (in Morgans) determined by an exponential distribution with rate equal to 200. This was performed to mitigate the signal of genuine admixture in C49 (e.g., see Fig. 3) from the simulated individuals.

We simulated four different scenarios, each consisting of 50 admixed individuals.

1. “EuroSim1”: 80%/20% of ancestry from Danish/Moroccans, intermixing 100 or 200 generations ago.
2. “EuroSim2”: 80%/20% of ancestry from Danish/Evenk, intermixing 100 or 200 generations ago.
3. “EuroSim3”: admixture 70 generations ago between Danish/Evenk at 80%/20%, with no subsequent admixture.
4. “EuroSim4”: admixture 70 generations ago between Danish/Evenk at 80%/20%, followed by this admixed population intermixing with Germans that contribute 20% of ancestry 10 generations ago (as in Supplemental Fig. S2).

The first of these new European-based simulations is designed to mimic the intermixing of groups related to North Africa (represented by Morocco) and Europe that we observe for several European clusters consisting of Belgians, French, and Germans. The remaining three simulations assess our model's ability to characterize gene flow between Siberian-related groups and Scandinavian populations, which we observe for clusters containing Finns, Norwegians, and Swedes, at one or multiple dates. We applied ChromoPainter and fastGLOBETROTTER to each simulation using the same protocol as our real data analysis, although excluding as references for EuroSim1 the Moroccan population used to simulate and excluding as references for EuroSim2–EuroSim4 the Evenk population used to simulate. This reflects a realistic scenario in which none of the admixing populations were sampled. For computational simplicity, we used the same paintings of reference populations against each other as was used in the real data analysis, by setting the amount that each other reference population matched to Evenk or Morocco (using either as appropriate) to zero and rescaling. This may result in a slight loss in accuracy in these simulations. Results are given in Table 1.

### Analysis of simulations

For the simulations from Hellenthal et al. (2014), we applied GLOBETROTTER and fastGLOBETROTTER to ChromoPainter output generated as previously described (Hellenthal et al. 2014). When analyzing each simulation with GLOBETROTTER and fastGLOBETROTTER, we used the default value of five iterations of inferring dates versus inferring admixture proportions and sources, at each iteration removing reference populations inferred to contribute <0.5% of ancestry. In most cases, we constructed admixture probability curves by only considering pairs of DNA segments 1–30 cM apart, rounding the distances between pairs to the nearest 0.1 cM. An exception is our analysis of the Colombian–Han and French–Brahui simulations with admixture 150 generations ago and 20 or fewer admixed individuals, where we instead only considered DNA segments 1–5 cM apart rounded to the nearest 0.05 cM, as recommended by Hellenthal et al. (2014) when inferred admixture dates are more than 55 generations ago when using the default values. In all cases, we used 100 bootstrap resamples of simulated target population individuals to construct CIs for inferred dates. Following the method of Hellenthal et al. (2014), a simulation was considered to have no admixture if the floor of the minimum bootstrap date was one or if the maximum bootstrap date was 400 or greater, as the former case is consistent with no admixture and the latter is older than what we can reliably infer with these sample sizes.

### Application to the European cohort

We explored admixture in 6209 European individuals sampled from Belgium, Denmark, Finland, France, Germany, Italy, Norway, Poland, Spain, and Sweden genotyped on the Illumina Human 660-Quad SNP array (Sawcer et al. 2011). These individuals were multiple sclerosis cases. As reference populations, we used 4309 individuals sampled from 162 worldwide populations genotyped on a similar platform (Supplemental Table S4; Li et al. 2008; Behar et al. 2010; The International HapMap 3 Consortium 2010; Rasmussen et al. 2010; Chaubey et al. 2011; Henn et al. 2011; Metspalu et al. 2011; Sawcer et al. 2011; Hodoğlugil and Mahley 2012; Yunusbayev et al. 2012; Hellenthal et al. 2014; Busby et al. 2015). Different data sets were merged using PLINK (Purcell et al. 2007), after which SNPs with minor-allele frequency <1% or missingness >10% were removed, resulting in 477,417 autosomal SNPs for analysis.

As the precise origins of the 6209 Europeans were unknown beyond the country level, we clustered them into genetically homogeneous groups before inferring admixture. To do so, we first phased individuals jointly using SHAPEIT (Delaneau et al. 2013) with the build 36 genetic map. Next, we ran ChromoPainter to form (“paint”) the two phased haploids from each of the 10,522 total individuals as a mosaic of those from all other 10,521 individuals. In particular, we estimated the genome-wide average switch (-n flag) and global emission rate (-M flag) by applying 10 iterations of the ChromoPainter expectation–maximization algorithm to paint the phased haploids of 1052 individuals across chromosomes {4, 10, 15, 22}, painting only one-tenth of individuals and four chromosomes for computational simplicity. We then averaged the estimated values of switch and emission rates across these chromosomes and individuals, giving 52.82727348 and 0.000134461, respectively, and ran ChromoPainter on each of the 10,522 individuals using these fixed values. We applied fineSTRUCTURE (Lawson et al. 2012) to this ChromoPainter output, clustering the 6209 Europeans and people from the Abhkasian, Greek, and Maya reference populations using 5 million Markov chain Monte Carlo (MCMC) iterations while fixing the other 159 reference populations as “superpopulations” (-f switch). FineSTRUCTURE sampled one MCMC iteration out of every 10,000, selected the sample from among these with the highest posterior probability, and used 10,000 additional optimization steps under a greedy approach to find a clustering solution with higher posterior probability (Lawson et al. 2012). This procedure assigned the Europeans and reference populations into 319 clusters, which fineSTRUCTURE then merged hierarchically, two at a time, under a greedy approach until only two clusters remained. We moved up this fineSTRUCTURE tree and stopped at a level of the tree where no cluster containing the 6209 target Europeans had fewer than nine individuals. This gave 86 clusters with European individuals that we analyzed in subsequent analyses. Cluster names, sample sizes, and population descriptions are provided in Supplemental Table S3.

To detect admixture events, we applied fastGLOBETROTTER separately to each of these 86 European clusters, using the 162 reference populations as surrogates to the putative admixing sources. To do so, we used ChromoPainter to form the phased haploids of individuals in all 86 clusters and 162 surrogate (reference) populations as mosaics of those from the 162 reference populations. When doing so, we used the same fixed switch and emission parameters in the fineSTRUCTURE analysis for computational convenience. For each of the 86 + 162 populations, we tabulated the average proportion of genome-wide DNA that individuals from that population match to any haploid in each of the 162 reference populations. Note that an individual is not allowed to match to themselves, so each person in a surrogate population matches to one fewer member from their own population than is the case with individuals in all other surrogate populations and the 86 European clusters. This asymmetry may influence inference of the sources involved in the admixture event, although previous analyses have not found this to have a noticeable effect when using similar sample sizes (Hellenthal et al. 2014; López et al. 2021). An exception is surrogate populations with small sample sizes (e.g., two or fewer individuals), which may be upweighted as an ancestry contributor relative to surrogate populations with larger sample sizes (López et al. 2021).

For each haploid from the 86 European clusters, we also used ChromoPainter to generate 10 stochastic samples of their mosaic matching to haploids from the 162 reference populations. As in GLOBETROTTER, fastGLOBETROTTER takes as input both the mosaic painting samples of all target population individuals and the average proportion of DNA that the target and 162 surrogate populations matches to each of the 162 reference populations, using these to infer dates and infer admixture sources/proportions (Hellenthal et al. 2014), respectively, in an iterative manner. We used five iterations of alternating between inferring dates and inferring admixture proportions and sources, while removing reference populations inferred to contribute <0.5% of ancestry at each iteration. We also used the “null individual” analysis (i.e., null.ind = 1) that adjusts date inference for the effects of a postadmixture bottleneck in the target population. Following our simulations, we constructed admixture probability curves by only considering pairs of DNA segments 1–30 cM apart, rounding the distances between pairs to the nearest 0.1 cM. In cases in which this gave an inferred admixture date more than 55 generations ago, we reinferred admixture dates and proportions, using five iterations as above, while only considering DNA segments 1–5 cM apart rounded to the nearest 0.05 cM, as recommended by Hellenthal et al. (2014). We used 100 bootstrap resamples of individuals’ chromosomes to construct 95% CIs for the inferred admixture date(s). Dates are inferred in generations (*g*), which were converted to years (*y*) using the formula *y* = 1960 − 28 × (*g* + 1), which assumes a generation time of 28 yr (Fenner 2005) and an average birthdate of 1960 for sampled individuals. One cluster, C23, inferred multiple admixture dates but with a very large inferred older date (more than 700 generations ago) beyond that which we expect to be able to reliably infer admixture; we therefore excluded this cluster from our results.

### Data sets

All data used in this paper are previously published, from https://ega-archive.org/datasets/EGAD00000000120 (Sawcer et al. 2011), https://hagsc.org/hgdp/files.html (Li et al. 2008), https://www.ncbi.nlm.nih.gov/geo/query/acc.cgi?acc=GSE37342 (Yunusbayev et al. 2012), https://www.ncbi.nlm.nih.gov/geo/query/acc.cgi?acc=GSE22494 (Rasmussen et al. 2010), https://www.ncbi.nlm.nih.gov/geo/query/acc.cgi?acc=GSE21478 (Behar et al. 2010), https://www.sanger.ac.uk/resources/downloads/human/hapmap3.html (The International HapMap 3 Consortium 2010), https://www.omicsdi.org/dataset/arrayexpress-repository/E-GEOD-33489 (Metspalu et al. 2011), https://www.pnas.org/doi/full/10.1073/pnas.1017511108 (Henn et al. 2011), https://academic.oup.com/mbe/article/28/2/1013/1220271 (Chaubey et al. 2011), https://onlinelibrary.wiley.com/doi/10.1111/j.1469-1809.2011.00701.x (Hodoğlugil and Mahley 2012), and https://data.mendeley.com/datasets/ckz9mtgrjj/3 (Hellenthal et al. 2014).

### Software availability

The software fastGLOBETROTTER is available at GitHub (https:// github.com/hellenthal-group-UCL/fastGLOBETROTTER), including a detailed tutorial with example files, and as Supplemental Code.

## Supplementary Material

Supplemental Material

## References

[GR275994WANC1] Antonio ML, Gao Z, Moots HM, Lucci M, Candilio F, Sawyer S, Oberreiter V, Calderon D, Devitofranceschi K, Aikens RC, 2019. Ancient Rome: a genetic crossroads of Europe and the Mediterranean. Science 366: 708–714. 10.1126/science.aay6826 31699931 PMC7093155

[GR275994WANC2] Behar DM, Yunusbayev B, Metspalu M, Metspalu E, Rosset S, Parik J, Rootsi S, Chaubey G, Kutuev I, Yudkovsky G, 2010. The genome-wide structure of the Jewish people. Nature 466: 238–242. 10.1038/nature09103 20531471

[GR275994WANC3] Bengtsson BO. 2014. Strange history: the fall of Rome explained in *Hereditas*. Hereditas 151: 132–139. 10.1111/hrd2.00080 25588300

[GR275994WANC4] Browning BL, Browning SR. 2009. A unified approach to genotype imputation and haplotype-phase inference for large data sets of trios and unrelated individuals. Am J Hum Genet 84: 210–223. 10.1016/j.ajhg.2009.01.005 19200528 PMC2668004

[GR275994WANC5] Busby G, Hellenthal G, Montinaro F, Tofanelli S, Bulayeva K, Rudan I, Zemunik T, Hayward C, Toncheva D, Karachanak-Yankova S, 2015. The role of recent admixture in forming the contemporary West Eurasian genomic landscape. Curr Biol 25: 2518–2526. 10.1016/j.cub.2015.08.007 26387712 PMC4714572

[GR275994WANC6] Busing F, Meijer E, Van Der Leeden R. 1999. Delete-m jackknife for unequal m. Stat Comput 9: 3–8. 10.1023/A:1008800423698

[GR275994WANC7] Bycroft C, Freeman C, Petkova D, Band G, Elliott L, Sharp K, Motyer A, Vukcevic D, Delaneau O, O'Connell J, 2018. The UK Biobank resource with deep phenotyping and genomic data. Nature 562: 203–209. 10.1038/s41586-018-0579-z 30305743 PMC6786975

[GR275994WANC8] Bycroft C, Fernandez-Rozadilla C, Ruiz-Ponte C, Quintela I, Carracedo A, Donnelly P, Myers S. 2019. Patterns of genetic differentiation and the footprints of historical migrations in the Iberian Peninsula. Nat Commun 10: 551. 10.1038/s41467-018-08272-w 30710075 PMC6358624

[GR275994WANC9] Chaubey G, Metspalu M, Choi Y, Mägi R, Romero IG, Soares P, van Oven M, Behar DM, Rootsi S, Hudjashov G, 2011. Population genetic structure in Indian Austroasiatic speakers: the role of landscape barriers and sex-specific admixture. Mol Biol and Evol 28: 1013–1024. 10.1093/molbev/msq288 20978040 PMC3355372

[GR275994WANC10] Chen GK, Marjoram P, Wall JD. 2009. Fast and flexible simulation of DNA sequence data. Genome Res 19: 136–142. 10.1101/gr.083634.108 19029539 PMC2612967

[GR275994WANC11] Chen Z, Chen J, Collins R, Guo Y, Peto R, Wu F, Li L, China Kadoorie Biobank (CKB) collaborative group. 2011. China Kadoorie Biobank of 0.5 million people: survey methods, baseline characteristics and long-term follow-up. Int J Epidemiol 40: 1652–1666. 10.1093/ije/dyr120 22158673 PMC3235021

[GR275994WANC12] Delaneau O, Marchini J, Zagury J-F. 2012. A linear complexity phasing method for thousands of genomes. Nat Methods 9: 179–181. 10.1038/nmeth.1785 22138821

[GR275994WANC13] Delaneau O, Zagury J-F, Marchini J. 2013. Improved whole-chromosome phasing for disease and population genetic studies. Nat Methods 10: 5–6. 10.1038/nmeth.2307 23269371

[GR275994WANC14] Durbin R. 2014. Efficient haplotype matching and storage using the positional Burrows–Wheeler transform (PBWT). Bioinformatics 30: 1266–1272. 10.1093/bioinformatics/btu014 24413527 PMC3998136

[GR275994WANC15] Efron B, Gong G. 1983. A leisurely look at the bootstrap, the jackknife, and cross-validation. Am Stat 37: 36–48. 10.1080/00031305.1983.10483087

[GR275994WANC16] Falush D, Stephens M, Pritchard JK. 2003. Inference of population structure using multilocus genotype data: linked loci and correlated allele frequencies. Genetics 164: 1567–1587. 10.1093/genetics/164.4.1567 12930761 PMC1462648

[GR275994WANC17] Fenner JN. 2005. Cross-cultural estimation of the human generation interval for use in genetics-based population divergence studies. Am J Phys Anthropol 128: 415–423. 10.1002/ajpa.20188 15795887

[GR275994WANC18] Hellenthal G, Busby GBJ, Band G, Wilson JF, Capelli C, Falush D, Myers S. 2014. A genetic atlas of human admixture history. Science 343: 747–751. 10.1126/science.1243518 24531965 PMC4209567

[GR275994WANC19] Henn B, Gignoux CR, Jobin M, Granka J, Macpherson J, Kidd J, Rodríguez-Botigué L, Ramachandran S, Hon L, Brisbin A, 2011. Hunter-gatherer genomic diversity suggests a southern African origin for modern humans. Proc Natl Acad Sci 108: 5154–5162. 10.1073/pnas.1017511108 21383195 PMC3069156

[GR275994WANC20] Hodoğlugil U, Mahley RW. 2012. Turkish population structure and genetic ancestry reveal relatedness among Eurasian populations. Annals Hum Genet 76: 128–141. 10.1111/j.1469-1809.2011.00701.x PMC4904778 22332727

[GR275994WANC21] Humphreys K, Grankvist A, Leu M, Hall P, Liu J, Ripatti S, Rehnström K, Groop L, Klareskog L, Ding B, 2011. The genetic structure of the Swedish population. PLoS One 6: e22547. 10.1371/journal.pone.0022547 21829632 PMC3150368

[GR275994WANC22] The International HapMap 3 Consortium. 2010. Integrating common and rare genetic variation in diverse human populations. Nature 467: 52–58. 10.1038/nature09298 20811451 PMC3173859

[GR275994WANC23] Kerminen S, Havulinna AS, Hellenthal G, Martin AR, Sarin A-P, Perola M, Palotie A, Salomaa V, Daly M, Ripatti S, 2017. Fine-scale genetic structure in Finland. G3 (Bethesda) 7: 3459–3468. 10.1534/g3.117.300217 28983069 PMC5633394

[GR275994WANC24] Lawson DJ, Hellenthal G, Myers S, Falush D. 2012. Inference of population structure using dense haplotype data. PLoS Genet 8: e1002453. 10.1371/journal.pgen.1002453 22291602 PMC3266881

[GR275994WANC25] Leslie S, Winney B, Hellenthal G, Davison D, Boumertit A, Day T, Hutnik K, Royrvik EC, Cunliffe B, Wellcome Trust Case Control Consortium 2, 2015. The fine-scale genetic structure of the British population. Nature 519: 309–314. 10.1038/nature14230 25788095 PMC4632200

[GR275994WANC26] Li JZ, Absher DM, Tang H, Southwick AM, Casto AM, Ramachandran S, Cann HM, Barsh GS, Feldman M, Cavalli-Sforza LL, 2008. Worldwide human relationships inferred from genome-wide patterns of variation. Science 319: 1100–1104. 10.1126/science.1153717 18292342

[GR275994WANC27] Loh P-R, Lipson M, Patterson N, Moorjani P, Pickrell JK, Reich D, Berger B. 2013. Inferring admixture histories of human populations using linkage disequilibrium. Genetics 193: 1233–1254. 10.1534/genetics.112.147330 23410830 PMC3606100

[GR275994WANC28] López S, Tarekegn A, Band G, van Dorp L, Bird N, Morris S, Oljira T, Mekonnen E, Bekele E, Blench R, 2021. Evidence of the interplay of genetics and culture in Ethiopia. Nat Commun 12: 3581. 10.1038/s41467-021-23712-w 34117245 PMC8196081

[GR275994WANC29] Mattingsdal M, Ebenesersdóttir SS, Moore KHS, Andreassen OA, Hansen TF, Werge T, Kockum I, Olsson T, Alfredsson L, Helgason A, 2021. The genetic structure of Norway. Eur J Hum Genet 29: 1710–1718. 10.1038/s41431-021-00899-6 34002043 PMC8560852

[GR275994WANC30] Metspalu M, Romero IG, Yunusbayev B, Chaubey G, Mallick CB, Hudjashov G, Nelis M, Mägi R, Metspalu E, Remm M, 2011. Shared and unique components of human population structure and genome-wide signals of positive selection in south Asia. Am J Hum Genet 89: 731–744. 10.1016/j.ajhg.2011.11.010 22152676 PMC3234374

[GR275994WANC31] Moorjani P, Patterson N, Hirschhorn JN, Keinan A, Hao L, Atzmon G, Burns E, Ostrer H, Price AL, Reich D. 2011. The history of African gene flow into Southern Europeans, Levantines, and Jews. PLoS Genet 7: e1001373. 10.1371/journal.pgen.1001373 21533020 PMC3080861

[GR275994WANC32] Novembre J, Johnson T, Bryc K, Kutalik Z, Boyko AR, Auton A, Indap A, King KA, Bergman S, Nelson MR, 2008. Genes mirror geography within Europe. Nature 456: 98–101. 10.1038/nature07331 18758442 PMC2735096

[GR275994WANC33] Patterson N, Moorjani P, Luo Y, Mallick S, Rohland N, Zhan Y, Genschoreck T, Webster T, Reich D. 2012. Ancient admixture in human history. Genetics 192: 1065–1093. 10.1534/genetics.112.145037 22960212 PMC3522152

[GR275994WANC34] Pickrell JK, Patterson N, Loh P-R, Lipson M, Berger B, Stoneking M, Pakendorf B, Reich D. 2014. Ancient west Eurasian ancestry in southern and eastern Africa. Proc Natl Acad Sci 111: 2632–2637. 10.1073/pnas.1313787111 24550290 PMC3932865

[GR275994WANC35] Pool JE, Nielsen R. 2009. Inference of historical changes in migration rate from the lengths of migrant tracts. Genetics 181: 711–719. 10.1534/genetics.108.098095 19087958 PMC2644959

[GR275994WANC36] Price AL, Tandon A, Patterson N, Barnes KC, Rafaels N, Ruczinski I, Beaty TH, Mathias R, Reich D, Myers S. 2009. Sensitive detection of chromosomal segments of distinct ancestry in admixed populations. PLoS Genet 5: e1000519. 10.1371/journal.pgen.1000519 19543370 PMC2689842

[GR275994WANC37] Purcell S, Neale B, Todd-Brown K, Thomas L, Ferreira MAR, Bender D, Maller J, Sklar P, de Bakker PIW, Daly M, 2007. PLINK: a toolset for whole-genome association and population-based linkage analyses. Am J Hum Genet 81: 559–575. 10.1086/519795 17701901 PMC1950838

[GR275994WANC38] Rasmussen M, Li Y, Lindgreen S, Pedersen JS, Albrechtsen A, Moltke I, Metspalu M, Metspalu E, Kivisild T, Gupta R, 2010. Ancient human genome sequence of an extinct Palaeo-Eskimo. Nature 463: 757–762. 10.1038/nature08835 20148029 PMC3951495

[GR275994WANC39] Raveane A, Aneli S, Montinaro F, Athanasiadis G, Barlera S, Birolo G, Boncoraglio G, Di Blasio A, Di Gaetano C, Pagani L, 2019. Population structure of modern-day Italians reveals patterns of ancient and archaic ancestries in Southern Europe. Sci Adv 5: eaaw3492. 10.1126/sciadv.aaw3492 31517044 PMC6726452

[GR275994WANC40] Saag L, Laneman M, Varul L, Malve M, Valk H, Razzak MA, Shirobokov IG, Khartanovich VI, Mikhaylova ER, Kushniarevich A, 2019. The arrival of Siberian ancestry connecting the eastern Baltic to Uralic speakers further east. Curr Biol 29: 1701–1711.e16. 10.1016/j.cub.2019.04.026 31080083 PMC6544527

[GR275994WANC41] Saint Pierre A, Giemza J, Alves I, Karakachoff M, Gaudin M, Amouyel P, Dartigues J, Tzourio C, Monteil M, Galan P, 2020. The genetic history of France. Eur J Hum Genet 28: 853–865. 10.1038/s41431-020-0584-1 32042083 PMC7316781

[GR275994WANC42] Salter-Townshend M, Myers S. 2019. Fine-scale inference of ancestry segments without prior knowledge of admixing groups. Genetics 212: 869–889. 10.1534/genetics.119.302139 31123038 PMC6614886

[GR275994WANC43] Sawcer S, Hellenthal G, Pirinen M, Spencer C, The International Multiple Sclerosis Genetics Consortium, The Wellcome Trust Case Control Consortium. 2011. Genetic risk and a primary role for cell-mediated immune mechanisms in multiple sclerosis. Nature 476: 214–219. 10.1038/nature10251 21833088 PMC3182531

[GR275994WANC44] Van den Eynden J, Descamps T, Delporte E, Roosens N, De Keersmaecker S, De Wit V, Vermeesch J, Goetghebeur E, Tafforeau J, Demarest S, 2018. The genetic structure of the Belgian population. Hum Genomics 12: 6. 10.1186/s40246-018-0136-8 29394955 PMC5796395

[GR275994WANC45] Veeramah K, Rott A, Groß M, van Dorp L, López S, Kirsanow K, Sell C, Blöcher J, Wegmann D, Link V, 2018. Population genomic analysis of elongated skulls reveals extensive female-biased immigration in early medieval Bavaria. Proc Natl Acad Sci 115: 3494–3499. 10.1073/pnas.1719880115 29531040 PMC5879695

[GR275994WANC46] Wangkumhang P, Hellenthal G. 2018. Statistical methods for detecting admixture. Curr Opin Genet Dev 53: 121–127. 10.1016/j.gde.2018.08.002 30245220

[GR275994WANC47] Yunusbayev B, Metspalu M, Järve M, Kutuev I, Rootsi S, Metspalu E, Behar D, Varendi K, Sahakyan H, Khusainova R, 2012. The Caucasus as an asymmetric semipermeable barrier to ancient human migrations. Mol Biol Evol 29: 359–365. 10.1093/molbev/msr221 21917723

