# Supplementary material for: An efficient method to identify, date, and describe admixture events using haplotype information
Source: Genome Res. 2022 Aug;32(8):1553–64. doi: 10.1101/gr.275994.121 (PMC9435750; doi:10.1101/gr.275994.121)
Supplement: Supplemental Material [file supp_32_8_1553__DC1.html]

An efficient method to identify, date, and describe admixture events using haplotype information — Supplemental Material 

# An efficient method to identify, date, and describe admixture events using haplotype information

## Supplemental Material

- Supplemental\_Globetrotter.tar.gz
- Supplementary\_Information.pdf
