# Supplementary material for: An efficient method to identify, date, and describe admixture events using haplotype information
Source: Genome Res. 2022 Aug;32(8):1553–64. doi: 10.1101/gr.275994.121 (PMC9435750; doi:10.1101/gr.275994.121)
Supplement: Supplemental Material [file supp_gr.275994.121_Supplemental_Globetrotter.tar.gz › FastGLOBETROTTER_Instructions.pdf]

Instruction Manual for "fastGLOBETROTTER: an efficient method to identify, date and describe admixture events using haplotype information"

Pongsakorn Wangkumhang & Garrett Hellenthal

August 13, 2021

Contents

1 Introduction **2**

2 Running ChromoPainterv2 to make input files for fastGLOBETROTTER **2**

3 Getting started **3**

4 Input Files **4**
- 4.1 parameter_infile 4
  - 4.1.1 input.file.ids 6
  - 4.1.2 input.file.copyvectors 7
  - 4.1.3 bootstrap re-samples versus jack-knifing 7
- 4.2 painting_samples_filelist_infile 7
- 4.3 recom_rates_filelist_infile 8

5 Output **8**
- 5.1 [output.filename1].txt 8
- 5.2 **[output.filename1]_curves.txt** 11
- 5.3 **[output.filename1].pdf** 13
- 5.4 [output.filename2].txt 13

6 Example usage of fastGLOBETROTTER **13**

7 Computational time and memory **14**

8 Citation **14**

1 Introduction

fastGLOBETROTTER is a program for inferring and dating admixture in populations, which was built following GLOBETROTTER [1], but with better performance features. The program provides faster inference without loss of accuracy, making it particularly suitable for large-scale data, e.g. hundreds of individuals and hundreds of thousands to millions of Single-Nucleotide-Polymorphisms (SNPs). To describe each admixing event within a target population, fastGLOBETROTTER uses genetic information from multiple sampled reference groups, or "surrogates", that may be related to ancestral sources of the target population. In particular fastGLOBETROTTER identifies whether the target population descends from multiple sources that intermixed at one or more times in the past, with each such source described as a mixture of the sampled surrogates provided by the user. It also infers the date(s) of such admixture, allowing inference of up to two admixture events.

The steps to perform fastGLOBETROTTER inference are similar to those for GLOBETROTTER, with all details provided below. In these instructions, a "target" population refers to the sampled population tested for admixture, and "surrogate" refers to the sampled populations that represent potential sources of ancestry in the target. "Donors" and "recipients" refer to inference from the companion program ChromoPainterv2 [2], with "donors" the sampled populations used to describe haplotype patterns in the "recipients". Here ChromoPainterv2 should be used to paint each "target" and "surrogate" population (i.e. as recipients) using a set of "donor" populations. In addition to these instructions, we have provided a tutorial that describes a specific, recommended analysis protocol that goes through these analysis steps.

2 Running ChromoPainterv2 to make input files for fastGLOBETROTTER

Prior to fastGLOBETROTTER, users need to perform ChromoPainterv2 analyses to obtain two files:

1. "a copying vector" file – a ChromoPainterv2 *XXX.chunklengths.out* output file produced by painting all surrogate and target individuals conditional on a set of donor individuals.

2. "painting samples" file – a ChromoPainterv2 *XXX.samples.out* output file produced by painting all target individuals conditional on a set of donor individuals. The set of donor individuals should ideally be the same set as that used in (1). (However, in practice, if testing multiple targets using the same dataset, ChromoPainterv2 usually can be run allowing all individuals to copy from each other by using that program's '-a' switch.) Most critically, the target population should not be included among the donors, as doing so will mask admixture signals.

3 Getting started

To install the program, first extract the files in the .tar ball and then compile using the following command:

```
R CMD SHLIB -o fastGLOBETROTTERCompanion.so fastGLOBETROTTERCompanion.c
-lz
```

Note that you must have “zlib” installed (e.g. sudo apt-get install zlib1g-dev).

You must also have the package “nnls” installed in R (i.e. install.packages(“nnls”)).

Note also that in order to run fastGLOBETROTTER on your machine, you may need to change the line in *fastGLOBETROTTER.R* that reads dyn.load(“fastGLOBETROTTERCompanion.so”) to include the pathway directory, i.e. dyn.load(“/directorypath/fastGLOBETROTTERCompanion.so”).

The fastGLOBETROTTER command line is as follows:

```
R < fastGLOBETROTTER.R [parameter_infile] [painting_samples_filelist_infile]
[recom_rate_filelist_infile] [running_mode] --no-save > [screen_output]
```

There are 4 required command parameters:

1. **parameter_infile**: contains a description of all the parameters to use in fastGLOBETROTTER (see Section 4.1).

2. **painting_samples_filelist_infile**: contains a list of the *XXX.samples.out* files (e.g. one file per chromosome) from a ChromoPainterv2 analysis of the target population conditional on the donors (see Section 4.2).

3. **recom_rate_filelist_infile**: contains a list of the recombination rate files used when running ChromoPainterv2. The file order must identical to the chromosome order in **painting_samples_filelist_infile**. (See Section 4.3.)

4. **running_mode**: specifies one of four different fastGLOBETROTTER modes:

   (a) “mem” - calculate memory (RAM) required by fastGLOBETROTTER in modes 1-3 described below, and print this memory to the screen output

   (b) “1” - run at maximum speed while using maximal memory (RAM)

   (c) “2” - run at maximum speed while requiring less memory than mode 1, though at a potential loss of accuracy (currently not recommended)

   (d) “3” - run at a slower speed, while requiring less memory (typically <1G).

Type “`R < fastGLOBETROTTER help --no-save`” to get a brief description of this command line and the parameter input file options.

4 Input Files

fastGLOBETROTTER takes three files as input, each having nearly identical formats as those used in GLOBETROTTER [1]:

4.1 parameter_infile

The parameter file (see example in “*tutorial/paramfile.txt*”) contains 20 rows. Below is a description of the parameters in each of these rows, which need to be formatted (and ordered) as shown in bold type, with brackets containing allowed values. Many of these parameters deal with how to fit the so-called “coancestry curves” described in [1], which measure the decay of admixture linkage disequilibrium versus the distance between SNPs. These curves are constructed for all pairwise combinations of surrogate populations inferred to match >**props.cutoff** (see below) of the total ancestry of the target population.

- **prop.ind: [0,1]** - indicate whether (“1”) or not (“0”) to infer admixture proportions, dates and sources (if “0”, this information will be read from previously made fastGLOBETROTTER files specified by save.file.main)
- **bootstrap.date.ind: [0,1,2]** - “1” to perform bootstrap re-sampling to infer confidence intervals around date estimates, “2” to instead perform jackknife re-sampling, and “0” for no action
- **null.ind: [0,1]** - indicate whether to standardize by a “NULL” individual when performing inference (recommended; this is used for inferring p-values for evidence of admixture and is also appropriate when the “target” population has likely undergone bottleneck effects and general robustness testing)
- **input.file.ids: [input.filename1]** - pathway and name for file containing id labels for all samples, for the ChromoPainterv2 analysis run to make the *XXX.samples.out* files (see below)
- **input.file.copyvectors: [input.filename2]** - pathway and name for file containing copy vectors for all surrogate and target populations (see below)
- **save.file.main: [output.filename1]** - pathway and name (prefix) for main output file
- **save.file.bootstraps: [output.filename2]** - pathway and name (prefix) for inferred date bootstrap/jackknife output file

- **copyvector.popnames: [pop 1 pop 2 ... pop k]** - names of all k populations used as donors; i.e. that both surrogate and target populations copied from when running ChromoPainterv2 (NOTE: Any painted segments in the *XXX.samples.out* files that select the target population as a donor will be ignored, even if you include the target population in this line of the input file.)

- **surrogate.popnames: [pop 1 pop 2 ... pop j]** - names of all j surrogate populations, i.e. used to describe admixture in target.popname

- **target.popname: [pop rec]** - name of target population

- **num.mixing.iterations: [0,1,...,5,...]** - number of iterations of date and proportion/source estimation to perform; "0" specifies to only infer proportions of ancestry relating the target to each surrogate as in [3], and to not try and infer/date admixture events (only used when prop.ind: 1)

- **props.cutoff: [0.0,...,1.0]** - at each iteration, remove any surrogates that contribute $\leq$ this value to the mixture describing the target population

- **bootstrap.num: [0,1,...]** - number of bootstrap re-samples (only used when bootstrap.date.ind: 1)

- **num.admixdates.bootstrap: [1,2]** - number of dates to fit when performing bootstrap/jackknife resampling (only used when bootstrap.date.ind: 1 or 2)

- **num.surrogatepops.perplot: [1,...]** - will plot this number squared of coancestry curves for each page of the curves output file (only used when prop.ind: 1)

- **curve.range: [lower.lim upper.lim]** - lower and upper bounds of x-axis (i.e. cM distance between DNA segments) to fit dates to when generating coancestry curves

- **bin.width: [e.g. 0.1]** - width of x-axis bins (in cM) when generating coancestry curves

- **xlim.plot: [lower.lim upper.lim]** - lower and upper bounds (in cM) of x-axis to plot for coancestry curves (only used when prop.ind: 1)

- **prop.continue.ind: [0,1]** - indicate whether you are continuing proportion estimation from those in a previous file (in which case the previous file will be read from save.file.main and output files will add the suffix "_continue")

- **haploid.ind: [0,1]** - indicate whether individuals are haploid ("1") or diploid ("0")

4.1.1 input.file.ids

This file should match exactly the donor input file used in ChromoPainterv2 ('-t' switch) when generating the *XXX.samples.out* files. An example of **input.file.ids** is provided in "*tutorial/individual.txt*". Each row is ordered to match the rows of the genotype input file ('-g') run using ChromoPainterv2. There are three columns per row, with the first column giving the individual identifier, the second column giving the individual's population label and the third column an indicator for whether the individual is not included in the analysis (use "0" to specify NOT to include the given individual). For example, consider a file with the following 7 individuals:

```
IND1 Pop1 0
IND3 Pop1 1
IND2 Pop1 1
IND4 Pop2 1
IND5 Pop2 0
Pop4Ind1 Pop4 1
IND7 Pop1 1
```

Here we only are specifying to include individuals {IND3, IND2, IND4, Pop4Ind1, IND7}, while excluding {IND1,IND5}.

Each population label specified in **copyvector.popnames**, **surrogate.popnames** and **target.popname** of the file "parameter_infile" MUST be in column 2 of at least one row of the file **input.file.ids**. An exception, incorporated to make things more flexible for the user, is if all **surrogate.popnames** and **target.popname** labels missing from column 2 of input.file.ids are specified in the row labels of input.file.copyvectors, and similarly all **copyvector.popnames** missing from column 2 of **input.file.ids** are specified in the column labels of **input.file.copyvectors**. In other words, the column names and row names of input.file.copyvectors must contain the individual identifiers and/or the population labels.

It is critical that the order of individuals in **input.file.ids** corresponds to the donor indices used in the *XXX.samples.out* files used in the analysis. Each painting sample (row) of each *XXX.samples.out* file gives a number D for each SNP, corresponding to the row of the ChromoPainterv2 haplotype input file ('-g' switch) that contains the donor haplotype copied at that SNP (where the first haplotype in this input file is assigned $D = 1$). The row containing the label for this donor individual in **input.file.ids** MUST be row D/p (with any decimal values of D/p rounded up to the nearest integer) where $p = 1, 2$ is the ploidy of the organism.

4.1.2 input.file.copyvectors

This file should contain the *XXX.chunklengths.out* file from the corresponding ChromoPainterv2 analyses, in the same format and combined across all chromosomes and individuals. Each row is an individual (or population), and the columns give the total amount of genome-wide DNA that the given individual (or population) is inferred to copy from every "donor" individual (or population) in the corresponding ChromoPainterv2 analyses. An example of **input.file.copyvectors** is provided in "*tutorial/copyvector.txt*".

The first row of **input.file.copyvectors** lists the column labels reflecting the donor individuals and/or populations. The remaining rows of **input.file.copyvectors** list the "recipient" individual (or population) label in the first column, with the remaining columns containing the total amount (or proportion) of genome-wide DNA that the given recipient individual (or population) copies from each donor label provided in the first row.

4.1.3 bootstrap re-samples versus jack-knifing

The parameter **bootstrap.date.ind** in `parameter_infile` is used to specify whether to calculate confidence intervals around date estimates using bootstrap re-sampling (if **bootstrap.date.ind: 1**) or jack-knifing (if **bootstrap.date.ind: 2**). If bootstrapping is chosen, **bootstrap.num** bootstrap re-samples of target individuals' chromosomes will be performed to infer new date estimates. We recommend using **bootstrap.num: 100**. Confidence intervals can be calculated using quantiles of these new inferred dates.

If jack-knifing is chosen, date estimation will be performed using all files except one in the `painting_samples_filelist_infile` input file. Each file in `painting_samples_filelist_infile` is dropped in this manner, so that the number of new date estimates will be equal to the number of rows in `painting_samples_filelist_infile`. These jack-knife values can then be used to generate standard errors using the procedure described in e.g. [4].

Standard errors using jack-knifing will likely give larger confidence intervals than using bootstrap re-sampling. For this reason, we recommend using bootstrap re-sampling rather than jack-knifing, except in cases where bootstrap re-sampling cannot be performed because the number of target individuals is small (e.g. $\leq$3 individuals or so).

4.2 painting_samples_filelist_infile

This file contains a list of file locations and names of *XXX.samples.out* output files from ChromoPainterv2, specifying one file per line (see *tutorial/samplefile.txt*).

Each XXX.samples.out file contains an initial row that gives details of the ChromoPainterv2) run (note that the number of samples MUST be listed in the

21st column of this file, exactly as it is by ChromoPainterv2). The remaining rows give the labels and the painting samples inferred for each haplotype of each painted recipient individual, including those from the target population, giving the sample number in the first column and the index of the donor haplotype copied at each SNP in the remaining columns.

4.3 recom_rates_filelist_infile

This file is a list of file locations and names of the recobination rate files used when running ChromoPainterv2 ('-r' switch) to make the *XXX.samples.out* files. The chromosome order must correspond to the order listed in the painting_samples_filelist_infile (See *tutorial/recomfile.txt*.)

Each file listed in "*recom_rates_filelist_infile*" should contain a header line followed by one line for each SNP. Each line should contain two columns, with the first column denoting the basepair position value, in increasing order. The second column should give the genetic distance per basepair between the SNP at the position in the first column of the same row and the SNP at the position in the first column of the subsequent row. The last row should have a "0" in the second column (though this is not required – this value is simply ignored by the program). Genetic distance should be given in Morgans, or at least the relevant output files assume this value is in Morgans.

5 Output

Identical to GLOBETROTTER [1], there are four output files from fastGLOBETROTTER:

5.1 [output.filename1].txt

This file summarizes the inferred admixture proportions, dates and sources. The first line lists our "best-guess" conclusion for admixture in the target population. The conclusion can be:

- **uncertain** - admixture is detected but difficult to describe (technical details: combined fit quality for two events "fit.quality.2events" < 0.985)
- **one-date** - a single date of admixture between two sources (combined fit quality for two events ≥ 0.985; two-date score "maxScore.2events" <0.35; fit-quality for a single event "fit.quality.1event" ≥ 0.975)
- **one-date-multiway** - a single date of admixture between more than two sources (combined fit quality for two events ≥ 0.985; two-date score < 0.35; fit quality for a single event < 0.975)

- **multiple-dates** - two (or more) distinct dates of admixture between two or more sources (combined fit quality for two events $\geq$ 0.985; two-date score $\geq$ 0.35)

- **unclear signal – check curves/bootstraps** – when fitting two dates of admixture, no coancestry curve (red line) provided a very good fit to the data (black lines), suggesting the admixture signal – if any – is very unclear or nonexistent (the maximum two-date R^2 fit across all curves is <0.3, so "maxR2fit.1date" should also be low). In these cases, date estimates when bootstrapping may contain 1 or $\geq$ 400 when specifying **null.ind: 1**, indicating no clear evidence of admixture.

We caution that these are just guidelines, and that **we highly recommend careful visual exploration of the inferred coancestry curves provided in the .pdf output file** to see how well e.g. one versus two events fit the data. Furthermore, we recommend that if any bootstrap re-sample gives a date estimate of 1 or very high values (e.g. >400), this indicates that there is no clear evidence of admixture.

The next lines ("1-DATE FIT EVIDENCE, DATE ESTIMATE, SINGLE BEST-FITTING DONORS") provide the fastGLOBETROTTER inferred date, proportions and "best-guess" sources of admixture for a single event or multiway admixture when assuming only a single date of admixture (i.e. this information is particularly appropriate when the "best-guess" conclusion is "one-date" or "one-date-multiway"), as well as measures of "goodness-of-fit" for these events. Specifically:

- **gen.1date** - inferred date of admixture in generations from present

- **proportion.source1** - inferred proportion of admixture from the minority contributing source

- **maxR2fit.1date** - the goodness-of-fit (R2) for a single date of admixture, taking the maximum value across all inferred coancestry curves

- **fit.quality.1event** - the fit of a single admixture event

- **fit.quality.2events** - the fit of the first and the second admixture event

- **bestmatch.event1.source1** - the single "best-guess" surrogate population that matches the inferred minority contributing source

- **bestmatch.event1.source2** - the single "best-guess" surrogate population that matches the inferred majority contributing source

- **proportion.event2.source1** - inferred proportion of admixture from the minority contributing source for the second, less strongly signaled event (appropriate for "one-date-multiway")

- **bestmatch.event2.source1** - the single "best-guess" surrogate population that matches the inferred minority contributing source for the second, less strongly signaled event (appropriate for "one-date-multiway")

- **bestmatch.event2.source2** - the single "best-guess" surrogate population that matches the inferred majority contributing source for the second, less strongly signaled event (appropriate for "one-date-multiway")

The subsequent lines of output ("2-DATE FIT EVIDENCE, DATE ESTIMATES, SINGLE BEST-FITTING DONORS") give 2 inferred admixture dates, proportions and "best-guess" sources of admixture when assuming two distinct dates of admixture. These lines are most appropriate if the "best-guess" conclusion is "multiple-dates", or if any curves in the *pdf output file indicate two-dates of admixture (red line) is a better fit to the data than one-date (green line). In particular:

- **gen.2dates.date1** - inferred date of admixture (in generations from present) for the first (most strongly signaled) event, when assuming two dates

- **gen.2dates.date2** - inferred date of admixture (in generations from present) for the second event, when assuming two dates

- **maxScore.2events** - the additional goodness-of-fit (R2) explained by adding a second date versus assuming only a single date of admixture, taking the maximum such value across all inferred coancestry curves

- **proportion.date1.source1** - inferred proportion of admixture from the minority contributing source for the first dates event (when assuming two dates)

- **bestmatch.date1.source1** - the single "best-guess" surrogate population that matches the inferred minority contributing source for the first dates event (when assuming two dates)

- **bestmatch.date1.source2** - the single "best-guess" surrogate population that matches the inferred majority contributing source for the first dates event (when assuming two dates)

- **proportion.date2.source1** - inferred proportion of admixture from the minority contributing source for the second dates event (when assuming two dates)

- **bestmatch.date2.source1** - the single "best-guess" surrogate population that matches the inferred minority contributing source for the second dates event (when assuming two dates)

- **bestmatch.date2.source2** - the single "best-guess" surrogate population that matches the inferred majority contributing source for the second dates event (when assuming two dates)

The subsequent lines of output ("1-DATE FIT SOURCES, PC1") present the fastGLOBETROTTER inferred composition of each admixing source in the most strongly signaled event, when assuming only a single date of admixture. In particular every two consecutive rows describe the inferred genetic composition of one admixing source (i.e. where each source is described as a mixture of the sampled surrogate groups), giving both the proportion of DNA contributed by that source (first column), and fastGLOBETROTTER's inferred mixture coefficients to describe each source (remaining columns - these should sum to 1 for each source). For instance, consider the following output:

```
##########################
### 1-DATE FIT SOURCES, PC1:
proportion BantuKenya Mandenka BantuSouthAfrica
0.29 0.26 0.31 0.43
proportion Han Japanese Balochi Druze Sardinian Ireland English
0.71 0.01 0.01 0.02 0.03 0.03 0.16 0.74
##########################
```

This implies that fastGLOBETROTTER has inferred the first admixing source, which is inferred to contribute 29% of the DNA found in the target population, to be best represented genetically as a mixture of (0.26, 0.31, 0.43) times the copy vectors of surrogate labels {BantuKenya, Mandenka, BantuSouthAfrica}, respectively. And fastGLOBETROTTER has inferred the second admixing source, which contributes 71% of the DNA of the target population, to be best represented genetically as a mixture of (0.01, 0.01, 0.02, 0.03, 0.03, 0.16, 0.74) times the copy vectors of surrogate labels {Han, Japanese, Balochi, Druze, Sardinian, Ireland, English}, respectively. Similar source proportion and mixing coefficient inference is given next for the less strongly signaled event, when assuming a single date of admixture (i.e. "1-DATE FIT SOURCES, PC2"), which is particularly appropriate when the best-guess conclusion is "one-date-multiway".

Following this is the analogous inference for the first date's event when assuming two dates of admixture ("2-DATE FIT SOURCES, DATE1-PC1"), and the second date's event when assuming two dates of admixture ("2-DATE FIT SOURCES, DATE2-PC1"), which is particularly appropriate when the "best-guess" conclusion is "multiple- dates".

5.2 [output.filename1]_curves.txt

This output file, with prefix specified by **save.file.main** in "*parameter_infile*" and suffix **"_curves.txt"**, gives the coancestry curves for every pairwise combination of surrogate populations inferred to match >**props.cutoff** (see "*parameter_infile*") of the total ancestry of the target population, as well as information related to these curves. It is generated only if **prop.ind: 1** in "*parameter_infile*".

The first line of **[output.filename1]_curves.txt** gives our "best-guess" conclusion for admixture in the target population (see Section 5.1). The second line gives a header key, and the genetic distance (in cM) corresponding to the x-axis of each coancesty curve (see Section 4.1 and [1] for a description of how the bins and range for these cM distance values are specified).

The first two columns ("surrogate1", "surrogate2") denote the surrogate populations in a given pair.

The third column ("curve.description") describes each curve; of which there are the following four types per surrogate population pairing (output in consecutive rows):

1. **scaled.data** – the re-weighted counts of DNA segment pairs inferred to copy from surrogate populations surrogate1 and surrogate2; i.e. the (re-weighted) "data"
2. **gen.fit.1date** – GLOBETROTTER's inferred fitted line for a single date of admixture
3. **source.fit.1date** – GLOBETROTTER's inferred fitted line for a single date of admixture between only two sources
4. **gen.fit.2date** – GLOBETROTTER's inferred fit for two distinct dates of admixture

The fourth column ("rsquared.date.fit") gives the goodness-of-fit (R^2) for a single admixture date for **gen.fit.1date** and **source.fit.1date** (note this column has identical values for these two rows within a given surrogate pair) or for two dates for **gen.fit.2date**.

The fifth column ("intercept.fit") and sixth column ("intercept.fit.date2") give the coefficients from fitting **scaled.data** using as predictors one or two exponential distributions with rates equal to the cM bins given in the second line scaled by the inferred dates of admixture. For **gen.fit.1date** and **gen.fit.2date**, this fit is accomplished using linear regression, while for **source.fit.1date** the coefficient is determined using the inferred admixture proportions and source mixing coefficients for the most strongly signaled event assuming a single date of admixture (see [1] for details). (Note that the sixth column is "NA" for **gen.fit.1date** and **source.fit.1date**, as they only have a single inferred date and hence a single predictor and coefficient.)

The remaining columns for each row give the y-axis values, corresponding to each x-axis cM bin value given in the second line, for each respective curve. In particular these y-axis values give the (scaled) probability of copying surrogate1 and surrogate2 at a pair of DNA segments separated by the corresponding x-axis (i.e. cM distance) value, for the raw data or one of the fitted models. For each of 1-4

above, these values are plotted for each pairing of surrogate populations in the pdf file described in Section 5.3.

5.3 [output.filename1].pdf

This output file, with prefix specified by **save.file.main** in "*parameter_infile*" and suffix **".pdf"**, plots the coancestry curves for every pairwise combination of surrogate populations inferred to match >**props.cutoff** (see "*parameter_infile*") of the total ancestry of the target population. The given surrogate pairing is specified in each plot's title. The x-axis gives genetic distance in cM (see Section 4.1 and [1] for a description of how the bins and range for these cM distance values are specified). The y-axis gives the weighted probability of copying from the first and second surrogate populations listed in the title at a pair of DNA segments separated by the corresponding x-axis (cM distance) value. For each surrogate population pair, four such probabilities (lines) are shown, corresponding to 1-4 in Section 5.2 with the colors black, green, blue and red, respectively. It is generated only if **prop.ind: 1** in "*parameter_infile*".

5.4 [output.filename2].txt

This output file, with prefix specified by **save.file.bootstraps** in "parameter_infile" and suffix ".txt", gives the inferred dates and goodness-of-fit (R2) values for bootstrap/jackknife re-samples of individuals DNA (see above for details of column values).

6 Example usage of fastGLOBETROTTER

To determine which mode to run fastGLOBETROTTER in, type:

```
R < fastGLOBETROTTER.R tutorial/paramfile.txt tutorial/samplefile.txt
tutorial/recomfile.txt mem --no-save > output.out
```

This will take a moment to finish and will output (in "output.out") the calculated memory required by each mode of fastGLOBETROTTER (see Section 3). This will assist users to decide a suitable mode according to their computational resources.

To run with mode 1, which is the fastest setting, type:

```
R < fastGLOBETROTTER.R tutorial/paramfile.txt tutorial/samplefile.txt
tutorial/recomfile.txt 1 --no-save > output.out
```

Once the program finishes, the output files will be produced in the directory specified in `tutorial/paramfile.txt`.

7 Computational time and memory

Assume N target population individuals, C chromosomes, B bootstrap/jackknife re-samples, M mixing iterations, S painting samples, L SNPs (maximum across all chromosomes), J donor populations, K surrogate groups, $I(\leq SL)$ total "chunks" (i.e. the maximum number of "chunks" across chromosomes and individuals), $I_j(I)$ "chunks" copied from donor population j (the maximum number of "chunks" across chromosomes and individuals copied from a single donor population) and G grid points over which the coancestry curves are estimated (i.e. $G=$ (**curve.range(upper.lim)-curve.range(lower.lim)**)/**bin.width** in section 4.1). Then the computational complexity of fastGLOBETROTTER at the maximum speed is:

$$O[(B+M)(NC(SL+J^2I+I_j^2)+NGJ^2K^2)+C[min(N;100)]2(L+I_j^2)]$$

The maximum required memory for Mode 1 and 2 is $O(NGJ^2+NGK^2)$, while Mode 3 stores $O(NGK^2)$.

8 Citation

When making use of fastGLOBETROTTER, please cite the following (or a more recent version):

Wangkumhang P, Greenfield M, and Hellenthal G (2021) "An efficient method to identify, date and describe admixture events using haplotype information" *BioRxiv* doi:10.1101/2021.08.12.455263

Questions? Bugs? Please contact Pongsakorn Wangkumhang (pongsakornw@gmail.com) and Garrett Hellenthal (ghellenthal@gmail.com).

References

[1] G. Hellenthal, G.B.J. Busby, G. Band, J.F. Wilson, C. Capelli, D. Falush, and S. Myers. A genetic atlas of human admixture history. *Science*, 343:747–751, 2014.

[2] D.J. Lawson, G. Hellenthal, S. Myers, and D. Falush. Inference of population structure using dense haplotype data. *PLoS Genet*, 8(1):e1002453, 2012.

[3] S. Leslie, B. Winney, G. Hellenthal, D. Davison, A. Boumertit, T. Day, K. Hutnik, E.C. Royrvik, B. Cunliffe, Wellcome Trust Case Control Consortium 2, International Multiple Sclerosis Genetics Consortium, D.J. Lawson, D. Falush, C. Freeman, M. Pirinen, S. Myers, M. Robinson, P. Donnelly, and W. Bodmer. The fine scale genetic structure of the British population. *Nature*, 519:309–314, 2015.

[4] F.M.T.A. Busing, E. Meijer, and R. Van Der Leeden. Delete-m Jackknife for Unequal m. *Statistics and Computing*, 9:3–8, 1999.
