# Supplementary material for: An efficient method to identify, date, and describe admixture events using haplotype information
Source: Genome Res. 2022 Aug;32(8):1553–64. doi: 10.1101/gr.275994.121 (PMC9435750; doi:10.1101/gr.275994.121)
Supplement: Supplemental Material [file supp_gr.275994.121_Supplemental_Globetrotter.tar.gz › FastGLOBETROTTER_Tutorial.pdf]

Tutorial for “fastGLOBETROTTER: an efficient method to identify, date and describe admixture events using haplotype information”

Pongsakorn Wangkumhang & Garrett Hellenthal

August 13, 2021

Contents

1 Introduction **1**

2 Pre-processing steps for CHROMOPAINTER **2**

3 Running CHROMOPAINTER **2**
- 3.1 CHROMOPAINTER files for fastGLOBETROTTER 4
- 3.2 Estimating parameters required by CHROMOPAINTER 5
- 3.3 Generating the copy vector input file 6
- 3.4 Generating the painting samples files 6

4 Inferring admixture events using fastGLOBETROTTER **7**
- 4.1 fastGLOBETROTTER `paramfile.txt` 7
- 4.2 Running fastGLOBETROTTER 8
- 4.3 fastGLOBETROTTER output 9

5 Citation **11**

1 Introduction

The aim of this tutorial is to walk users through the steps in inferring an admixture event using the two programs CHROMOPAINTER [1] and fastGLOBETROTTER. We will analyse a target population of 100 individuals simulated as mixtures of present-day French (contributing ≈50% of the DNA) and Yoruban (contributing the remaining ≈50%) sampled individuals, with the mixture occurring 30 generations ago. We will attempt to infer admixture using the worldwide populations in Table 1 as reference populations to describe the admixture event. For simplicity, we will focus only on analysing chromosomes 21-22.

In this tutorial, a “target” population refers to the sampled population tested for admixture (i.e. the simulated population mentinoed above). To describe this

event, we use "surrogate" populations, which are the sampled populations that represent potential sources of ancestry in the target. When running CHROMOPAINTER, "recipients" refer to the individuals we paint, and "donors" refer to the individuals each recipient is painted against. I.e. CHROMOPAINTER composes (stochastically) each phased recipient haploid as a mosaic of haplotypes from the phased donor haploids. Running fastGLOBETROTTER requires painting each surrogate and target population haploid using the same set of donor individuals. Often in practice, as in this tutorial, the donors consist of all surrogate population individuals.

2 Pre-processing steps for CHROMOPAINTER

While there are no example files for this process as part of this tutorial, data must first be phased and put into CHROMOPAINTER input format. For example, PLINK [3] can be used to combine files, run quality control and split data into separate chromosomes (ideal if datasets are large, with e.g. >100K SNPs). Then the data can be phased using (e.g.) SHAPEIT [4] with the following command:

```
shapeit --input-bed [bedfile] [bimfile] [famfile] -M [genticmapfile]
--output-max [phasedfile] [samplefile]
```

The SHAPEIT output files can be formatted into CHROMOPAINTER input using `impute2chromopainter2.pl` included in the fastGLOBETROTTER package (also available at https://people.maths.bris.ac.uk/ madjl/finestructure/toolsummary.html):

```
perl impute2chromopainter2.pl [phasedfile] [genticmapfile] [output]
```

This will give the input files included as part of this tutorial, with e.g. `AllFrenchYoruba30gen50propchrZ.txt` containing the phased haplotypes of chromosome Z for all populations, and `ChromZ.recomrates` containing the corresponding recombination rates for chromosome Z.

3 Running CHROMOPAINTER

First open CHROMOPAINTER:

```
tar -xzvf ChromoPainterv2.tar.gz
```

and then compile:

```
gcc -o ChromoPainterv2 ChromoPainterv2.c -lm -lz
```

Population	Region	Number of individuals
Balochi	Central South Asia	21
Balochi	Central South Asia	21
BantuKenya	Africa	11
BantuSouthAfrica	Africa	8
Druze	West Asia	42
EastSicilian	South Europe	10
English	Northwest Europe	6
Hadza	Central Africa	3
Han	Southeast Asia	34
Ireland	Northeest Europe	7
Japanese	Northeast Asia	28
Makrani	Central South Asia	22
Mandenka	West Africa	22
Maya	America	21
Naxi	Southeast Asia	8
Russian	East Europe	25
Sardinian	South Europe	28
Saudi	South Middle East	10
Scottish	Northwest Europe	6
She	Southeast Asia	10
Sindhi	Central South Asia	23
Surui	America	8
Syrian	South Middle East	16
Turkish	West Asia	17
UAE	South Middle East	9
Uygur	Central South Asia	10
Yemeni	South Middle East	4
FrenchYoruba	simulated population	100

Table 1: List of populations used to describe admixture in the "FrenchYoruba" simulated target population, for this tutorial. These populations were analysed in [2].

There are two fastGLOBETROTTER inputs required from CHROMOPAINTER: (1) a copy vector file and (2) a painting sample files. Here are the steps to obtain those files.

3.1 CHROMOPAINTER files for fastGLOBETROTTER

When inferring admixture in a target population, fastGLOBETROTTER requires two CHROMOPAINTER input files.

A. One (Section 3.3) is used to infer the sources of an admixture event(s), by using genome-wide patterns of haplotype sharing.

B. The other (Section 3.4) is to date the admixture event(s), by using local matching to different putative ancestral sources along the genome of each target individual.

As detailed in this section, file (A) requires painting the target population and each surrogate population, while file (B) requires painting only the target population. Ideally (A) and (B) should be painted using identical sets of donor populations, as we do here.

For simplicity, in many applications surrogate populations are often the same as donor populations. One important complication of this is that surrogates and targets are not painted using an identical set of donors. In particular a person is not allowed to use themself as a donor. Thus if population X with n_X individuals is treated as both a surrogate and donor population, individuals from surrogate population X will only match to $n_x - 1$ donor individuals from X. In contrast, individuals from the target population and each other surrogate population will match to all n_x donor individuals from X. This asymmetry can affect downstream fastGLOBETROTTER inference.

A strategy to cope with this is to paint the target and all surrogate populations using $n_k - 1$ individuals from each donor population k that is also a surrogate population. To do so, when painting people from the target population, a new CHROMOPAINTER '-t' file can be made that removes one person from each surrogate population that is also a donor population, by placing a "0" in the third column of that person's row. Analogously, when painting people from surrogate population k, a new CHROMOPAINTER '-t' file can be made that removes one person from each *other* surrogate population that is also a donor population.

However, for convenience, in practice this asymmetry issue is ignored and no special painting is done to account for the fact that some surrogate populations are also donor populations. For example, we do not account for this issue in this tutorial. As we demonstrate below, fastGLOBETROTTER inference is still accurate, for this tutorial's particular admixture scenario and sampled populations and counts. The reason for this is in part to do with our observation that the total amount matched to all individuals of a donor population with n_k individuals is often approximately the same as that matched to $n_k - 1$ individuals;

that is, only the amount matched to each individual changes. **However, we stress this may not always be the case.** Unusual fastGLOBETROTTER results, such as bootstrap-based 95% confidence intervals around date estimates not containing the point date estimate, may indicate this shortcut should not be taken.

3.2 Estimating parameters required by CHROMOPAINTER

We first need to estimate two parameters required in CHROMOPAINTER: the switch (-n) and emission (-M) rates. To do so, for computational convenience we run ChromoPainterv2 on only a subset of chromosomes and "recipient" individuals (i.e. individuals that are painted). It is critical that the number of "donors" you use (i.e. individuals that each "recipient" is painted against) is the same as you will use in the final CHROMOPAINTER analysis. The command for estimating n and M on chromosome 21 is:

```
ChromoPainterv2 -g AllFrenchYoruba30gen50propchr21.txt -r Chrom21.recomrates
-t individual.txt -f popfileSurr.txt 1 10 -s 0 -i 10 -in -iM -o output_estimateEM_Chr21
```

The file `popfileSurr.txt` specifies that we want to paint all populations in Table 1 as recipients ("R"), while using all of these populations except the target population "FrenchYoruba" as donors ("D") in this painting. We do this because we need to paint each surrogate and target population individual. For convenience, we use each surrogate population also as a donor population. Here "-f popfileSurr.txt 1 10" specifies to only paint each phased haploid of the first ten recipient individuals (as ordered in `AllFrenchYoruba30gen50propchr21.txt`) using individuals from the donor populations. (Though note an individual cannot match to themselves as a donor.) We set "-s 0" to specify that no painting samples are needed in this step, since these take up storage and will not be used. And "-i 10 -in -iM" specifies to use 10 iterations of Expectation-Maximisation to infer the switch ("-in") and emission ("-iM") parameters. We might apply the command above to other individuals (e.g. "-f popfileSurr.txt 101 110", "-f popfileSurr.txt 201 210", etc – typically we paint 10 out of every 100 individuals) and chromosomes (e.g. typically we do chromosomes 1, 8, 15, 22). After completing the above command, and repeating an analogous command for chromosome 22 (i.e. replace `21` with `22` in the above), we apply a perl script to summarize these inferred values across individuals:

```
perl ChromoPainterv2EstimatedNeExtractEM.pl
```

Note that prior to running the above perl script, you must specify which chromosomes were painted (in `@chromovec`), and the number of SNPs per chromosome (in `@chromolengths`). You should also provide a prefix name (in `$infilePREFIX`) and a suffix name (in `$infileSUFFIX`) for the CHROMOPAINTER output files to be read in, where the value between the prefix and suffix is the chromosome number.

From the output of this perl script, which is printed to the screen, we obtain n = 407.590 and M = 0.000441. We use these values below.

3.3 Generating the copy vector input file

To construct the copy vectors, we use the estimated parameters from Section 3.2 and run ChromoPainterv2 again, this time painting all individuals as recipients on chromosome 21:

```
ChromoPainterv2 -g AllFrenchYoruba30gen50propchr21.txt -r Chrom21.recomrates
-t individual.txt -f popfileSurr.txt 0 0 -s 0 -n 407.590 -M 0.000441
-o AllFrenchYoruba30gen50propchr21_DonorvALL
```

The command "-f poplistSurr.txt 0 0" specifies that we paint all individuals from the populations listed in `popfileSurr.txt` as recipients. Repeat the above for each chromosome (i.e. chromosome 22 in this tutorial). We then want to sum the `AllFrenchYoruba30gen50propchrZ_DonorvALL.chunklengths.out` files across all chromosomes, which we can do using a provided perl script:

```
perl ChromoPainterOutputSum.pl AllFrenchYoruba30gen50propchr _DonorvALL.chunklengths.out
```

Note that prior to running the above perl script, you must specify which chromosomes were painted (in `@chromovec`). The above will make a new file "`AllFrenchYoruba30gen50propchrAll_DonorvALL.chunklengths.out`" that will be used in the fastGLOBETROTTER `paramfile.txt` input file.

(Note that to save computational time, you could remove the "FrenchYoruba" population as a recipient for the above ChromoPainterv2 run, since it will be painted as a recipient in Section 3.4. However, you would then have to merge the `xxx.chunklengths.out` output files from the above painting and Section 3.4 paintings prior to using `ChromoPainterOutputSum.pl`.)

3.4 Generating the painting samples files

Here we paint only the target population individuals, as these output files will be used by fastGLOBETROTTER to date the admixture event, which relies on inferred local matching to different surrogate populations along each admixed individual's genome. (Note that `fastGLOBETROTTER` will remove segments in these files that are matched to other target individuals, potentially removing a lot of data, so that it is critical that you do <u>not</u> include target individuals as donors when generating these painting samples files.) This painting is specified in `popfile.txt`, which now specifies only "FrenchYoruba" as a recipient ("R"). The command for generating painting samples for chromosome 21 is as follows:

```
ChromoPainterv2 -g AllFrenchYoruba30gen50propchr21.txt -r Chrom21.recomrate
-t individual.txt -f popfile.txt 0 0 -s 10 -n 407.590 -M 0.000441 -o
AllFrenchYoruba30gen50propchr21_DonorvTarget
```

We also keep "-s 10" (the default), which specifies to provide ten stochastic painting samples for each target population haploid. Repeat the above for each chromosome.

4 Inferring admixture events using fastGLOBETROTTER

In this section, we will apply fastGLOBETROTTER to infer the admixture history of the target population (FrenchYoruba) using the other 26 reference populations in Table 1 as surrogates for the admixing sources. First extract fastGLOBETROTTER (now making sure you are in the directory above the `tutorial` directory):

```
tar -xzvf fastGLOBETROTTER.tar.gz
```

and compile the program:

```
R CMD SHLIB -o fastGLOBETROTTERCompanion.so fastGLOBETROTTERCompanion.c
-lz
```

Note you may need add a new path to a line in `fastGLOBETROTTER.R` that reads dyn.load("fastGLOBETROTTERCompanion.so") to dyn.load("/directorypath/ fastGLOBETROTTERCompanion.so").

To run fastGLOBETROTTER, you have to prepare three files:

- a parameter file, `tutorial/paramfile.txt`, that contains the fastGLOBETROTTER parameters, including which population is the target, and which populations should be specified as donors and surrogates, etc.
- a painting samples file, `tutorial/samplefile.txt`, that lists the file locations of `tutorial/AllFrenchYoruba30gen50propchrZ_DonorvTarget.samples.out.gz` of all included chromosomes, generated in Section 3.4. Note these files can also each be gzipped, and have suffix `*.gz`.
- a recombination rate file, `tutorial/recomfile.txt`, that lists the file locations of `tutorial/ChromXX.recomrates` of all included chromosomes.

4.1 fastGLOBETROTTER `paramfile.txt`

See the fastGLOBETROTTER manual for a description of all parameters in `tutorial/paramfile.txt`. We describe a few of the key ones here. We specify

“prop.ind: 1” to note that we wish to infer and date admixture, and “bootstrap.date.ind: 1” to also perform bootstrap re-samples to infer confidence intervals around the inferred date. In this tutorial, we use “bootstrap.num: 100” such re-samples, which is what we recommend. Importantly, here fastGLOBETROTTER will assume a single date of admixture for each bootstrap re-sample. If you wish to infer confidence intervals around dates when instead assuming two dates of admixture, you must change “num.admixdates.bootstrap: 2” in `tutorial/paramfile.txt`.

To account for linkage disequilibrium patterns that may not be due to genuine admixture, e.g. if the target population has experienced a strong bottleneck since admixture, we suggest setting “null.ind: 1”, as we have done in this tutorial.

We set “input.file.ids:” as `tutorial/individual.txt`. This file must be the same file as we used by CHROMOPAINTER in Section 3.4 to generate the painting samples files.

The output filename (“save.file.main:”) is defined according to the user’s preference. Here we use `tutorial/AllFrenchYoruba30gen50prop.main.txt`.

We also specify the “input.file.copyvectors:” made in Section 3.3 as `tutorial/AllFrenchYoruba30gen50propchrAll_DonorvALL.chunklengths.out`.

The line “copyvector.popnames:” contains the list of donor populations used in ChromoPainterv2 in Sections 3.3 and 3.4. The “target.popname” target population can be exluded from this – any matching to this population will be treated as 0.

Finally, “surrogate.popnames:” lists the surrogate populations to be used to describe the admixture. Each of these surrogate populations had to be painted (i.e. used as recipients) in the analysis in Section 3.3. Typically “copyvector.popnames” and “surrogate.popsnames” list the same populations, as we have done in this tutorial.

4.2 Running fastGLOBETROTTER

As mentioned, fastGLOBETROTTER allows users to select a suitable “running_mode” that depends on user’s memory (RAM) availability. The fastest mode is “Mode 1” which consumes more memory than other modes, whereas “Mode 3” normally requires minimal memory but is ≈5-50 times slower than Mode 1. We currently do not recommend using Mode 2, which is marginally faster than Mode 1 and requires less memory, but may sacrifice accuracy. To select the desired Mode, users can calculate RAM required for each mode by

performing this command with the "mem" setting:

```
R < fastGLOBETROTTER.R tutorial/paramfile.txt tutorial/samplefile.txt
tutorial/recomfile.txt mem -no-save > tutorial/AllFrenchYoruba30gen50prop.fastGT.out
```

The result of the memory test will be printed in `tutorial/AllFrenchYoruba30gen50prop.fastGT.out`. Users then can choose which Mode suits their available resources. Below is an example of a command line when Mode 1 (recommended if you have sufficient RAM) is selected:

```
R < fastGLOBETROTTER.R tutorial/paramfile.txt tutorial/samplefile.txt
tutorial/recomfile.txt 1 -no-save > tutorial/AllFrenchYoruba30gen50prop.fastGT.out
```

4.3 fastGLOBETROTTER output

When the analysis is finished, the final main inference will be printed in `tutorial/AllFrenchYoruba30gen50prop.main.txt`. In the provided output examples, the admixture conclusion (top line) is "one date" of admixture at around 27 generations; this is given with `gen.1date` near the top of the file. Note that, as fastGLOBETROTTER is stochastic, this inferred date (and all other values described in this section) may slightly change among independent runs. The output file `tutorial/AllFrenchYoruba30gen50prop.boot.txt` gives the 100 bootstrap re-sample values for the inferred date (column 2). If rounding the lower bound down to the nearest integer and the higher bound up to the nearest integer, the inner 95% empirical range of these values is 21-31 in the provided example output, which can be reported as a confidence interval for the inferred date.

Other values in `tutorial/AllFrenchYoruba30gen50prop.main.txt` give details of the inferred admixture event. For example, the inferred sources are described as:

```
###########################
### 1-DATE FIT SOURCES, PC1:
proportion BantuKenya Mandenka BantuSouthAfrica
0.49 0.16292895647841 0.317407291065057 0.519663752456533
proportion Druze Sardinian Ireland English
0.51 0.0196072757431737 0.0658784932171261 0.129113532999502 0.785400698040198
###########################
```

Of the two sources contributing to the target, one is inferred to contribute 49% of the total admixture proportion and is most genetically similar to the sampled BantuSouthAfrica. More specifically, its painting profile is inferred to be best described as {51.97,31.74,16.29}% mixture of the painting profiles for

{BantuSouthAfrica, Mandenka, BantuKenya}, respectively. Analogously, the other source is inferred to contribute 51%, and is most genetically similar to the sampled English. The single best-fitting groups for each admixing source are given near the top of the file (in "`1-DATE FIT EVIDENCE, DATE ESTIMATE, SINGLE BEST-FITTING DONORS`"), under the labels `bestmatch.event1.source1` and `bestmatch.event1.source2`. Note here these are "BantuSouthAfrica" and "Ireland". This inference is close to the true admixture we simulated in terms of the date, ancestral sources and admixture proportions.

If the conclusion had been "one-date, multiway", we can similarly look at the inference under "`1-DATE FIT SOURCES, PC2`" to see a description of the sources inferred for an additional event that is less strongly-signalled than the first one. Analogously, if the conclusion had been "multiple-dates", you should use the inference under "`2-DATE FIT SOURCES, DATE1-PC1`" to describe the first admixture date (the one whose estimated date is given in `gen.2dates.date1` near the top of the file) and the inference under "`2-DATE FIT SOURCES, DATE2-PC1`" to describe the second admixture date (the one whose estimated date is given in `gen.2dates.date2`).

The `tutorial/AllFrenchYoruba30gen50prop.main.pdf` file provides the plots of fitted "coancestry curves" for each surrogate pair inferred to describe >0.1% (`props.cutoff` in `tutorial/paramfile.txt`) of the target population's haplotype patterns. Examples are shown in Figure 1. **It is essential to visually inspect these plots** to assess whether the inference proposed by the fastGLOBETROTTER model is supported by the data. In particular, a subset of curves should be clearly increasing and a subset clearly decreasing. In addition, if a curve involving a pair of surrogates is increasing, this suggests that the given pair of surrogates represent different ancestral sources. For example, English and Mandenka in Figure 1-left show this patterns, which makes sense given the former represents the "French" source and the latter represents the "Yoruba" source. Conversely, if the curve is decreasing, this suggests the surrogates are each representing the same ancestral source, such as plotting Mandenka and BantuSouthAfrica in Figure 1-right, which again makes sense since each represents the "Yoruba" source. These patterns can be cross-referenced with the results reported in `tutorial/AllFrenchYoruba30gen50prop.main.txt` to assess their reliability. In the provided example, the two inferred sources (see "`1-DATE FIT SOURCES, PC1`" above) are clearly differentiated into African and European surrogate populations, which fits the patterns presented in the curves of `tutorial/AllFrenchYoruba30gen50prop.main.pdf`.

Finally, the inferred admixture conclusion (i.e. "one-date", "one-date, multiway", "multiple-dates") can be double-checked by assessing how well the {green, cyan, red} colored lines fit the data (black line). Specifically, the green line shows the fit of a single date of admixture, and the red lines shows the fit of two dates of admixture. If the red line fits the data (black line) substantially better than the green line for some (non-noisy) curves, this suggests two dates of admixture

may be more plausible than one-date, and that the one date admixture inference may be inaccurate. Meanwhile, the cyan line shows the fit of a single date of admixture between two sources. If in some (non-noisy) curves the cyan line goes in the opposite direction of the data (black line), i.e. the cyan line increases while the black line decreases or vice versa, this suggests perhaps more than two sources intermixed at the same (or multiple) date(s). In this case (Figure 1, and for other curves in `tutorial/AllFrenchYoruba30gen50prop.main.pdf`), the black and cyan lines do not show this trend, and the red line is not a noticeably better fit to the data than the green line. This suggests that the conclusion of one date of admixture between two sources, the truth, is a good fit to the data.

5 Citation

When making use of fastGLOBETROTTER, please cite the following (or a more recent version):

Wangkumhang P, Greenfield M, and Hellenthal G (2021) "An efficient method to identify, date and describe admixture events using haplotype information" *BioRxiv* doi:10.1101/2021.08.12.455263

Questions? Bugs? Please contact Pongsakorn Wangkumhang (pongsakornw@gmail.com) and Garrett Hellenthal (ghellenthal@gmail.com).

References

[1] D.J. Lawson, G. Hellenthal, S. Myers, and D. Falush. Inference of population structure using dense haplotype data. *PLoS Genet*, 8(1):e1002453, 2012.

[2] G. Hellenthal, G.B.J. Busby, G. Band, J.F. Wilson, C. Capelli, D. Falush, and S. Myers. A genetic atlas of human admixture history. *Science*, 343:747–751, 2014.

[3] S. Purcell, B. Neale, K. Todd-Brown, L. Thomas, M.A.R. Ferreira, D. Bender, J. Maller, P. Sklar, P.I.W. de Bakker, M.J. Daly, and P.C. Sham. PLINK: a toolset for whole-genome association and population-based linkage analysis. *Am J Hum Genet*, 81(3):559–575, 2007.

[4] O. Delaneau, J.F. Zagury, and J. Marchini. Improved whole chromosome phasing for disease and population genetic studies. *Nat Methods*, 10(1):5–6, 2013.

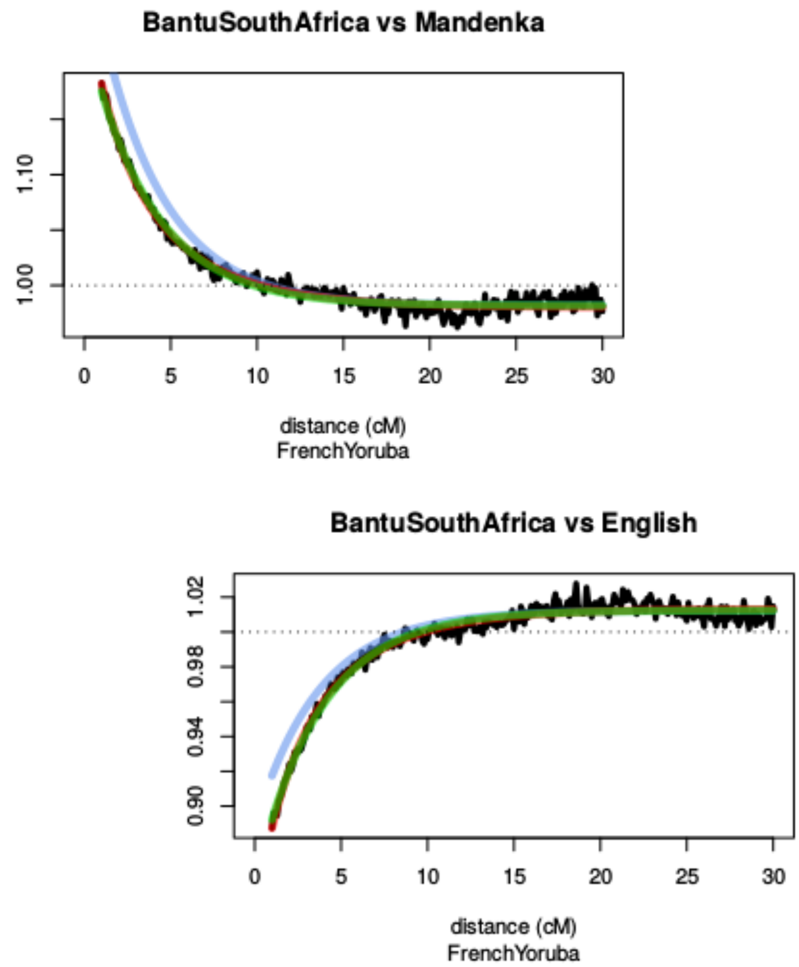

Figure 1: Coancestry curves showing the scaled probability (black lines) that two DNA segments separated by XcM in a target individual match to (top) BantuSouthAfrica and Mandenka surrogates or to (bottom) BantuSouthAfrica and English surrogates. Decreasing curves (top) suggest the two surrogates represent the same admixing source, while increasing curves (bottom) suggest the two surrogates represent different admixing sources. The green line gives the fit of a single date of admixture, which fits the data well here, while the red line (covered by the green) gives the fit of two dates of admixture. The cyan line gives the fit of a single date of admixture between two sources – while this is marginally off of the black line, the cyan line is not opposite to the black line (i.e. the cyan decreasing while the black line increases, or vice versa). This suggests a single date of admixture between two sources provides a good visual fit to the data.
