# Supplementary material for: An efficient method to identify, date, and describe admixture events using haplotype information
Source: Genome Res. 2022 Aug;32(8):1553–64. doi: 10.1101/gr.275994.121 (PMC9435750; doi:10.1101/gr.275994.121)
Supplement: Supplemental Material [file supp_gr.275994.121_Supplemental_Globetrotter.tar.gz › tutorial/AllFrenchYoruba30gen50prop.main.pdf]

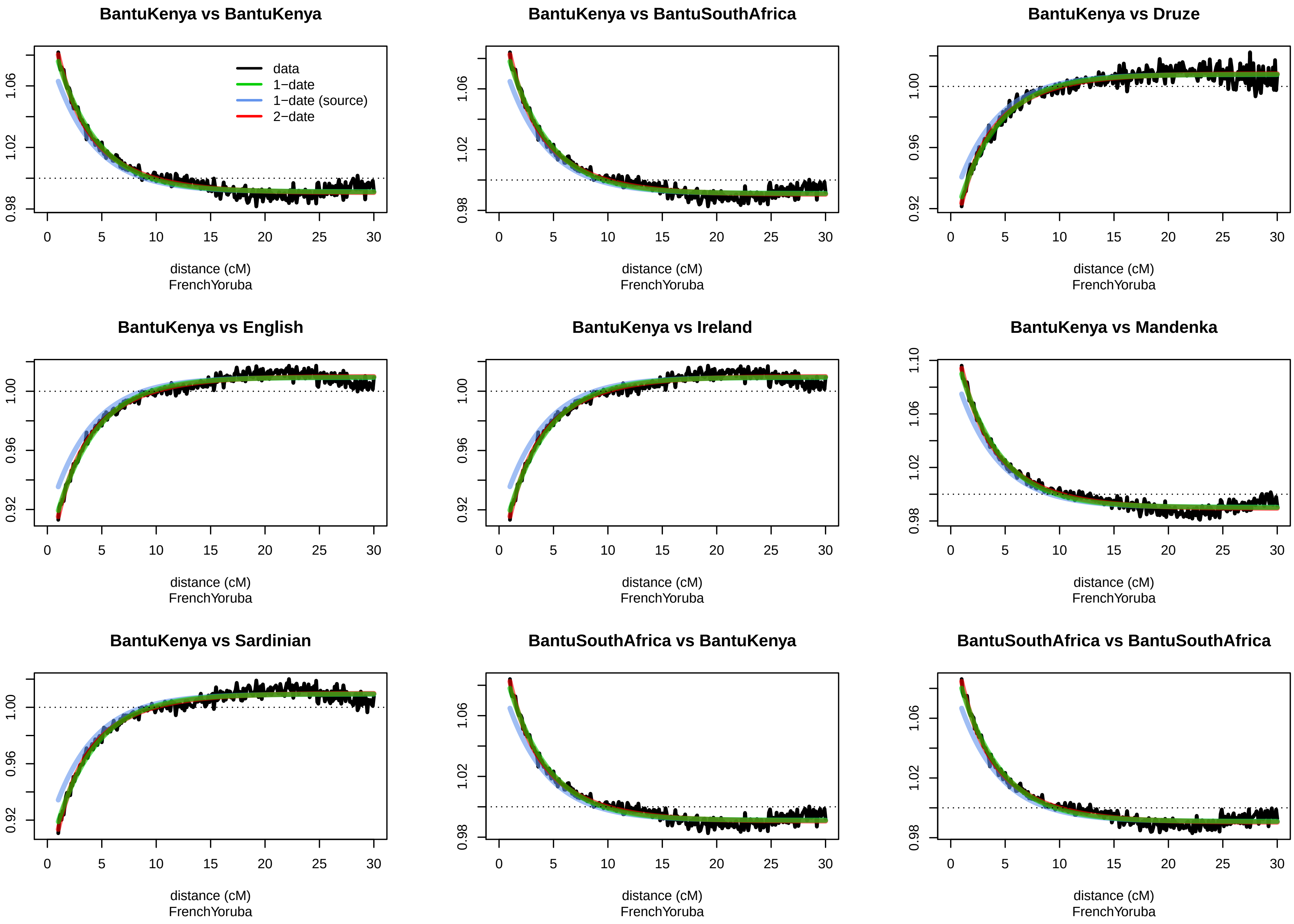
BantuKenya vs BantuKenya
data
1−date
1−date (source)
2−date
0.98
1.02
1.06
0 5 10 15 20 25 30
distance (cM)
FrenchYoruba
BantuKenya vs BantuSouthAfrica
0.98
1.02
1.06
0 5 10 15 20 25 30
distance (cM)
FrenchYoruba
BantuKenya vs Druze
0.92
0.96
1.00
0 5 10 15 20 25 30
distance (cM)
FrenchYoruba
BantuKenya vs English
0.92
0.96
1.00
0 5 10 15 20 25 30
distance (cM)
FrenchYoruba
BantuKenya vs Ireland
0.92
0.96
1.00
0 5 10 15 20 25 30
distance (cM)
FrenchYoruba
BantuKenya vs Mandenka
0.98
1.02
1.06
1.10
0 5 10 15 20 25 30
distance (cM)
FrenchYoruba
BantuKenya vs Sardinian
0.92
0.96
1.00
0 5 10 15 20 25 30
distance (cM)
FrenchYoruba
BantuSouthAfrica vs BantuKenya
0.98
1.02
1.06
0 5 10 15 20 25 30
distance (cM)
FrenchYoruba
BantuSouthAfrica vs BantuSouthAfrica
0.98
1.02
1.06
0 5 10 15 20 25 30
distance (cM)
FrenchYoruba

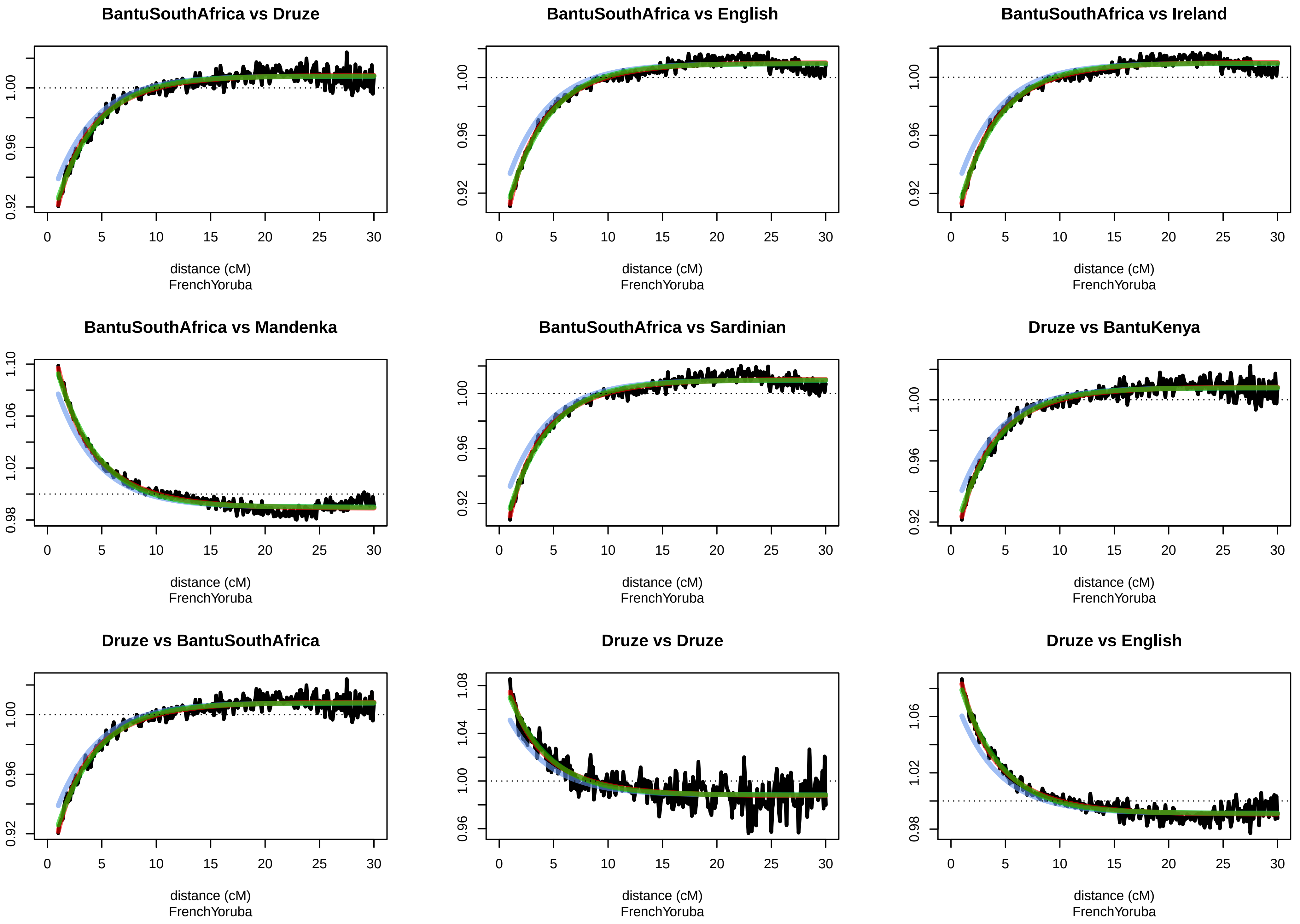
BantuSouthAfrica vs Druze
1.00
0.96
0.92
0
5
10
15
20
25
30
distance (cM)
FrenchYoruba
BantuSouthAfrica vs English
1.00
0.96
0.92
distance (cM)
FrenchYoruba
BantuSouthAfrica vs Ireland
1.00
0.96
0.92
distance (cM)
FrenchYoruba
BantuSouthAfrica vs Mandenka
1.10
1.06
1.02
0.98
distance (cM)
FrenchYoruba
BantuSouthAfrica vs Sardinian
1.00
0.96
0.92
distance (cM)
FrenchYoruba
Druze vs BantuKenya
1.00
0.96
0.92
distance (cM)
FrenchYoruba
Druze vs BantuSouthAfrica
1.00
0.96
0.92
distance (cM)
FrenchYoruba
Druze vs Druze
1.08
1.04
1.00
0.96
distance (cM)
FrenchYoruba
Druze vs English
1.06
1.02
0.98
distance (cM)
FrenchYoruba

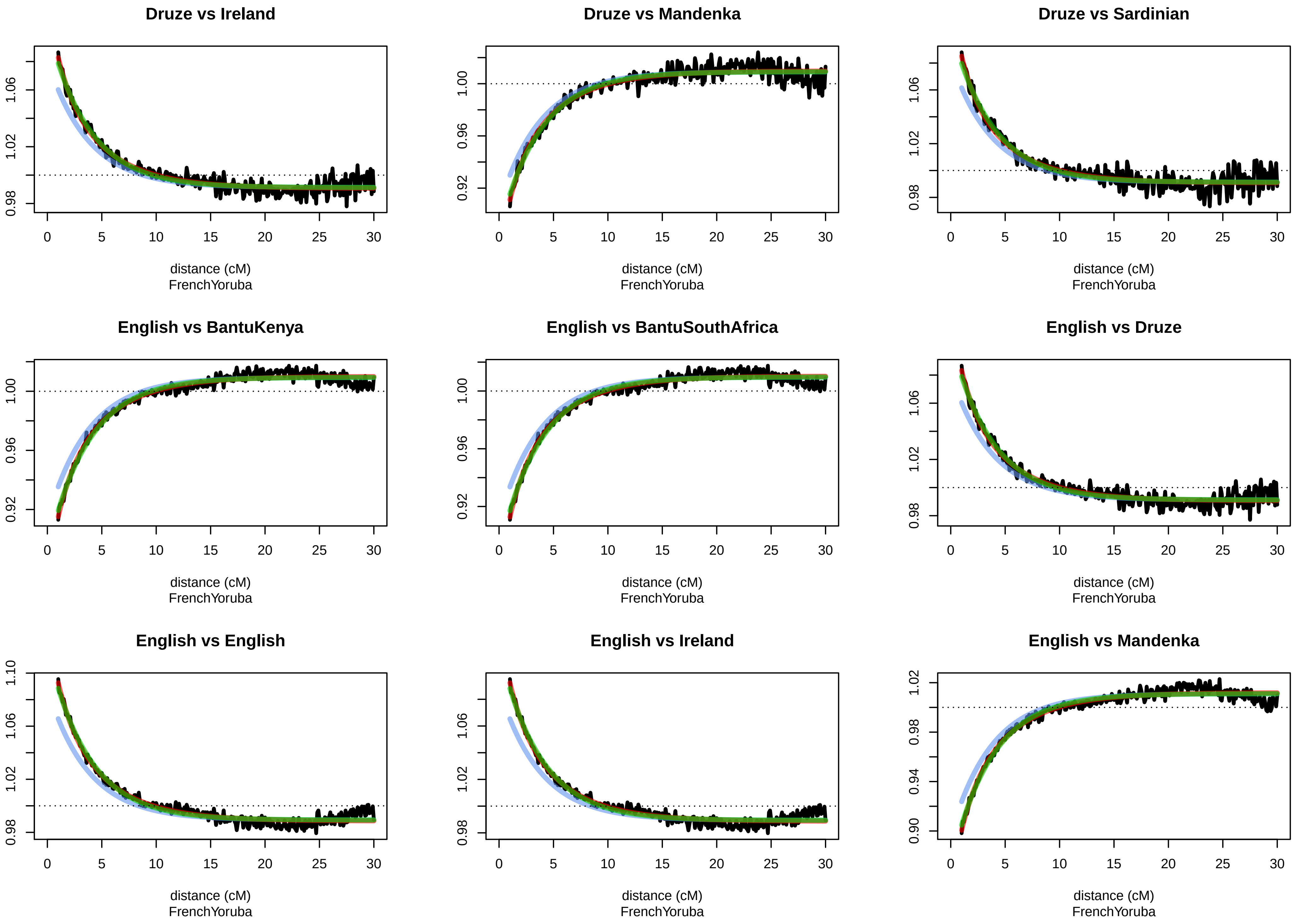

Druze vs Ireland
1.06
1.02
0.98
0
5
10
15
20
25
30
distance (cM)
FrenchYoruba
Druze vs Mandenka
1.00
0.96
0.92
0
5
10
15
20
25
30
distance (cM)
FrenchYoruba
Druze vs Sardinian
1.06
1.02
0.98
0
5
10
15
20
25
30
distance (cM)
FrenchYoruba
English vs BantuKenya
1.00
0.96
0.92
0
5
10
15
20
25
30
distance (cM)
FrenchYoruba
English vs BantuSouthAfrica
1.00
0.96
0.92
0
5
10
15
20
25
30
distance (cM)
FrenchYoruba
English vs Druze
1.06
1.02
0.98
0
5
10
15
20
25
30
distance (cM)
FrenchYoruba
English vs English
1.10
1.06
1.02
0.98
0
5
10
15
20
25
30
distance (cM)
FrenchYoruba
English vs Ireland
1.06
1.02
0.98
0
5
10
15
20
25
30
distance (cM)
FrenchYoruba
English vs Mandenka
1.02
0.98
0.94
0.90
0
5
10
15
20
25
30
distance (cM)
FrenchYoruba

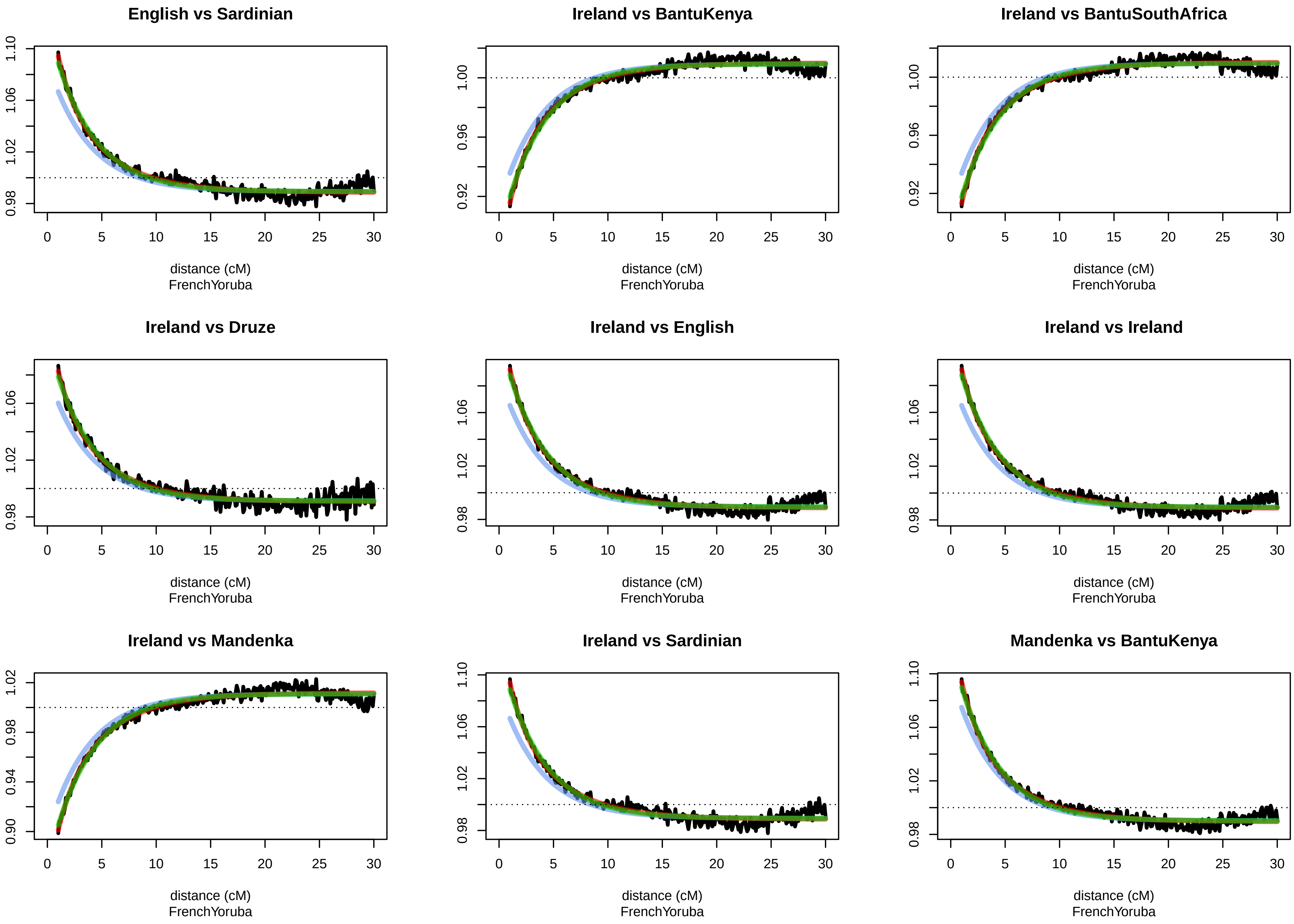
English vs Sardinian
Ireland vs BantuKenya
Ireland vs BantuSouthAfrica
Ireland vs Druze
Ireland vs English
Ireland vs Ireland
Ireland vs Mandenka
Ireland vs Sardinian
Mandenka vs BantuKenya
distance (cM)
FrenchYoruba
0
5
10
15
20
25
30
0.90
0.92
0.94
0.96
0.98
1.00
1.02
1.06
1.10

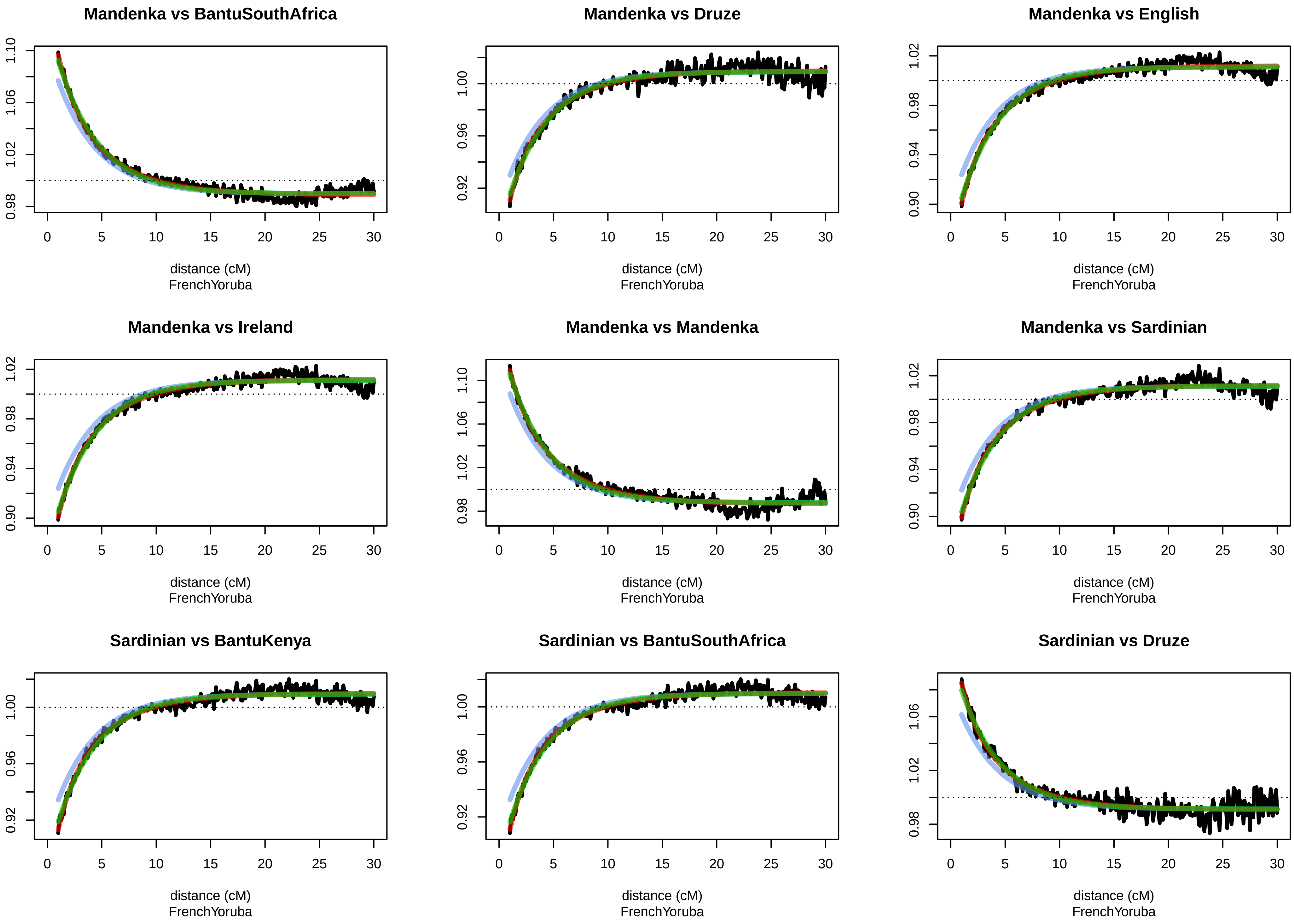
Mandenka vs BantuSouthAfrica
0.98 1.02 1.06 1.10
0 5 10 15 20 25 30
distance (cM)
FrenchYoruba
Mandenka vs Druze
0.92 0.96 1.00
0 5 10 15 20 25 30
distance (cM)
FrenchYoruba
Mandenka vs English
0.90 0.94 0.98 1.02
0 5 10 15 20 25 30
distance (cM)
FrenchYoruba
Mandenka vs Ireland
0.90 0.94 0.98 1.02
0 5 10 15 20 25 30
distance (cM)
FrenchYoruba
Mandenka vs Mandenka
0.98 1.02 1.06 1.10
0 5 10 15 20 25 30
distance (cM)
FrenchYoruba
Mandenka vs Sardinian
0.90 0.94 0.98 1.02
0 5 10 15 20 25 30
distance (cM)
FrenchYoruba
Sardinian vs BantuKenya
0.92 0.96 1.00
0 5 10 15 20 25 30
distance (cM)
FrenchYoruba
Sardinian vs BantuSouthAfrica
0.92 0.96 1.00
0 5 10 15 20 25 30
distance (cM)
FrenchYoruba
Sardinian vs Druze
0.98 1.02 1.06
0 5 10 15 20 25 30
distance (cM)
FrenchYoruba

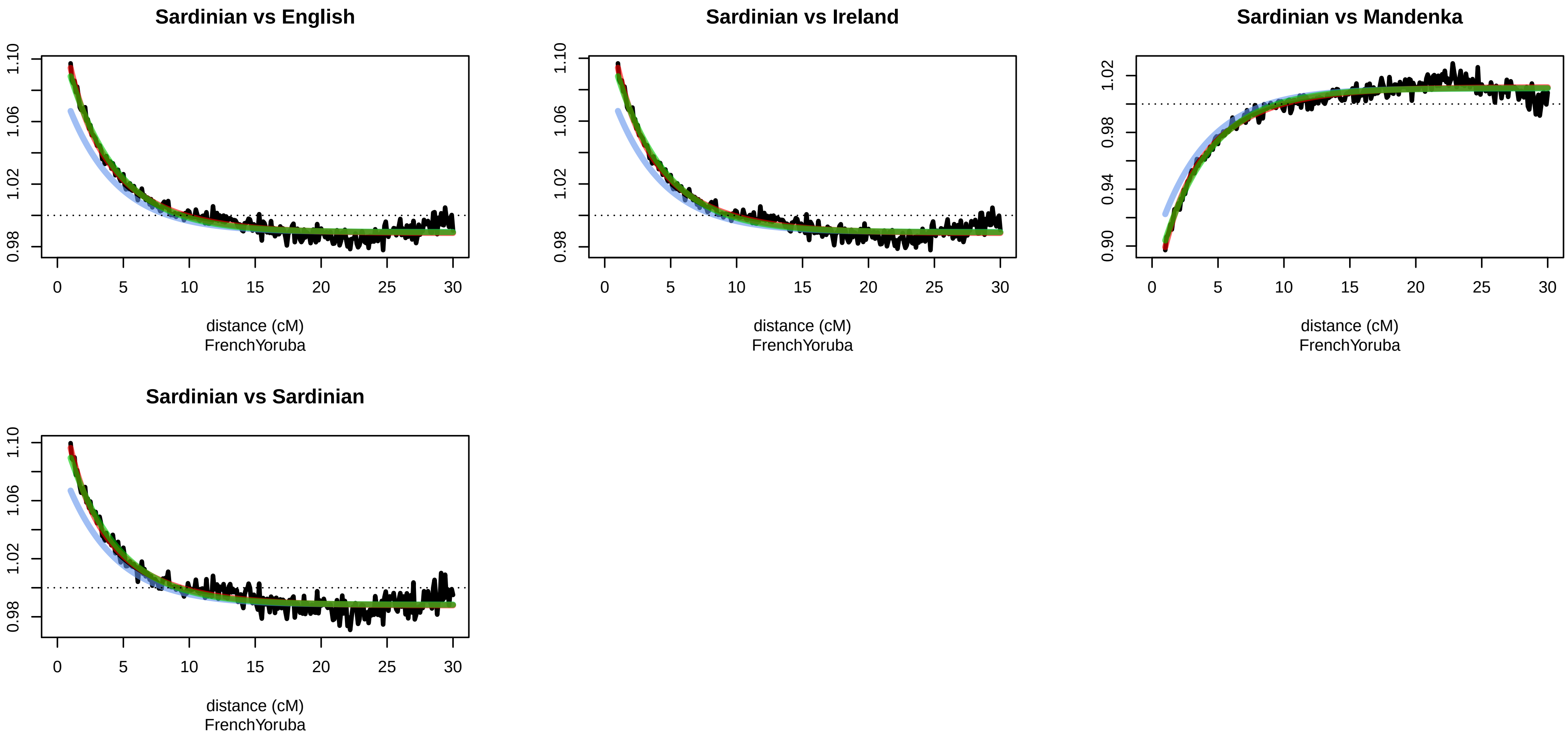
Sardinian vs English
Sardinian vs Ireland
Sardinian vs Mandenka
Sardinian vs Sardinian
0.98
1.02
1.06
1.10
0.90
0.94
0.98
1.02
0
5
10
15
20
25
30
distance (cM)
FrenchYoruba
