# Supplementary material for: An efficient method to identify, date, and describe admixture events using haplotype information
Source: Genome Res. 2022 Aug;32(8):1553–64. doi: 10.1101/gr.275994.121 (PMC9435750; doi:10.1101/gr.275994.121)
Supplement: Supplemental Material [file supp_gr.275994.121_Supplementary_Information.pdf]

Supplementary Information for "An efficient method to identify, date and describe admixture events using haplotype information"

Pongsakorn Wangkumhang[1,2], Matthew Greenfield[1], Garrett Hellenthal[1]

[1]University College London Genetics Institute (UGI), Department of Genetics, Evolution and Environment, University College London, London, United Kingdom, phone: +44(0)2031084003
[2]National Biobank of Thailand, National Science and Technology Development Agency, Pathum Thani, Thailand

Supplementary Tables

sim groups	gen	true %	n	c	R_1	R_2^*	FQ_1	date	%	source 1	source 2
Brahui/Yoruba	7	5	20	1	0.93	0.03	1	9 (8-11)	6	0.009 – BantuK(0.38)BantuSA(0.28)Mandenka(0.25)	0.003 – Balochi(0.85)Makrani(0.15)
Brahui/Yoruba	7	20	20	1	0.96	0.01	1	8 (7-9)	20	0.008 – Mandenka(0.31)BantuK(0.3)BantuSA(0.25)	0.003 – Balochi(0.85)Makrani(0.15)
Brahui/Yoruba	7	50	20	1	0.97	0.02	1	8 (6-9)	48	0.009 – Mandenka(0.33)BantuK(0.3)BantuSA(0.24)	0.003 – Balochi(0.89)Makrani(0.11)
Brahui/Yoruba	30	5	20	1	0.94	0.03	1	30 (28-32)	6	0.008 – BantuK(0.36)Mandenka(0.27)BantuSA(0.22)	0.003 – Balochi(0.89)Makrani(0.11)
Brahui/Yoruba	30	20	20	1	0.95	0.04	1	29 (27-31)	20	0.008 – Mandenka(0.31)BantuK(0.29)BantuSA(0.26)	0.003 – Balochi(0.88)Makrani(0.12)
Brahui/Yoruba	30	50	20	1	0.94	0.01	1	32 (30-34)	47	0.009 – Mandenka(0.34)BantuK(0.3)BantuSA(0.24)	0.003 – Balochi(0.92)Makrani(0.08)
Brahui/Yoruba	150	5	20	1	0.24	0.04	1	117 (87-160)	8	0.009 – Mandenka(0.2)BantuK(0.18)Makrani(0.14)	0.003 – Balochi(0.81)Makrani(0.1)
Brahui/Yoruba	150	20	20	1	0.29	0.04	1	133 (95-159)	22	0.008 – Mandenka(0.25)BantuK(0.22)BantuSA(0.2)	0.003 – Balochi(0.82)Makrani(0.11)
Brahui/Yoruba	150	50	20	1	0.3	0.05	1	129 (102-154)	46	0.009 – Mandenka(0.32)BantuK(0.29)BantuSA(0.23)	0.003 – Balochi(0.76)Makrani(0.1)
Yoruba/French	7	5	20	1	0.98	0.02	1	8 (6-10)	7	0.006 – Lithuanian(0.87)BantuK(0.06)	0.009 – Mandenka(0.32)BantuK(0.28)BantuSA(0.21)
Yoruba/French	7	20	20	1	1	0.03	1	7 (6-9)	25	0.002 – Scottish(0.9)Lithuanian(0.07)	0.009 – Mandenka(0.33)BantuK(0.3)BantuSA(0.22)
Yoruba/French	7	50	20	1	1	0.06	1	7 (6-8)	46	0.009 – Mandenka(0.34)BantuK(0.31)BantuSA(0.23)	0.002 – Welsh(0.42)English(0.17)Scottish(0.11)
Yoruba/French	30	5	20	1	0.97	0.08	1	29 (25-32)	7	0.006 – Lithuanian(0.9)	0.009 – Mandenka(0.31)BantuK(0.28)BantuSA(0.21)
Yoruba/French	30	20	20	1	0.99	0.05	1	30 (28-33)	26	0.002 – Scottish(0.58)Ireland(0.3)Lithuanian(0.08)	0.009 – Mandenka(0.33)BantuK(0.3)BantuSA(0.23)
Yoruba/French	30	50	20	1	0.99	0.13	1	29 (26-31)	47	0.009 – Mandenka(0.33)BantuK(0.3)BantuSA(0.23)	0.002 – Welsh(0.45)Scottish(0.15)NorthItalian(0.11)
Yoruba/French	150	5	20	1	0.54	0.02	0.98	128 (127-153)	12	0.009 – Lithuanian(0.62)BantuK(0.21)BantuSA(0.14)	0.009 – Mandenka(0.33)BantuK(0.25)BantuSA(0.2)
Yoruba/French	150	20	20	1	0.82	0.06	1	154 (128-175)	21	0.002 – Ireland(0.88)Lithuanian(0.11)	0.009 – Mandenka(0.31)BantuK(0.26)BantuSA(0.21)
Yoruba/French	150	50	20	1	0.85	0.07	1	144 (131-172)	46	0.009 – Mandenka(0.32)BantuK(0.29)BantuSA(0.23)	0.002 – Welsh(0.35)Scottish(0.18)Tuscan(0.11)
Colomb/Han	7	5	7	1	0.88	0.05	1	8 (6-10)	25	0.003 – HanNchina(0.63)Saudi(0.07)Maya(0.06)	0.037 – Maya(0.61)Karitiana(0.07)Pima(0.06)
Colomb/Han	7	20	7	1	0.97	0.2	1	6 (5-7)	40	0.001 – Tujia(0.79)	0.037 – Maya(0.6)Karitiana(0.07)Pima(0.06)
Colomb/Han	7	50	7	1	0.98	0.09	1	8 (7-8)	31	0.037 – Maya(0.71)Karitiana(0.09)Pima(0.08)	0.001 – Tujia(0.47)HanNchina(0.11)Tu(0.08)
Colomb/Han	30	5	7	1	0.61	0.12	0.98	18 (16-31)	24	0.037 – Tujia(0.36)Maya(0.14)Scottish(0.12)	0.037 – Maya(0.56)Karitiana(0.07)Pima(0.05)
Colomb/Han	30	20	7	1	0.91	0.05	1	32 (30-34)	37	0.001 – Tujia(0.7)Oroqen(0.06)	0.037 – Maya(0.59)Karitiana(0.07)Pima(0.06)
Colomb/Han	30	50	7	1	0.97	0.27	1	33 (32-34)	31	0.037 – Maya(0.69)Karitiana(0.09)Pima(0.08)	0.001 – Tujia(0.47)HanNchina(0.16)Xibo(0.07)
Colomb/Han†	150	5	7	NA	0.09	0.04	0.95	49 (3-132)	47	0.037 – Maya(0.53)Pima(0.09)	0.037 – Uzbekistani(0.38)Maya(0.29)Dai(0.07)
Colomb/Han†	150	20	7	1	0.14	0.04	0.99	112 (96-224)	41	0.001 – Tujia(0.55)Myanmar(0.19)Karitiana(0.05)	0.037 – Maya(0.57)Pima(0.07)Pathan(0.06)
Colomb/Han†	150	50	7	1	0.34	0.02	1	107 (82-175)	37	0.037 – Maya(0.56)Karitiana(0.07)Scottish(0.06)	0.001 – Tujia(0.3)HanNchina(0.21)Xibo(0.09)
Brahui/Han	7	5	20	1	0.96	0.16	0.99	10 (6-13)	13	0.001 – Tujia(0.8)Dai(0.07)	0.003 – Balochi(0.3)Pathan(0.14)Iranian(0.14)
Brahui/Han	7	20	20	1	0.98	0.34	1	7 (5-9)	30	0.001 – Tujia(0.53)Dai(0.11)Yi(0.06)	0.003 – Balochi(0.35)Makrani(0.11)Sindhi(0.11)
Brahui/Han	7	50	20	1	0.99	0.17	1	9 (8-11)	50	0.001 – Tujia(0.64)Dai(0.11)	0.003 – Balochi(0.41)Makrani(0.1)Armenian(0.07)
Brahui/Han	30	5	20	1	0.92	0.17	0.99	26 (22-34)	14	0.001 – Tujia(0.66)Dai(0.06)Daur(0.06)	0.003 – Balochi(0.35)Pathan(0.13)Sindhi(0.09)
Brahui/Han	30	20	20	1	0.97	0.18	1	32 (27-37)	29	0.001 – Tujia(0.32)Dai(0.18)Miao(0.1)	0.003 – Balochi(0.4)Makrani(0.1)Sindhi(0.1)
Brahui/Han	30	50	20	1	0.97	0.04	1	33 (30-38)	50	0.001 – Tujia(0.45)Dai(0.12)Yi(0.08)	0.003 – Balochi(0.46)Makrani(0.09)Cypriot(0.06)
Brahui/Han	150	5	20	NA	0.2	0.13	0.99	22 (4-262)	47	0.003 – Balochi(0.68)Makrani(0.1)	0.009 – Iranian(0.36)Pathan(0.19)Sindhi(0.1)
Brahui/Han	150	20	20	1	0.48	0.06	1	123 (89-160)	32	0.001 – Tujia(0.48)Dai(0.07)HanNchina(0.07)	0.003 – Balochi(0.46)Sindhi(0.08)Makrani(0.08)
Brahui/Han	150	50	20	1	0.46	0.03	1	161 (113-215)	48	0.001 – Tujia(0.68)Dai(0.09)	0.003 – Balochi(0.38)Makrani(0.09)Sindhi(0.09)
French/Brahui	7	5	20	1	0.65	0.04	0.99	11 (7-18)	7	0.003 – Balochi(0.69)Georgian(0.15)Tunisian(0.06)	0.002 – English(0.31)Scottish(0.24)NorthItalian(0.11)
French/Brahui	7	20	20	1	0.91	0.04	1	8 (6-10)	19	0.003 – Balochi(0.99)	0.002 – English(0.31)Welsh(0.2)Scottish(0.16)
French/Brahui	7	50	20	1	0.95	0.03	1	7 (5-8)	48	0.002 – English(0.33)Welsh(0.22)Scottish(0.2)	0.003 – Balochi(0.87)Makrani(0.13)
French/Brahui	30	5	20	1	0.56	0.03	1	27 (19-37)	7	0.003 – Balochi(0.78)Georgian(0.09)UAE(0.07)	0.002 – English(0.38)Welsh(0.19)Scottish(0.12)
French/Brahui	30	20	20	1	0.83	0.03	1	31 (26-37)	19	0.003 – Balochi(0.93)Makrani(0.07)	0.002 – English(0.31)Welsh(0.2)Scottish(0.14)
French/Brahui	30	50	20	1	0.91	0.02	1	28 (25-34)	48	0.002 – English(0.29)Scottish(0.22)Welsh(0.17)	0.003 – Balochi(0.9)Makrani(0.1)
French/Brahui†	150	5	20	NA	0.02	0.02	0.62	66 (1-716)	31	0.012 – Balochi(0.25)IndianJew(0.19)Palestinian(0.19)	<0.001 – English(0.27)Polish(0.19)Norwegian(0.18)
French/Brahui†	150	20	20	NA	0.08	0.02	0.94	161 (84-415)	41	0.003 – Balochi(0.56)Makrani(0.24)BantuK(0.09)	0.002 – Norwegian(0.3)Lithuanian(0.29)Basque(0.18)
French/Brahui†	150	50	20	1	0.1	0.01	1	77 (40-206)	49	0.002 – Scottish(0.27)English(0.15)Welsh(0.12)	0.003 – Balochi(0.71)Makrani(0.12)NorthItalian(0.07)

Continued on next page

Table S1 – continued from previous page

sim groups	gen	true %	n	c	R_1	R_2^*	FQ_1	date	%	source 1	source 2
French/Brahui	150	50	50	1	0.36	0.04	1	108 (48-152)	48	0.002 – English(0.18)Scottish(0.15)WestSicilian(0.12)	0.003 – Balochi(0.64)NorthItalian(0.14)Welsh(0.1)
French/Brahui	150	50	100	1	0.6	0.14	1	146 (112-191)	44	0.003 – Balochi(0.83)Makrani(0.11)	0.002 – Welsh(0.18)English(0.15)Scottish(0.13)

Table S1: Admixture inference for simulations in [1] (summarised in Figure 2 of main text). Columns give: **sim groups**: the admixing populations (Colomb=Colombian), **gen**: the true date of admixture (generations ago), **true %**: the true proportion of admixture from the second group, **n**: sample size, **c**: fastGLOBETROTTER's conclusion (1 = one admixture date, NA = no admixture), R_1: r-squared fit of a single date (i.e. measuring fit of green line to black lines in Figure S7); R_2^*: additional r-squared explained by adding a second date, FQ_1: fit quality of a single date between only two sources, with low values (< 0.975) indicating $>$2 admixing sources (see [1] for details), **date**: inferred date and 95% CI, %: proportion of admixture from source 1, **source 1:** lowest F_{ST} [2] between either admixing sim group and the single reference population (not shown) inferred to best match the minority contributing source, followed by top three reference populations used in inferred genetic make-up of the minority contributing source (parentheses give the mixture proportions from these references used to describe this source; these values sum to 1 across all reference populations; BantuK = BantuKenya, BantuSA = BantuSouthAfrica), **source2:** same for majority contributing source. † refers to cases where the date was inferred considering only pairs of DNA segments 1-5cM apart; otherwise pairs of DNA segments 1-30cM apart were analysed (see Methods).

		NULL					non-NULL				
**gen**	**method**	**c**	**date**	**%**	**source1**	**source2**	**c**	**date**	**%**	**source1**	**source2**
**45**	GT	1	47 (45-52)	45	Pop10	Pop2	2	30 (29-32)	48	Pop10	Pop2
	fastGT	1	47 (43-50)	45	Pop10	Pop2	1	31 (28-34)	48	Pop10	Pop2
**20**	GT	1	20 (19-21)	39	Pop10	Pop2	1	17 (16-18)	44	Pop10	Pop2
	fastGT	1	20 (18-21)	39	Pop10	Pop2	1	18 (15-19)	44	Pop10	Pop2
**10**	GT	1	10 (10-12)	40	Pop10	Pop2	1	10 (9-11)	41	Pop10	Pop2
	fastGT	1	10 (9-12)	40	Pop10	Pop2	1	10 (9-11)	41	Pop10	Pop2

Table S2: GLOBETROTTER (GT) and fastGLOBETROTTER (fastGT) inference for simulations from [1] that mimic admixture among African ("Pop2") and East Asian ("Pop8") sources, contributing 60% and 40% of ancestry, respectively, followed by a severe bottleneck in the admixed population. See Figure S1 for the demographic history related the admixing populations. **NULL** gives results when using `null.ind=1`, which adjusts date inference for the effects of a post-admixture bottleneck in the target population. **non-NULL** gives results when using `null.ind=0`, which makes no adjustment. Columns give: **gen:** the simulated date of admixture, **c**: GLOBETROTTER/fastGLOBETROTTER's conclusion (1=one admixture date, 2=multiple admixture dates), **date**: inferred date and 95% CI (in generations ago), **%**: proportion of admixture from source 1, **source1:** inferred single surrogate group that best genetically represents the minority contributing source, **source2:** inferred single surrogate group that best genetically represents the majority contributing source.

pop	cluster make-up	pop	cluster make-up
C1	Spa(24) Fra(10) Ger(3) Bel(1) Nor(1)	C2	Spa(88) Fra(5) Bel(2) Ger(1)
C3	Spa(28) Fra(17) Bel(1)	C4	Spa(54) Fra(21) Ita(1) Swe(1)
C5	Spa(5) Fra(4)	C6	Ita(23) Bel(1)
C7	Ita(84) Fra(6) Ger(4) Spa(1) Bel(1)	C8	Ita(45) Ger(2) Bel(1)
C9	Ita(103) Bel(3) Ger(2) Fra(1) Nor(1)	C10	Swe(10) Fra(4) Bel(3) Ger(3) Den(2) Pol(1)
C11	Swe(13) Ger(9) Ita(1) Fra(1) Bel(1) Den(1) Nor(1)	C12	Ger(15) Swe(8) Ita(7) Fra(3) Bel(2) Den(1) Nor(1)
C13	Swe(8) Ger(3) Spa(2) Bel(2) Den(1) Nor(1) Fin(1)	C14	Ita(14) Fra(1) Bel(1) Den(1) Swe(1)
C15	Ita(30)	C16	Ita(106) Fra(6) Bel(2) Ger(2)
C17	Ita(26)	C18	Ita(96) Fra(2)
C19	Fra(22) Ita(18) Bel(1)	C20	Ita(141) Ger(1)
C21	Ita(46) Fra(1)	C22	Swe(7) Ger(6)
C23	Fin(58) Swe(6)	C24	Fin(30) Swe(4)
C25	Fin(57) Swe(1)	C26	Fin(25) Swe(2)
C27	Fin(45)	C28	Fin(32)
C29	Fin(83) Swe(6)	C30	Fin(59) Swe(3)
C31	Fin(56) Swe(7)	C32	Fin(80) Swe(2)
C33	Fin(30)	C34	Nor(8) Bel(6) Ger(3) Ita(2) Den(2) Swe(2)
C35	Fra(76) Den(1)	C36	Swe(11) Ger(2) Bel(1) Pol(1) Nor(1)
C37	Pol(54) Ger(17) Swe(8) Fra(3) Ita(1) Bel(1)	C38	Ger(128) Den(3) Pol(2) Swe(1)
C39	Ger(136) Swe(5) Fra(3) Bel(1) Nor(1)	C40	Swe(46) Nor(11) Den(8) Ger(4) Fra(2)
C41	Ger(113) Swe(2) Bel(1)	C42	Ger(82) Den(7) Swe(4)
C43	Ger(43)	C44	Ger(52) Den(2) Bel(1)
C45	Ger(92) Bel(2) Den(1) Swe(1)	C46	Ger(111) Bel(5) Fra(2)
C47	Ger(105) Fra(7) Bel(4) Swe(4) Spa(1) Den(1)	C48	Ger(151) Fra(2) Bel(2) Swe(2)
C49	Den(153) Nor(7) Swe(2)	C50	Den(140) Swe(12) Nor(2) Ger(1)
C51	Fra(84) Spa(1) Nor(1) Swe(1)	C52	Fra(191) Bel(7) Ger(5) Swe(2) Spa(1)
C53	Bel(181) Fra(5) Ger(2) Ita(1)	C54	Bel(203) Ger(1)
C55	Bel(107)	C56	Swe(23) Nor(9)
C57	Fin(22) Swe(12)	C58	Swe(15) Fin(2) Den(1)
C59	Swe(44)	C60	Swe(77)
C61	Swe(71) Nor(2)	C62	Swe(28) Nor(1) Fin(1)
C63	Swe(88) Nor(2)	C64	Swe(140)
C65	Swe(211) Nor(1)	C66	Swe(70) Den(4)
C67	Swe(154)	C68	Swe(77) Nor(1)
C69	Nor(25) Swe(2)	C70	Nor(16)
C71	Nor(21)	C72	Nor(76) Swe(4)
C73	Nor(28)	C74	Nor(67)
C75	Nor(37)	C76	Nor(91) Swe(1)
C77	Nor(78) Swe(6) Den(3)	C78	Nor(41) Swe(1)
C79	Nor(26) Swe(1)	C80	Nor(86) Swe(2)
C81	Nor(78) Swe(1)	C82	Nor(53)
C83	Nor(52) Swe(1)	C84	Nor(58)
C85	Nor(32)	C86	Nor(36)

Table S3: Number of individuals from each country in each Europe cluster. Bel=Belgium, Den=Denmark, Fin=Finland, Fra=France, Ger=Germany, Ita=Italy, Nor=Norway, Pol=Poland, Spa=Spain, Swe=Sweden

pop	n	region	pop	n	region
colombian(g)	7	Americas	karitiana(g)	11	Americas
maya(g)	21	Americas	pima(g)	14	Americas
surui(g)	5	Americas	bantukenya(g)	11	Bantu
bantusouthafrica(g)	8	Bantu	luhya(c)	94	Bantu
biakapygmy(g)	21	Central Africa	hadza(e)	3	Central Africa
mbutipygmy(g)	13	Central Africa	sandawe(e)	28	Central Africa
balochi(g)	24	Central South Asia	bengali(h)	1	Central South Asia
bhunjia(h)	1	Central South Asia	brahmin(h)	11	Central South Asia
brahui(g)	25	Central South Asia	burusho(g)	25	Central South Asia
chamar(h)	10	Central South Asia	chenchu(b)	4	Central South Asia
dharkar(h)	8	Central South Asia	dhurwa(h)	1	Central South Asia
dusadh(h)	7	Central South Asia	gond(h)	4	Central South Asia
hakkipikki(b)	3	Central South Asia	indian(a)	1	Central South Asia
indianjew(a)	8	Central South Asia	kalash(g)	23	Central South Asia
kanjar(h)	5	Central South Asia	karnataka(a)	8	Central South Asia
kol(h)	16	Central South Asia	kshatriya(h)	7	Central South Asia
kurmi(h)	1	Central South Asia	kurumba(h)	4	Central South Asia
lambadi(h)	1	Central South Asia	makrani(g)	25	Central South Asia
mawasi(h)	1	Central South Asia	meena(h)	1	Central South Asia
meghawal(h)	1	Central South Asia	muslim(h)	5	Central South Asia
nihali(h)	2	Central South Asia	pathan(g)	22	Central South Asia
piramalaikallar(h)	8	Central South Asia	sakd(a)	4	Central South Asia
sindhi(g)	24	Central South Asia	tamilnadu(h)	2	Central South Asia
tharus(h)	2	Central South Asia	upcaste(b)	5	Central South Asia
velamas(h)	9	Central South Asia	hazara(g)	22	Central South Asia2
kyrgyz(f)	16	Central South Asia2	uygur(g)	10	Central South Asia2
uzbekistani(a)	15	Central South Asia2	maasai(c)	97	East Africa
kumyk(k)	14	East Asia	belorussian(a)	9	East Europe
bulgarian(k)	31	East Europe	chuvash(a)	17	East Europe
croatian(d)	19	East Europe	finnish(d)	2	East Europe
hungarian(a)	19	East Europe	lithuanian(a)	10	East Europe
mordovian(k)	15	East Europe	polish(d)	17	East Europe
romanian(a)	16	East Europe	russian(g)	25	East Europe
ukrainian(k)	20	East Europe	ethiopiana(a)	7	Ethiopian
ethiopianjew(a)	11	Ethiopian	ethiopiano(a)	7	Ethiopian
ethiopiant(a)	5	Ethiopian	moroccan(a,d)	25	North Africa
mozabite(g)	29	North Africa	tunisian(a)	12	North Africa
buryat(i)	15	Northeast Asia	daur(g)	9	Northeast Asia
hezhen(g)	8	Northeast Asia	japanese(g)	28	Northeast Asia
mongolian(g)	19	Northeast Asia	oroqen(g)	9	Northeast Asia
tu(g)	10	Northeast Asia	xibo(g)	9	Northeast Asia
yakut(g)	25	Northeast Asia	basque(g)	24	Northwest Europe
english(d)	8	Northwest Europe	french(g)	28	Northwest Europe
german(d)	30	Northwest Europe	germanyaustria(d)	4	Northwest Europe
irish(d)	7	Northwest Europe	norwegian(d)	18	Northwest Europe
orcadian(g)	15	Northwest Europe	scottish(d)	6	Northwest Europe
spanish(a,d)	34	Northwest Europe	welsh(d)	4	Northwest Europe
MS:NIreland(j)	61	Northwest Europe	MS:UK(j)	1854	Northwest Europe
melanesian(g)	10	Oceania	papuan(g)	17	Oceania
sankhomani(e)	30	San	sannamibia(g)	5	San
altai(i)	13	Siberia	burya(i)	2	Siberia
chukchi(i)	5	Siberia	dolgan(i)	7	Siberia
evenk(i)	12	Siberia	ket(i)	2	Siberia
koryake(i)	5	Siberia	nganassan(i)	10	Siberia
selkup(i)	10	Siberia	tuva(i)	13	Siberia
yukagir(i)	4	Siberia	greek(d)	16	South Europe

Continued on next page

Table S4 – continued from previous page					
**pop**	n	**region**	**pop**	n	**region**
northitalian(g)	12	South Europe	sardinian(g)	28	South Europe
siciliane(d)	10	South Europe	southitalian(d)	18	South Europe
tsi(c)	98	South Europe	tuscan(g)	8	South Europe
westsicilian(d)	10	South Europe	bedouin(g)	45	South Middle East
egyptian(a)	12	South Middle East	jordanian(a)	20	South Middle East
lebanese(a)	5	South Middle East	palestinian(g)	46	South Middle East
saudi(a)	19	South Middle East	syrian(a)	16	South Middle East
uae(d)	14	South Middle East	yemeni(a)	9	South Middle East
cambodian(g)	10	Southeast Asia	dai(g)	10	Southeast Asia
han(g)	34	Southeast Asia	hannchina(g)	10	Southeast Asia
lahu(g)	8	Southeast Asia	malayan(a)	1	Southeast Asia
miao(g)	10	Southeast Asia	myanmar(a)	3	Southeast Asia
naga(h)	4	Southeast Asia	naxi(g)	8	Southeast Asia
she(g)	10	Southeast Asia	tujia(g)	10	Southeast Asia
yi(g)	10	Southeast Asia	mandenka(g)	22	West Africa
yoruba(g)	21	West Africa	abhkasian(k)	20	West Asia
adygei(g)	17	West Asia	armenian(a)	35	West Asia
balkar(k)	19	West Asia	chechen(k)	20	West Asia
cypriot(a)	12	West Asia	druze(g)	42	West Asia
georgian(a)	20	West Asia	iranian(a)	20	West Asia
kurd(k)	6	West Asia	lezgin(a)	18	West Asia
nogay(k)	16	West Asia	northossetian(k)	15	West Asia
tajik(k)	15	West Asia	turkish(a)	19	West Asia
turkishe(d)	23	West Asia	turkishn(d)	20	West Asia
turkishs(d)	20	West Asia	turkmen(k)	10	West Asia

Table S4: Surrogate groups used as proxies to ancestral sources in each European cluster, along with (approximate) regional assignments designated for this paper (n=sample size). The published source of each population is given in parentheses, with a=[3], b=[4], c=[5], d=[1], e=[6], f=[7], g=[8], h=[9], i=[10], j=[11], k=[12].

pop	n	cluster make-up	c	AF/EA	R_1	R_2^*	FQ_1	date	%	source 1	source 2
C1	39	Spa(24)Fra(10)Ger(3)	1	9.9/0	0.96	0.32	0.98	1092 (924-1288)	0.08	moroccan(0.34)yoruba(0.29)tsi(0.18)	welsh(0.32)spanish(0.24)MS:NIreland(0.11)
C2	96	Spa(88)Fra(5)Bel(2)	M	12.1/0	0.98	0.26	0.88	868 (756-1008)	0.18<br>0.15	lithuanian(0.55)mandenka(0.15)yoruba(0.07)<br>basque(0.67)welsh(0.24)	welsh(0.42)basque(0.16)tsi(0.15)<br>spanish(0.49)french(0.26)scottish(0.06)
C3	46	Spa(28)Fra(17)Bel(1)	M	9.5/0	0.91	0.24	0.9	616 (448-812)	0.34<br>0.17	basque(0.56)french(0.29)welsh(0.15)<br>moroccan(0.4)basque(0.23)tsi(0.11)	english(0.29)welsh(0.12)tsi(0.11)<br>french(0.58)basque(0.17)welsh(0.12)
C4	77	Spa(54)Fra(21)Ita(1)	2	6.4/1	0.97	0.35	0.88	1344 (1232-1456)<br>364B (784B-308)	0.21<br>0.07	tsi(0.38)basque(0.23)welsh(0.19)<br>tsi(0.34)mozabite(0.14)mandenka(0.12)	english(0.27)spanish(0.26)scottish(0.2)<br>french(0.43)spanish(0.2)welsh(0.15)
C6	24	Ita(23)Bel(1)	M	8.5/0	0.89	0.08	0.83	1008 (784-1344)	0.28<br>0.39	welsh(0.3)southitalian(0.27)tsi(0.12)<br>sardinian(0.78)welsh(0.08)	sardinian(0.61)MS:NIreland(0.12)welsh(0.1)<br>sardinian(0.19)welsh(0.18)tsi(0.17)
C7	96	Ita(84)Fra(6)Ger(4)	1	35.5/3.1	0.99	0.27	0.98	980 (840-1064)	0.19	georgian(0.36)welsh(0.2)mandenka(0.19)	tsi(0.2)welsh(0.17)lezgin(0.16)
C8	48	Ita(45)Ger(2)Bel(1)	U	28.1/0	0.88	0.26	0.89	812 (476-1148)	0.23	armenian(0.46)tsi(0.19)mandenka(0.1)	welsh(0.26)croatian(0.26)tsi(0.1)
C9	110	Ita(103)Bel(3)Ger(2)	M	31.7/0	0.97	0.25	0.92	672 (504-840)	0.23<br>0.39	armenian(0.43)tsi(0.15)mandenka(0.11)<br>welsh(0.32)tsi(0.16)spanish(0.09)	welsh(0.23)tsi(0.12)norwegian(0.11)<br>greek(0.16)southitalian(0.13)tsi(0.12)
C10	23	Swe(10)Fra(4)Bel(3)	1	17.8/0.3	0.77	0.31	0.98	700 (280-1036)	0.26	tuscan(0.19)moroccan(0.18)cypriot(0.14)	german(0.53)welsh(0.23)polish(0.09)
C11	27	Swe(13)Ger(9)Ita(1)	M	9/0	0.71	0.05	0.96	448 (140-756)	0.43<br>0.26	welsh(0.28)tsi(0.23)georgian(0.14)<br>welsh(0.58)MS:NIreland(0.15)tsi(0.12)	belorussian(0.69)lithuanian(0.31)<br>croatian(0.75)
C12	37	Ger(15)Swe(8)Ita(7)	M	1.3/2.2	0.62	0.08	0.97	1064 (616-1176)	0.43<br>0.36	MS:UK(0.58)norwegian(0.12)welsh(0.1)<br>welsh(0.32)syrian(0.12)norwegian(0.08)	ukrainian(0.39)croatian(0.31)romanian(0.12)<br>polish(0.37)lithuanian(0.33)welsh(0.27)
C13	18	Swe(8)Ger(3)Spa(2)	1	10.1/5.3	0.78	0.08	0.98	1064 (868-1316)	0.26	greek(0.58)daur(0.24)welsh(0.09)	croatian(0.25)norwegian(0.16)MS:UK(0.14)
C14	18	Ita(14)Fra(1)Bel(1)	M	28.2/0	0.52	0.05	0.82	532 (196-1344)	0.32<br>0.42	sardinian(0.62)tsi(0.22)welsh(0.08)<br>tsi(0.21)sardinian(0.17)armenian(0.1)	welsh(0.22)armenian(0.1)german(0.1)<br>welsh(0.4)sardinian(0.2)german(0.14)
C16	116	Ita(106)Fra(6)Bel(2)	M	24.2/0	0.97	0.18	0.91	504 (336-672)	0.23<br>0.45	armenian(0.47)welsh(0.15)tsi(0.1)<br>welsh(0.42)tsi(0.16)german(0.1)	welsh(0.24)MS:NIreland(0.15)lithuanian(0.11)<br>northitalian(0.23)tsi(0.17)kurd(0.15)
C17	26	Ita(26)	1	8.8/0.2	0.68	0.06	0.98	560 (28-784)	0.45	northitalian(0.47)cypriot(0.1)armenian(0.06)	welsh(0.56)tsi(0.18)MS:NIreland(0.14)
C18	98	Ita(96)Fra(2)	1	11.9/0	0.89	0.06	0.98	476 (336-672)	0.32	tsi(0.17)northitalian(0.14)tunisian(0.12)	welsh(0.28)german(0.18)tsi(0.15)
C19	41	Fra(22)Ita(18)Bel(1)	1	12.8/0	0.84	0.04	0.98	420 (168-672)	0.24	greek(0.2)moroccan(0.15)tsi(0.15)	welsh(0.27)english(0.27)german(0.15)
C20	142	Ita(141)Ger(1)	M	11.7/0	0.94	0.1	0.97	420 (252-560)	0.35<br>0.26	tsi(0.21)southitalian(0.16)turkishe(0.14)<br>MS:UK(0.53)tsi(0.19)german(0.15)	welsh(0.32)scottish(0.15)tsi(0.15)<br>northitalian(0.29)croatian(0.17)welsh(0.16)
C21†	47	Ita(46)Fra(1)	M	9.4/0	0.84	0.29	0.97	756 (224B-1092)	0.37<br>0.32	tuscan(0.36)armenian(0.22)greek(0.09)<br>welsh(0.49)MS:NIreland(0.18)cypriot(0.11)	welsh(0.43)MS:NIreland(0.14)german(0.14)<br>welsh(0.35)tsi(0.18)lithuanian(0.15)
C24†	34	Fin(30)Swe(4)	1	0/12.7	0.96	0.17	1	896B (1372-336B)	0.25	russian(0.4)daur(0.25)hezhen(0.11)	welsh(0.78)russian(0.16)
C25†	58	Fin(57)Swe(1)	1	0/15.3	0.98	0.32	1	280B (700B-112)	0.21	oroqen(0.54)russian(0.37)dolgan(0.09)	welsh(0.7)russian(0.2)chuvash(0.06)
C26†	27	Fin(25)Swe(2)	1	0/13.5	0.89	0.05	0.99	728B (1456-112B)	0.23	oroqen(0.43)russian(0.39)chuvash(0.07)	welsh(0.58)norwegian(0.19)russian(0.12)
C27†	45	Fin(45)	1	1.3/11.6	0.97	0.24	0.99	448B (980-56B)	0.26	russian(0.47)oroqen(0.34)nganassan(0.1)	welsh(0.82)russian(0.14)
C28†	32	Fin(32)	1	0.9/11.3	0.96	0.29	1	308B (1036B-140)	0.27	russian(0.46)oroqen(0.39)dolgan(0.07)	welsh(0.81)russian(0.14)
C29	89	Fin(83)Swe(6)	M	0/7.9	0.92	0.27	0.97	756 (448-952)	0.32<br>0.23	russian(0.6)oroqen(0.21)nganassan(0.07)<br>welsh(0.73)norwegian(0.1)oroqen(0.1)	welsh(0.4)norwegian(0.33)russian(0.17)<br>finnish(0.56)russian(0.37)
C30†	62	Fin(59)Swe(3)	1	0/17.5	0.98	0.3	1	840B (1176-420B)	0.22	oroqen(0.54)russian(0.3)nganassan(0.15)	welsh(0.72)russian(0.19)
C31†	63	Fin(56)Swe(7)	1	0/10.9	0.98	0.19	0.99	364B (672B-56)	0.32	russian(0.48)oroqen(0.17)dolgan(0.12)	welsh(0.61)russian(0.24)orcadian(0.13)
C32†	82	Fin(80)Swe(2)	1	0/10.9	0.98	0.21	0.99	616B (1092-392B)	0.34	russian(0.37)oroqen(0.31)dolgan(0.12)	welsh(0.65)russian(0.22)orcadian(0.13)
C33†	30	Fin(30)	1	0/12.1	0.94	0.27	1	644B (1540-280B)	0.32	russian(0.41)oroqen(0.29)evenk(0.09)	welsh(0.48)norwegian(0.24)russian(0.15)
C34	23	Nor(8)Bel(6)Ger(3)	1	2.7/0.3	0.71	0.09	0.99	1260 (868-1764)	0.04	sardinian(0.48)orcadian(0.34)yoruba(0.07)	welsh(0.6)MS:NIreland(0.2)MS:UK(0.13)
C35†	77	Fra(76)Den(1)	1	2.6/0	0.84	0.09	0.99	392 (1344B-924)	0.23	welsh(0.27)tsi(0.24)southitalian(0.24)	welsh(0.5)irish(0.28)norwegian(0.07)
C36	16	Swe(11)Ger(2)Bel(1)	M	2.6/9.2	0.87	0.13	0.93	812 (532-1120)	0.16<br>0.28	lithuanian(0.56)miao(0.16)she(0.16)<br>welsh(0.82)	lithuanian(0.27)polish(0.25)welsh(0.21)<br>lithuanian(0.64)russian(0.12)koryake(0.06)
C37	84	Pol(54)Ger(17)Swe(8)	M	1.2/4.6	0.77	0.04	0.86	784 (560-924)	0.25	welsh(0.91)	lithuanian(0.59)belorussian(0.19)mordovian(0.11)

Continued on next page

Table S5 – continued from previous page

pop	n	cluster make-up	c	AF/EA	R_1	R_2^*	FQ_1	date	%	source 1	source 2
									0.15	welsh(0.56)russian(0.16)buryat(0.13)	polish(0.74)lithuanian(0.18)
C38	134	Ger(128)Den(3)Pol(2)	1	0/0	0.9	0.25	1	1232 (1148-1344)	0.43	welsh(0.42)MS:UK(0.42)tsi(0.1)	belorussian(0.79)polish(0.2)
C39	146	Ger(136)Swe(5)Fra(3)	1	0/0	0.9	0.12	1	1148 (1036-1260)	0.49	belorussian(0.69)polish(0.14)ukrainian(0.11)	MS:UK(0.36)english(0.22)welsh(0.2)
C40	71	Swe(46)Nor(11)Den(8)	1	0.4/0.7	0.79	0.1	0.99	1064 (896-1232)	0.3	belorussian(0.34)ukrainian(0.14)norwegian(0.13)	MS:UK(0.5)welsh(0.29)german(0.11)
C41	116	Ger(113)Swe(2)Bel(1)	1	0/0	0.86	0.17	1	1316 (1204-1428)	0.38	polish(0.64)ukrainian(0.25)lithuanian(0.07)	MS:UK(0.49)welsh(0.26)german(0.2)
C42	93	Ger(82)Den(7)Swe(4)	1	0/0	0.87	0.11	1	1260 (1176-1372)	0.37	polish(0.74)lithuanian(0.07)german(0.06)	MS:UK(0.54)welsh(0.4)german(0.06)
C43	43	Ger(43)	1	9.8/0	0.74	0.04	1	532 (420-812)	0.19	greek(0.2)tsi(0.14)cypriot(0.12)	welsh(0.54)MS:UK(0.21)german(0.13)
C44	55	Ger(52)Den(2)Bel(1)	1	4.6/0	0.74	0.05	1	588 (364-812)	0.13	croatian(0.29)belorussian(0.23)tsi(0.23)	welsh(0.9)norwegian(0.07)
C45	96	Ger(92)Bel(2)Den(1)	1	6.2/0	0.88	0.16	1	784 (532-896)	0.18	greek(0.31)ukrainian(0.13)cypriot(0.12)	welsh(0.64)MS:UK(0.22)norwegian(0.1)
C46	118	Ger(111)Bel(5)Fra(2)	1	8.3/0	0.91	0.08	1	532 (392-672)	0.2	greek(0.22)tsi(0.12)cypriot(0.12)	MS:UK(0.56)welsh(0.27)german(0.1)
C47	122	Ger(105)Fra(7)Bel(4)	1	10.5/0	0.92	0.11	1	616 (420-700)	0.25	cypriot(0.18)greek(0.16)tsi(0.15)	welsh(0.34)MS:UK(0.34)german(0.19)
C48	157	Ger(151)Fra(2)Bel(2)	1	5.6/0	0.89	0.14	0.99	756 (616-924)	0.31	croatian(0.23)greek(0.22)armenian(0.11)	welsh(0.37)MS:UK(0.27)german(0.23)
C49	162	Den(153)Nor(7)Swe(2)	1	2.9/0	0.89	0.09	1	644 (504-812)	0.15	lithuanian(0.29)tsi(0.23)croatian(0.2)	welsh(0.81)MS:NIreland(0.11)norwegian(0.08)
C50	155	Den(140)Swe(12)Nor(2)	1	0.9/0.3	0.81	0.11	1	896 (812-1092)	0.2	polish(0.5)tsi(0.12)ukrainian(0.11)	welsh(0.82)norwegian(0.12)MS:NIreland(0.06)
C51	87	Fra(84)Spa(1)Nor(1)	U	11.8/0	0.93	0.23	0.82	392 (224-504)	0.14	tsi(0.25)moroccan(0.17)greek(0.09)	french(0.33)welsh(0.26)scottish(0.18)
C52	206	Fra(191)Bel(7)Ger(5)	M	10.8/0	0.97	0.17	0.97	420 (308-560)	0.15	tsi(0.25)greek(0.21)moroccan(0.16)	english(0.38)welsh(0.24)MS:UK(0.11)
									0.44	welsh(0.75)MS:NIreland(0.14)	english(0.34)welsh(0.26)greek(0.12)
C53	189	Bel(181)Fra(5)Ger(2)	1	11.6/0	0.96	0.17	1	532 (420-616)	0.16	tsi(0.19)greek(0.16)cypriot(0.14)	MS:UK(0.67)welsh(0.22)german(0.06)
C54	204	Bel(203)Ger(1)	1	8.5/0	0.95	0.1	1	504 (392-560)	0.16	greek(0.22)armenian(0.22)tsi(0.21)	MS:UK(0.51)welsh(0.34)norwegian(0.06)
C55	107	Bel(107)	1	7.9/0	0.92	0.1	1	560 (364-672)	0.15	greek(0.25)armenian(0.19)tsi(0.18)	MS:UK(0.59)welsh(0.28)norwegian(0.07)
C56	32	Swe(23)Nor(9)	1	0/5.6	0.89	0.18	0.99	784 (616-952)	0.25	russian(0.72)nganassan(0.1)oroqen(0.1)	welsh(0.62)norwegian(0.2)lithuanian(0.11)
C57	34	Fin(22)Swe(12)	1	0/4.9	0.88	0.23	0.99	476 (224-672)	0.32	russian(0.77)dolgan(0.09)oroqen(0.07)	welsh(0.57)norwegian(0.17)lithuanian(0.13)
C58†	18	Swe(15)Fin(2)Den(1)	1	0/9.7	0.85	0.18	0.99	952B (2408-168B)	0.21	russian(0.49)oroqen(0.13)dolgan(0.12)	welsh(0.56)norwegian(0.33)orcadian(0.1)
C59	44	Swe(44)	1	0/3.8	0.88	0.26	1	980 (840-1176)	0.24	russian(0.41)norwegian(0.32)chuvash(0.07)	welsh(0.57)MS:NIreland(0.25)norwegian(0.11)
C60	77	Swe(77)	1	0/3.5	0.91	0.21	0.99	896 (700-1064)	0.19	russian(0.39)norwegian(0.2)lithuanian(0.17)	welsh(0.56)MS:NIreland(0.17)norwegian(0.12)
C61	73	Swe(71)Nor(2)	1	0/5.6	0.91	0.27	1	588 (420-812)	0.19	russian(0.41)norwegian(0.27)koryake(0.11)	welsh(0.62)MS:NIreland(0.17)norwegian(0.12)
C62	30	Swe(28)Nor(1)Fin(1)	1	0/2.4	0.8	0.19	0.99	840 (588-1092)	0.37	finnish(0.65)russian(0.2)	welsh(0.62)MS:NIreland(0.19)norwegian(0.1)
C63	90	Swe(88)Nor(2)	1	0/3.7	0.89	0.14	0.99	672 (560-868)	0.18	russian(0.31)norwegian(0.3)lithuanian(0.18)	welsh(0.65)norwegian(0.15)MS:NIreland(0.14)
C64	140	Swe(140)	2	0/2.9	0.93	0.37	0.99	1288 (1148-1568)	0.14	russian(0.51)lithuanian(0.24)norwegian(0.15)	welsh(0.52)norwegian(0.21)lithuanian(0.06)
								1400B (1876-308B)	0.16	lithuanian(0.28)russian(0.21)norwegian(0.19)	welsh(0.66)MS:NIreland(0.18)russian(0.06)
C65	212	Swe(211)Nor(1)	2	0/0.5	0.94	0.46	0.99	1288 (1120-1624)	0.32	polish(0.26)welsh(0.2)norwegian(0.18)	welsh(0.43)MS:UK(0.27)norwegian(0.17)
								700B (1036B-28)	0.05	koryake(0.48)russian(0.16)nganassan(0.09)	welsh(0.43)MS:UK(0.18)norwegian(0.16)
C66	74	Swe(70)Den(4)	1	0.4/0.3	0.68	0.09	1	784 (644-1176)	0.18	belorussian(0.34)russian(0.19)ukrainian(0.18)	welsh(0.63)norwegian(0.16)MS:UK(0.1)
C67	154	Swe(154)	1	0/2.2	0.88	0.17	1	532 (364-812)	0.18	ukrainian(0.71)russian(0.11)norwegian(0.07)	welsh(0.71)MS:NIreland(0.13)norwegian(0.11)
C68	78	Swe(77)Nor(1)	1	0/1.6	0.69	0.08	0.99	672 (280-896)	0.22	norwegian(0.43)lithuanian(0.28)russian(0.07)	welsh(0.7)norwegian(0.16)MS:NIreland(0.15)
C69	27	Nor(25)Swe(2)	1	0.3/3.8	0.83	0.31	1	1204 (1120-1456)	0.16	russian(0.57)chuvash(0.14)nganassan(0.07)	welsh(0.43)norwegian(0.39)MS:NIreland(0.09)
C70	16	Nor(16)	1	0/5.3	0.73	0.11	0.99	756 (280-1064)	0.22	norwegian(0.37)russian(0.34)oroqen(0.06)	welsh(0.66)MS:NIreland(0.22)
C71	21	Nor(21)	1	0/5	0.79	0.12	1	952 (504-1092)	0.19	norwegian(0.56)russian(0.12)nganassan(0.1)	welsh(0.61)MS:NIreland(0.23)norwegian(0.12)
C72	80	Nor(76)Swe(4)	1	0.2/6.3	0.92	0.26	1	728 (644-952)	0.12	norwegian(0.37)russian(0.27)nganassan(0.12)	welsh(0.57)norwegian(0.19)MS:NIreland(0.18)
C73	28	Nor(28)	1	0/4.9	0.87	0.17	1	868 (588-1092)	0.17	norwegian(0.49)russian(0.22)nganassan(0.1)	welsh(0.55)MS:NIreland(0.28)norwegian(0.14)
C74	67	Nor(67)	1	0/3.8	0.91	0.29	1	1092 (952-1260)	0.2	norwegian(0.52)russian(0.2)nganassan(0.1)	welsh(0.6)MS:NIreland(0.23)norwegian(0.12)
C75†	37	Nor(37)	1	0.5/2.9	0.86	0.21	0.99	308B (868B-868)	0.07	russian(0.26)nganassan(0.16)oroqen(0.12)	welsh(0.58)norwegian(0.27)MS:NIreland(0.12)
C76	92	Nor(91)Swe(1)	1	0/3.2	0.88	0.26	0.99	672 (448-868)	0.2	norwegian(0.61)russian(0.21)oroqen(0.06)	welsh(0.67)MS:NIreland(0.16)norwegian(0.14)
C77	87	Nor(78)Swe(6)Den(3)	1	0/3.5	0.85	0.08	0.98	560 (336-784)	0.18	norwegian(0.59)russian(0.15)dolgan(0.07)	welsh(0.65)MS:NIreland(0.19)norwegian(0.12)
C78	42	Nor(41)Swe(1)	1	0/3	0.66	0.02	0.99	532 (280-1008)	0.14	norwegian(0.49)russian(0.18)selkup(0.14)	welsh(0.53)norwegian(0.44)
C80	88	Nor(86)Swe(2)	M	0/5.6	0.89	0.29	0.95	448 (140-672)	0.36	norwegian(0.84)nganassan(0.06)	welsh(0.94)orcadian(0.06)

Continued on next page

Table S5 – continued from previous page

pop	n	cluster make-up	c	AF/EA	R_1	R_2^*	FQ_1	date	%	source 1	source 2
									0.45	lithuanian(0.9)norwegian(0.1)	welsh(0.82)surui(0.12)
C81	79	Nor(78)Swe(1)	1	0/3.4	0.87	0.09	0.99	532 (308-728)	0.18	norwegian(0.66)russian(0.14)nganassan(0.08)	welsh(0.54)MS:NIreland(0.34)norwegian(0.1)
C82	53	Nor(53)	M	0.7/2.5	0.62	0.13	0.9	504 (196B-840)	0.24	norwegian(0.84)selkup(0.06)	MS:NIreland(0.52)welsh(0.45)
									0.3	welsh(0.42)lithuanian(0.41)norwegian(0.09)	welsh(0.95)
C83†	53	Nor(52)Swe(1)	M	0/4.2	0.8	0.12	0.97	448B (1288B-420)	0.06	melanesian(0.23)kurumba(0.15)selkup(0.14)	welsh(0.63)norwegian(0.28)MS:NIreland(0.08)
									0.41	norwegian(0.65)lithuanian(0.16)MS:NIreland(0.14)	welsh(0.86)orcadian(0.1)
C84†	58	Nor(58)	1	0/4.4	0.87	0.17	1	644B (1568B-140)	0.14	welsh(0.6)dolgan(0.13)nganassan(0.11)	welsh(0.74)norwegian(0.15)
C85	32	Nor(32)	1	0/2.8	0.63	0.02	0.99	392 (56B-952)	0.17	norwegian(0.84)yakut(0.09)	welsh(0.84)orcadian(0.06)norwegian(0.05)
C86	36	Nor(36)	M	0/3.1	0.68	0.06	0.97	392 (28-756)	0.31	norwegian(0.81)russian(0.06)oroqen(0.06)	welsh(0.95)orcadian(0.05)
									0.42	welsh(0.79)surui(0.09)russian(0.06)	norwegian(1)

Table S5: Admixture inference for each European cluster. Columns give: **n**: sample size, **cluster make-up**: the three countries most represented in the cluster (counts in parentheses; see Table S3 for full list), **c**: fastGLOBETROTTER's conclusion (1=one admixture date, 2=multiple admixture dates, M=one-date with $>$2 sources, U=admixture difficult to classify, N=caution should be used due to low signal with $R_1 < 0.5$), **AF/EA**: total proportion of DNA matched to *"African/W.Asia"* and *"E.Asia/Siberia"* groups described in Figure 3; R_1: r-squared fit of a single date (i.e. measuring fit of green line to black lines in Figure S7); R_2^*: additional r-squared explained by adding a second date, FQ_1: fit quality of a single date between only two sources, with low values (< 0.975) indicating $>$2 admixing sources (see [1] for details), **date**: inferred date and 95% CI (B=BCE, otherwise CE), %: proportion of admixture from source 1, **source 1:** top three reference populations used in inferred genetic make-up of the minority contributing source (parentheses give the mixture proportions from these references used to describe this source; these values sum to 1 across all reference populations), **source2:** same for majority contributing source.

Supplementary Figures

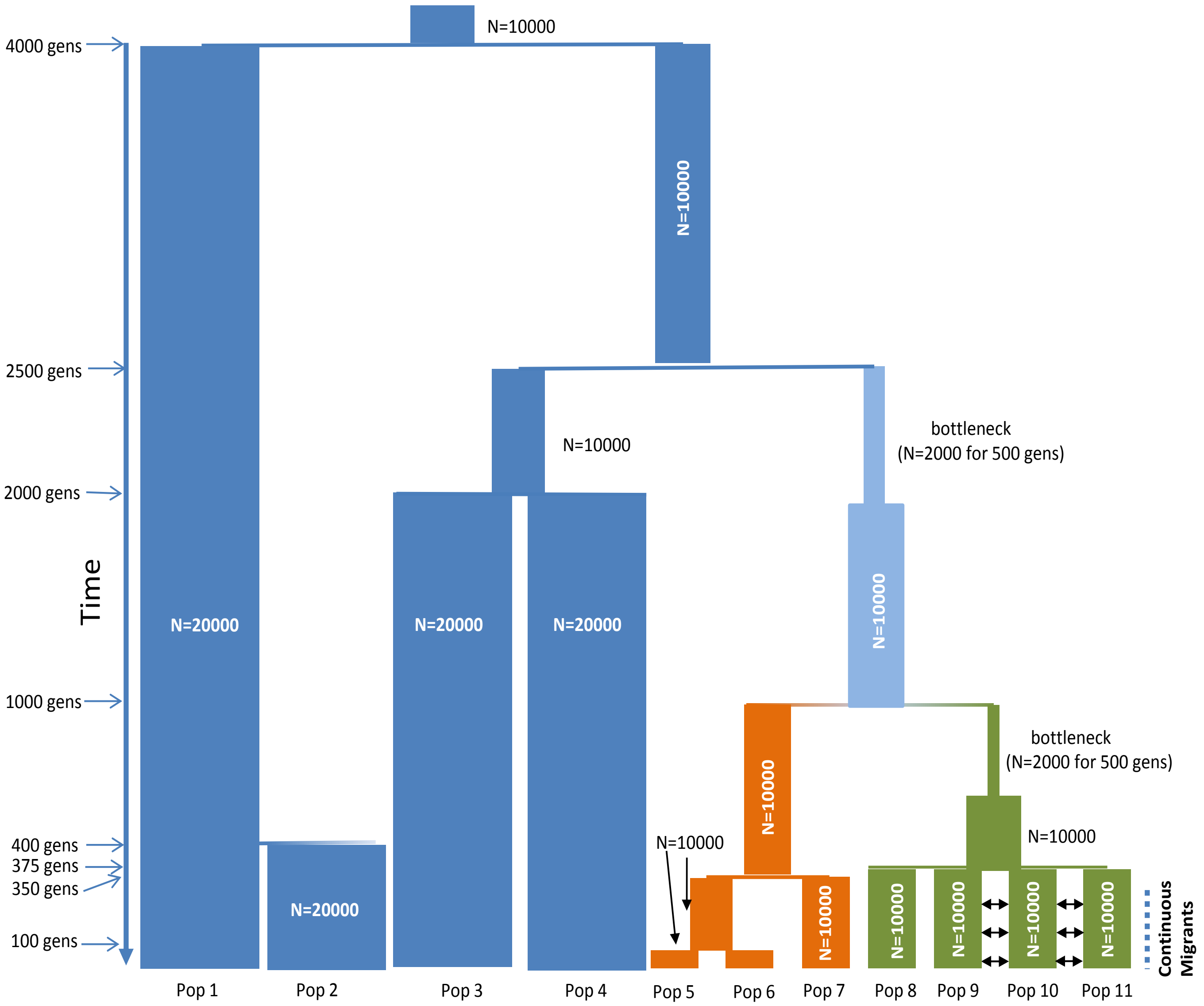

Figure S1: Simulated history for populations 1-11, generated using the coalescent-based software MaCS [13], taken from [1]. Roughly speaking, populations 1-4 in blue are meant to represent diversity in African groups, with populations 5-7 in orange and 8-11 in green representing Western Eurasian and East Asian groups, respectively. 100 generations (gens) denotes the split between Pop5 and Pop6; 350 gens the split between Pop7 and Pop5/Pop6; 375 gens the simultaneous split of populations 8-11; 400 gens the split of Pop1 and Pop2; 1000 gens the split of Pop5/Pop6/Pop7 and Pop8/Pop9/Pop10/Pop11; 2000 gens the split of Pop3 and Pop4; 2500 gens the split of Pop3/Pop4 and Pop5/Pop6/Pop7/Pop8/Pop9/Pop10/Pop11; and 4000 gens the split of Pop1/Pop2 and all other populations.

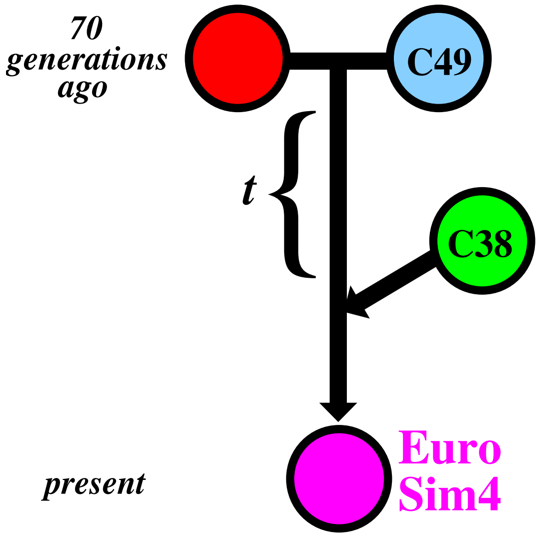

Figure S2: Simulated history for "EuroSim4" (represented by the pink circle) designed to mimic a scenario that might explain patterns observed in our European analysis. Here we simulate admixture occurring 70 generations ago between the Evenk from Northern Asia (red circle) and Europe cluster C49 primarily comprised of Danish (cyan circle), with this admixed group subsequently intermixing with Europe cluster C38 primarily comprised of Germans (green circle) t generations later (here $t = 60$). Given the genetic similarity between Europe clusters C38 and C49, for small t (i.e. $t \leq 60$) fastGLOBETROTTER may infer only single pulse of admixture in "EuroSim4", with inferred date somewhere along the branch indicated by t and a higher proportion of European-like ancestry relative to a scenario where there is no additional pulse of admixture from Europe cluster C38.

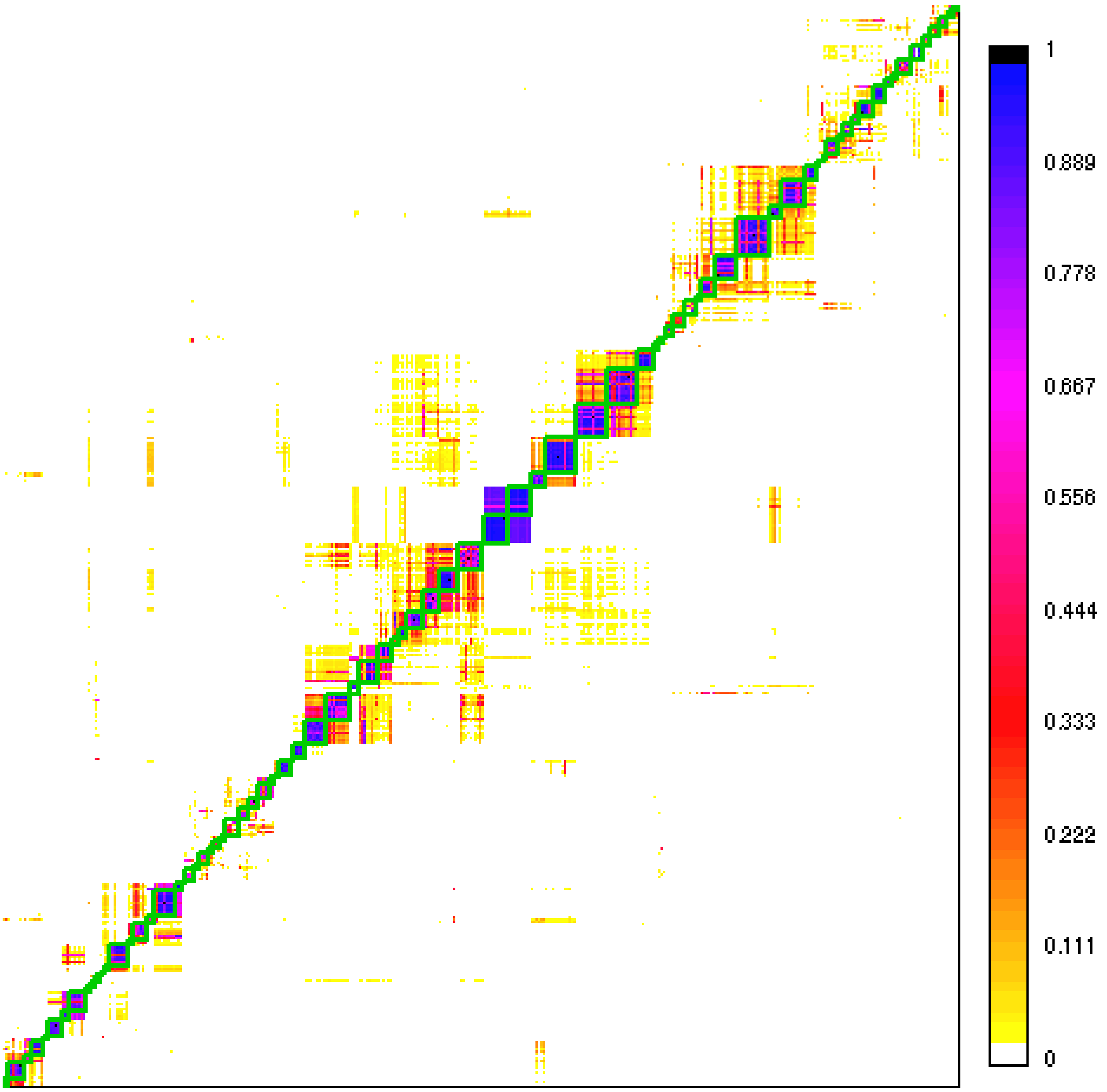

Figure S3: The proportion of fineSTRUCTURE MCMC samples whereby the 6,209 Europeans in the rows/columns were clustered together. Green rectangles denote the 86 clusters we used for our final analyses.

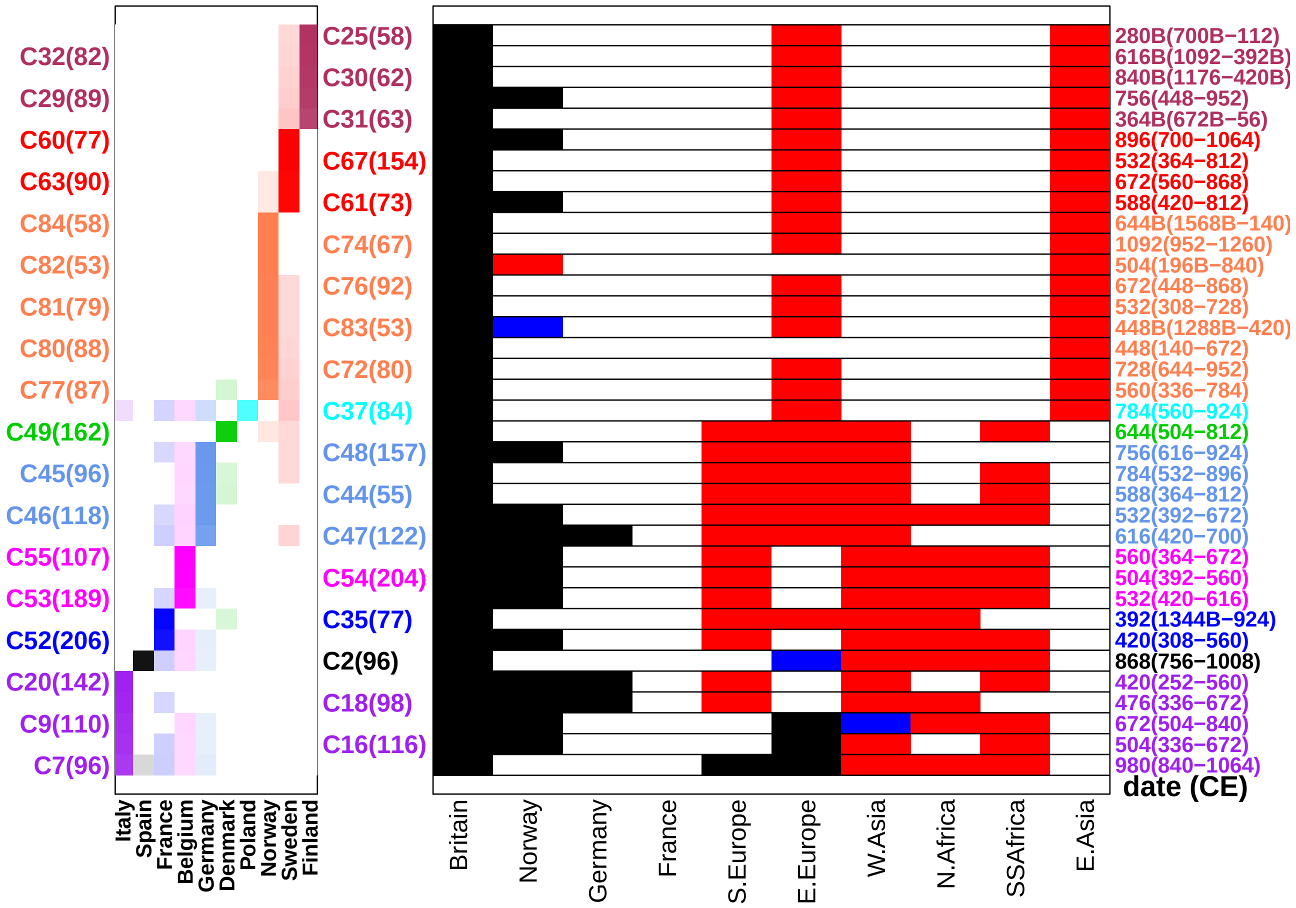

Figure S4: **(right:)** Inferred admixing sources for the 36 Europe clusters that contain >50 individuals, have a single inferred date, and have a >2% inferred contribution from reference groups in E.Asia/Siberia or in Africa/W.Asia (as in main text Figure 3). Black, red and blue colors refer to distinct admixing sources in each row. Geographic regions (columns) were classified into sources by finding the admixture probability curve with the highest r-squared fit assuming one date of admixture (i.e. fit of green line to black lines in Figures S7-S17), among all curves containing a population from Britain and a population from the given column. If this curve was monotonically decreasing, the region was assigned to the black source, otherwise it was assigned to the red source. In clusters where fastGLOBETROTTER inferred >2 sources mixing at the same time, the blue source reflect cases where the fitted line assuming one date (green lines in Figures S7-S17) and the fitted line assuming one date with only two sources (cyan lines in Figures S7-S17) are not both increasing or both decreasing for any given curve. Britain={MS:UK, english, NIreland, orcadian, scottish, welsh}, Germany={germanyaustria, german}, W.Asia={South Middle East, West Asia}, SSAfrica={West Africa, San, Central Africa, East Africa, Bantu, Ethiopian}, E.Asia={Siberia, Northeast Asia, Southeast Asia}. **(left:)** The proportion of individuals in each cluster (row) from each country (column), with clusters' sample sizes in parentheses and countries' colors based on the map in Figure 3 of the main text. Each cluster's label at left and inferred admixture date (+95% CI; B=BCE) at right is colored according to its majority contributing country.

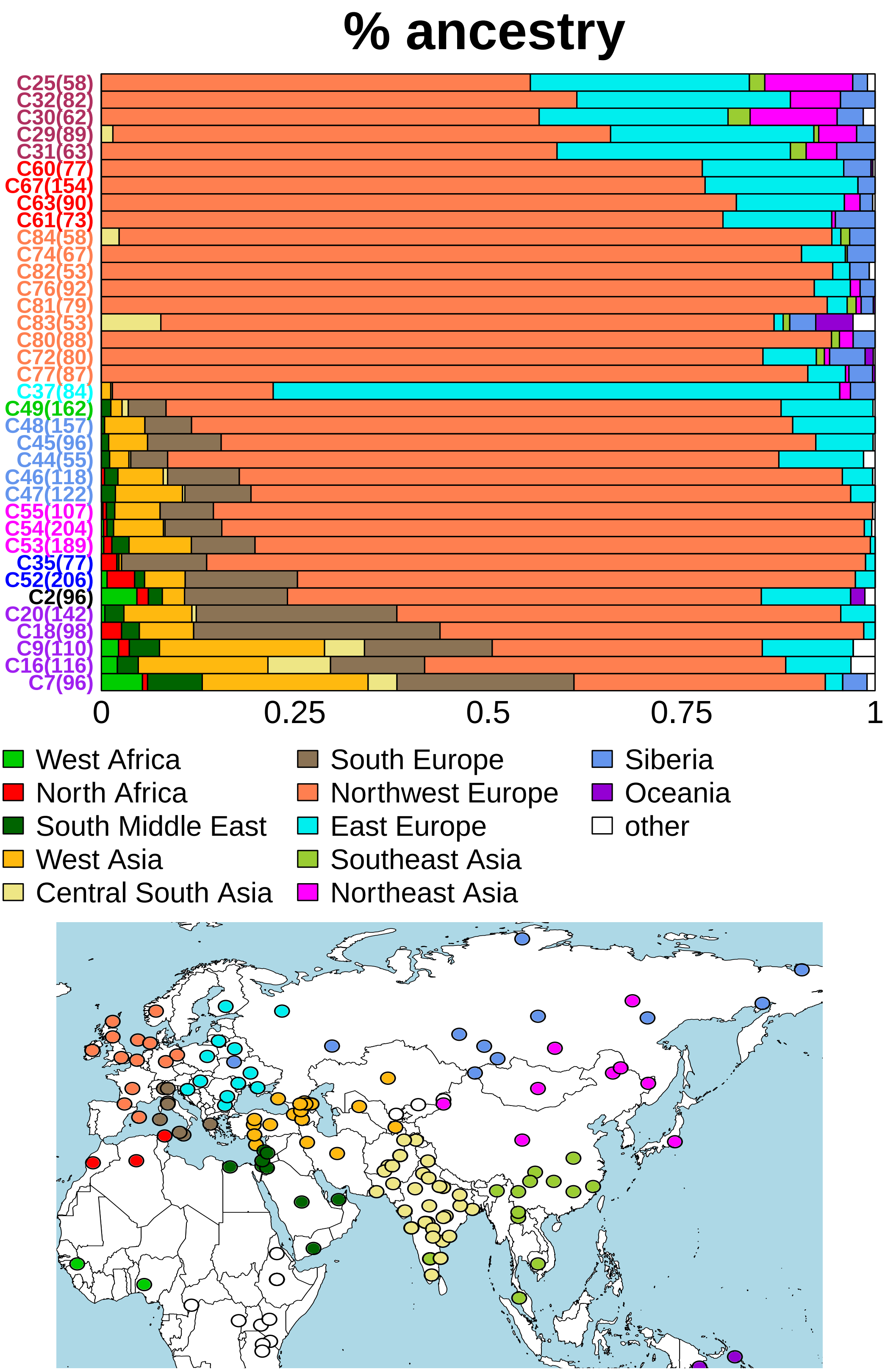

Figure S5: Inferred average proportion of matching to reference populations (bottom map), for all 36 Europe clusters (rows) that contain >50 individuals, have a single inferred date, and have a >2% inferred contribution from reference groups in E.Asia/Siberia or in Africa/W.Asia (as in main text Figure 3). Number of individuals in each cluster are given in parentheses after the cluster label.

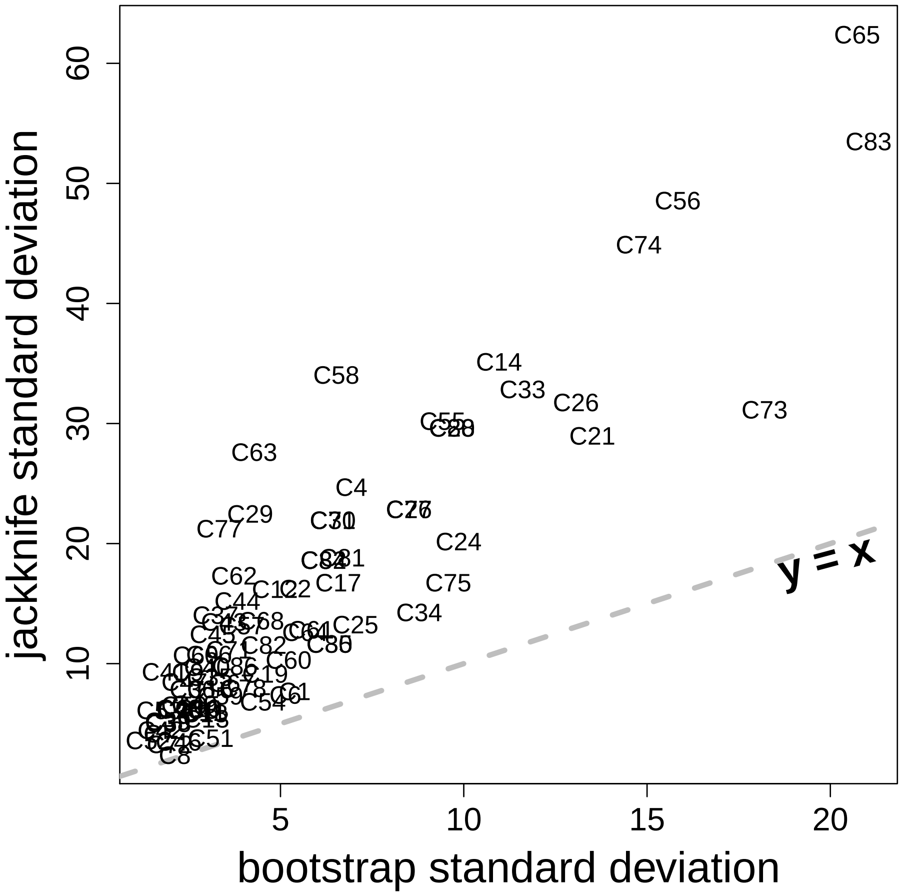

Figure S6: Comparison of standard deviations for date estimates when using jackknifing versus bootstrapping, for all 76 European clusters that inferred “one-date” or “one-date, multiway” admixture. The dotted grey line depicts the line of equality. Jackknife estimates are consistently larger than bootstrap estimates, as expected [14], but they are highly correlated.

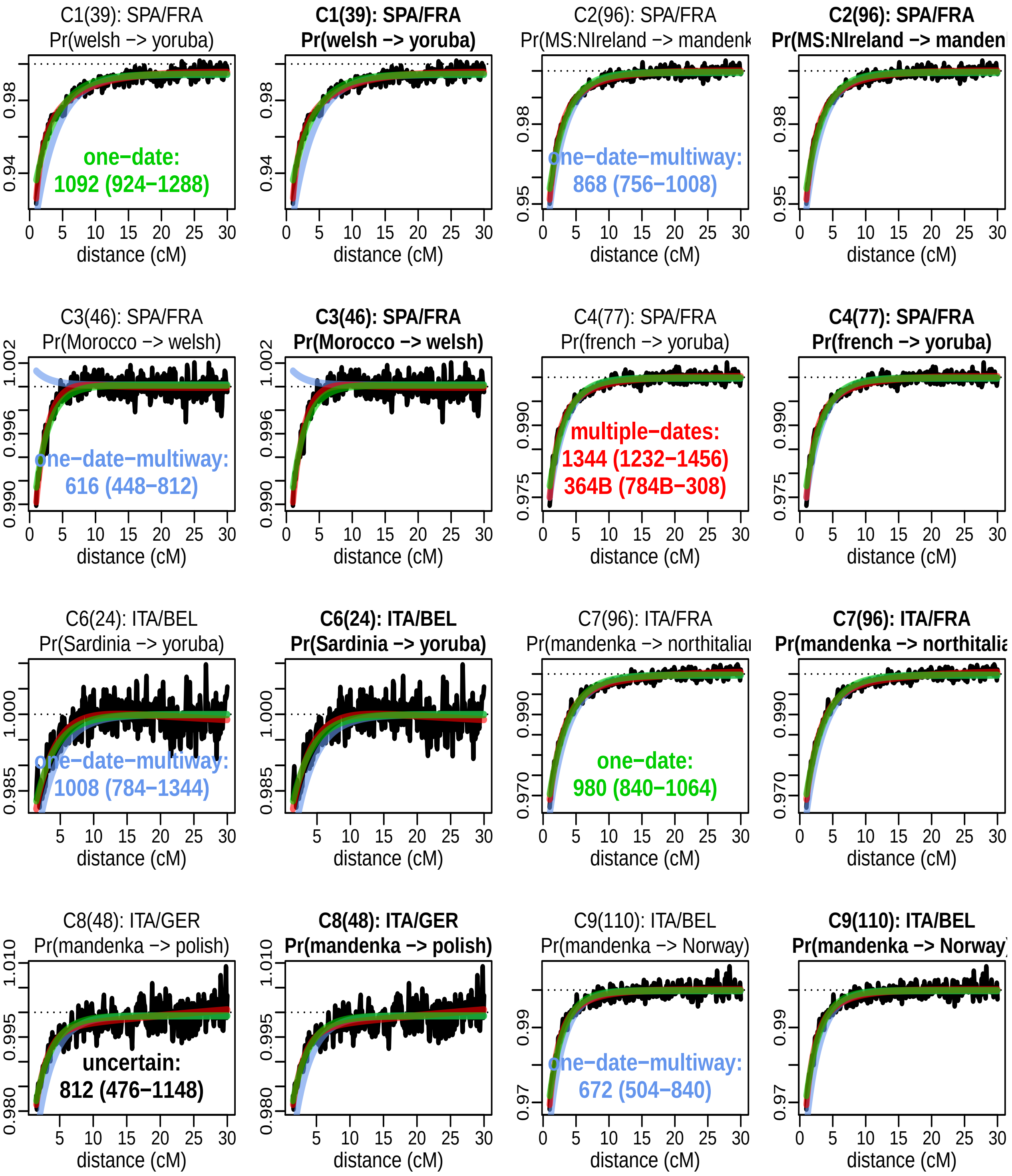

Figure S7: FastGLOBETROTTER's admixture probability curves for Europe clusters C1-C4 and C6-C9. The cluster number, with sample size in parenthesis, and top two countries represented in the cluster are given in the top line. The black lines in each plot show the (scaled) probability, within that cluster's individuals, of matching two DNA segments to the reference populations listed just below the cluster name versus the centimorgan (cM) distance between the two DNA segments. Green lines depict the model fit when assuming a single pulse (date) of admixture, cyan lines when assuming a single pulse of admixture between only two sources, and red lines when assuming two pulses of admixture with distinct dates. There are two plots per cluster. The left plot depicts curves for the monotonically increasing reference pairing with highest r-squared between the green and black lines. The right plot depicts the same among reference pairings containing an African (plot title in bold) or East Asian (plot title in italics) population. In the left plot, we provide the inferred type of admixture and date(s) (B=BCE, otherwise CE) with 95% CI.

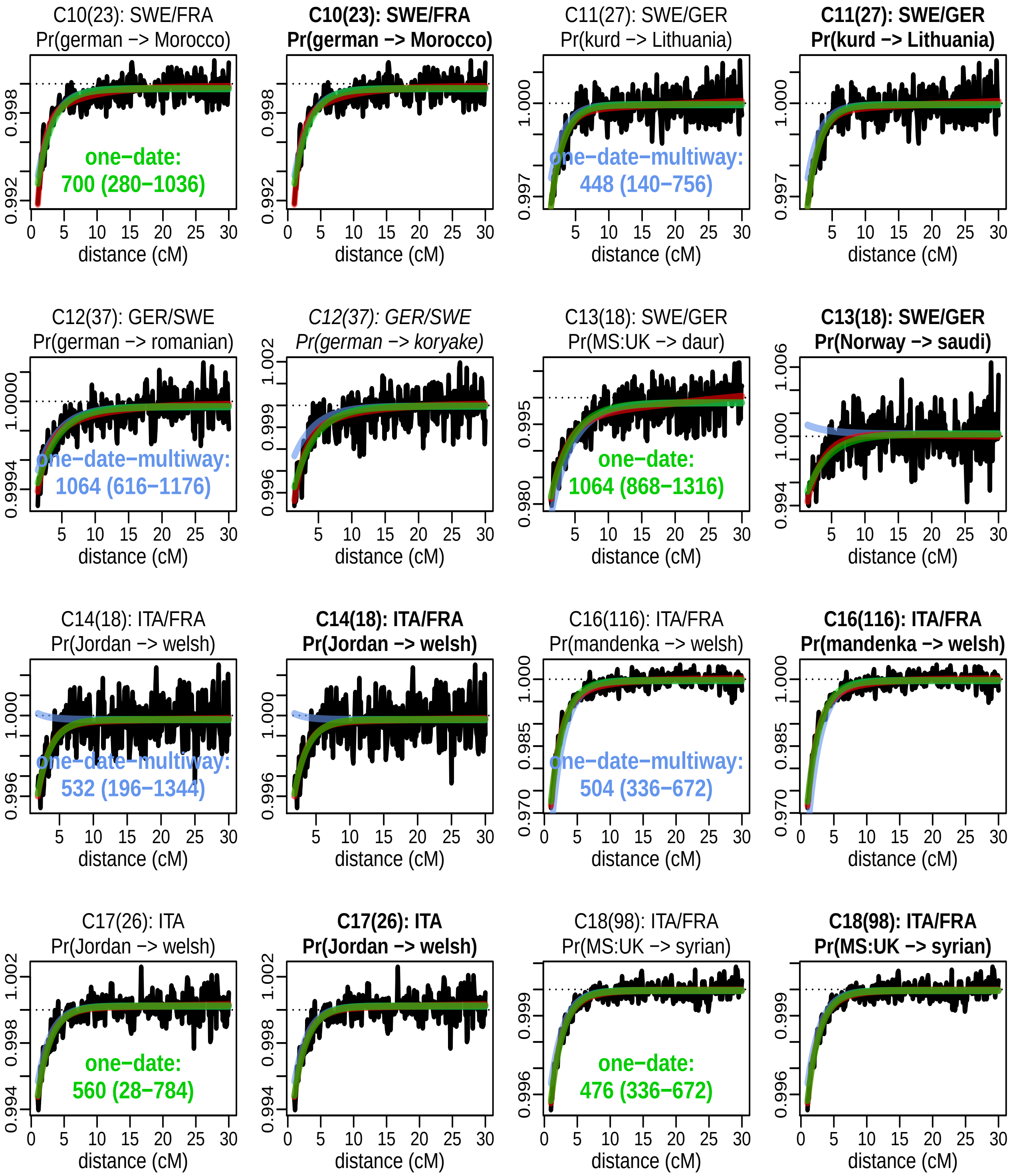

Figure S8: FastGLOBETROTTER's admixture probability curves for Europe clusters C10-C14 and C16-C18. See Figure S7 legend for details.

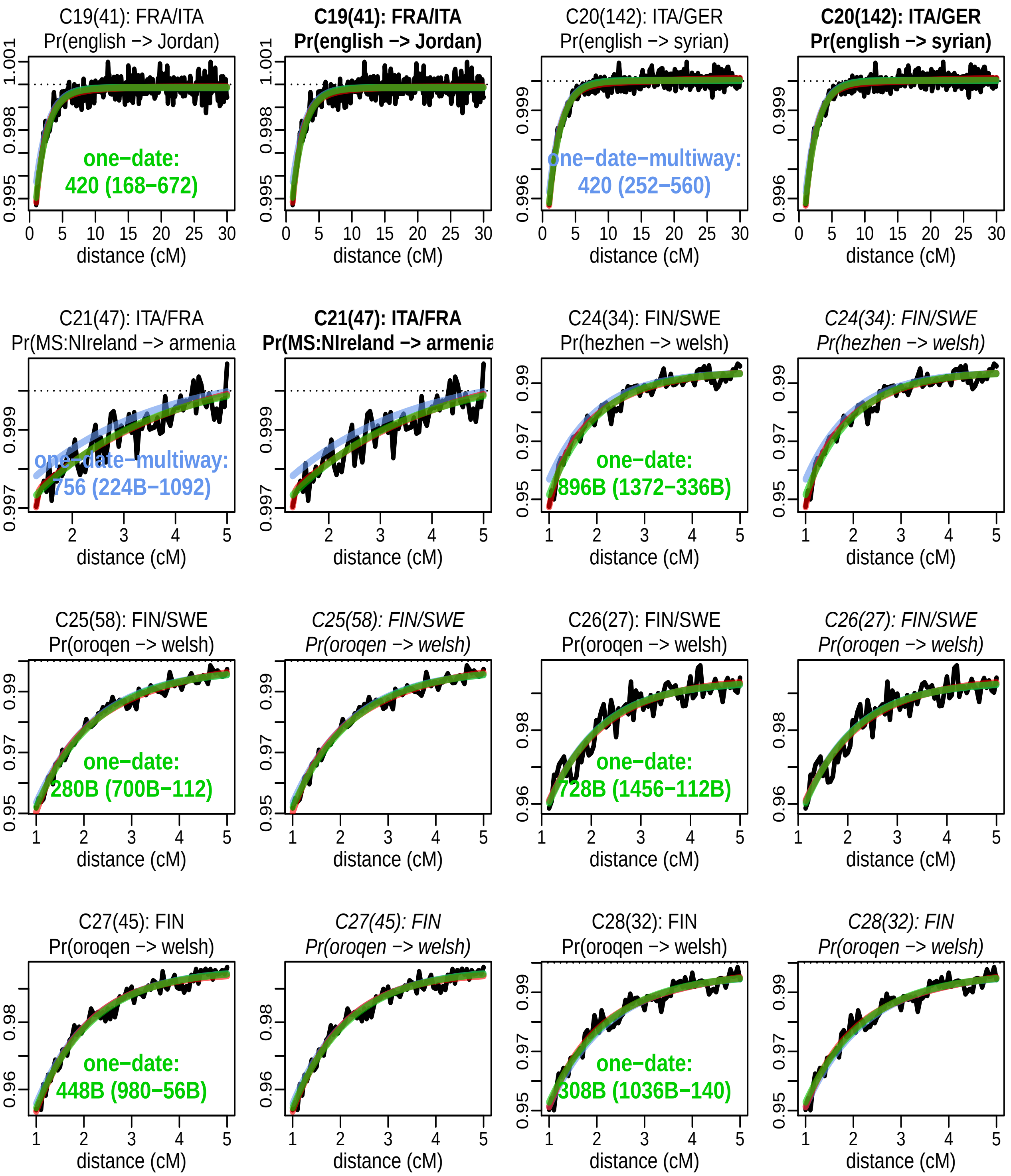

Figure S9: FastGLOBETROTTER's admixture probability curves for Europe clusters C19-C21 and C24-C28. See Figure S7 legend for details.

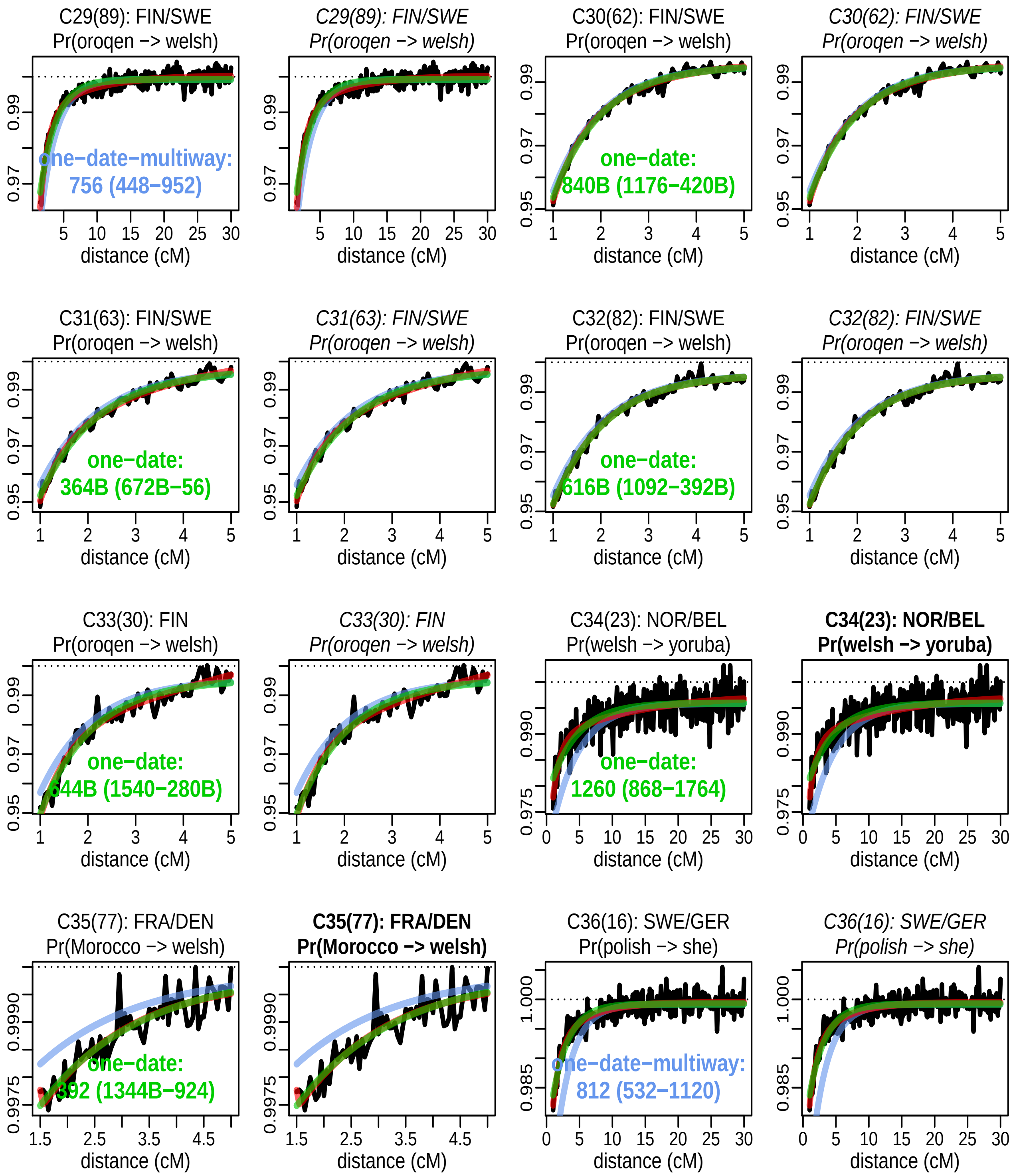

Figure S10: FastGLOBETROTTER's admixture probability curves for Europe clusters C29-C36. See Figure S7 legend for details.

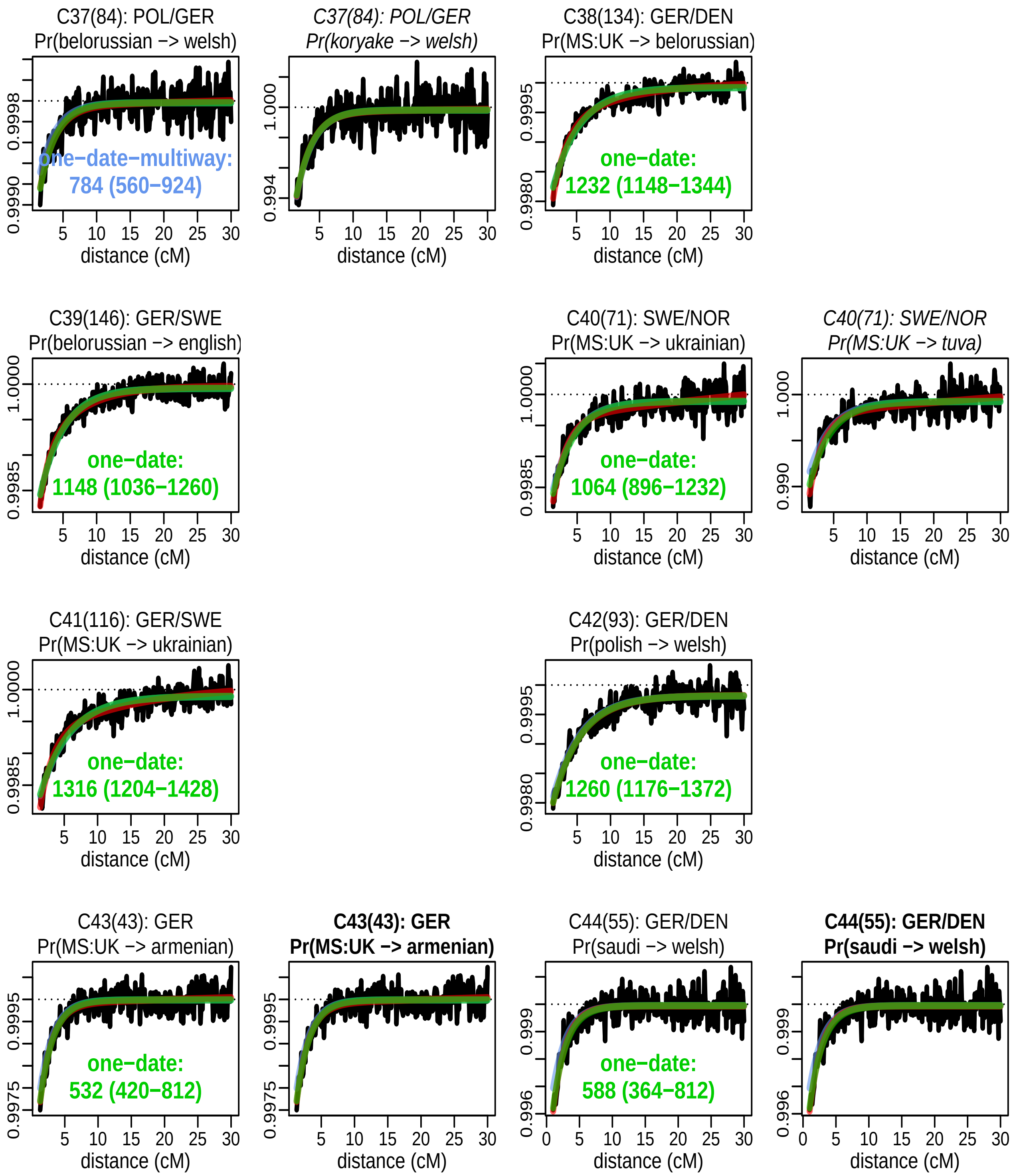

Figure S11: FastGLOBETROTTER's admixture probability curves for Europe clusters C37-C44. See Figure S7 legend for details.

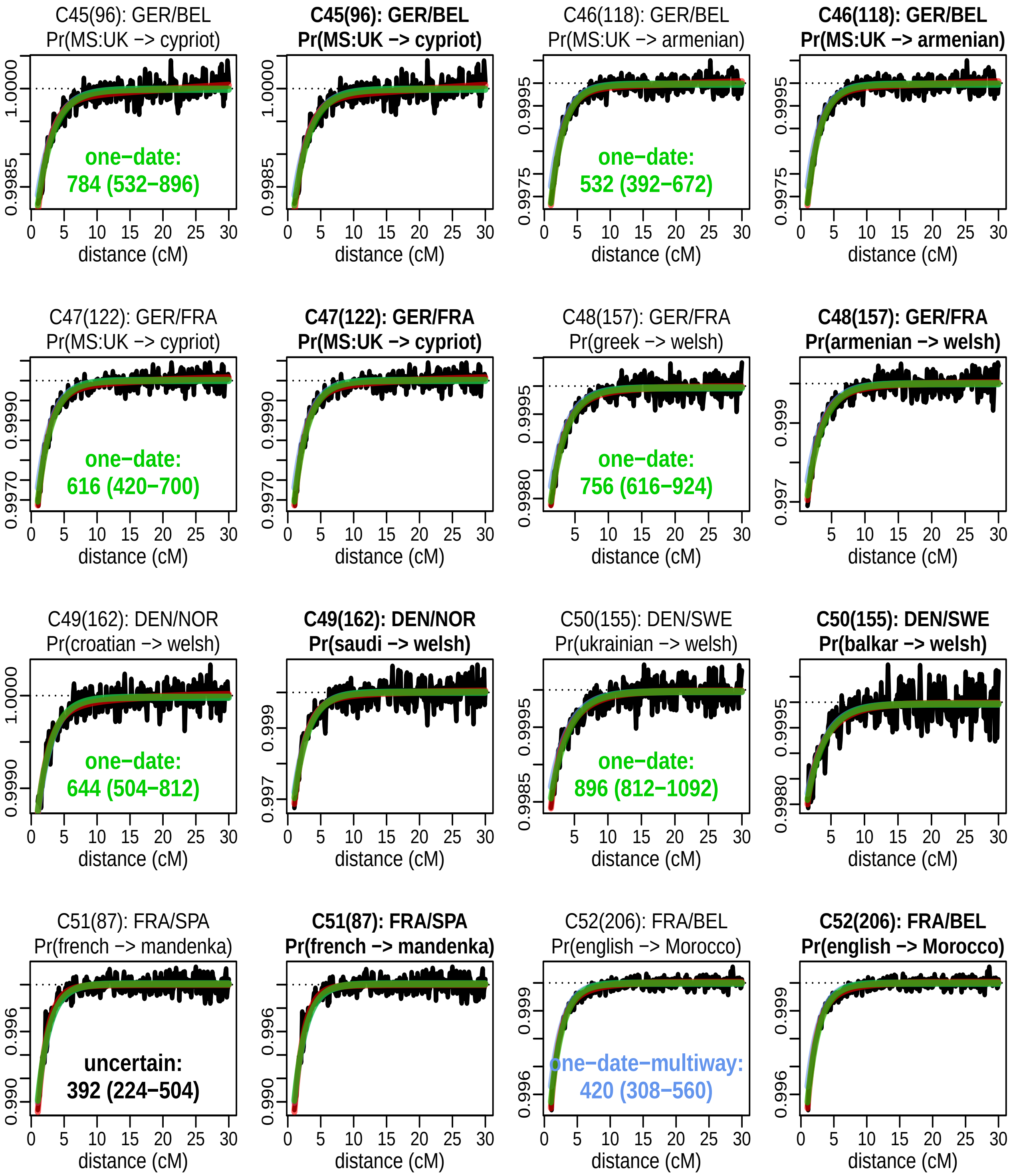

Figure S12: FastGLOBETROTTER's admixture probability curves for Europe clusters C45-C52. See Figure S7 legend for details.

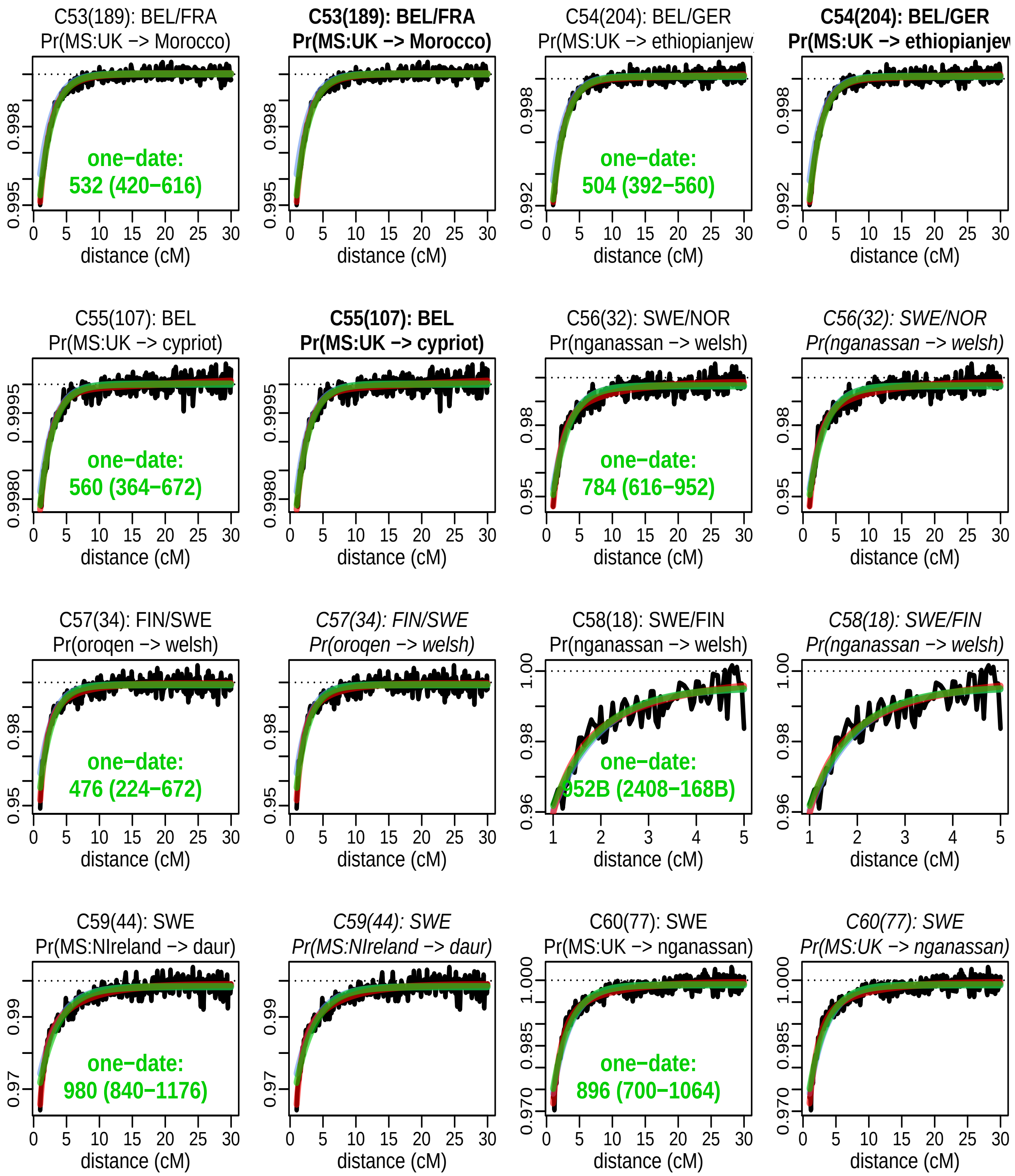

Figure S13: FastGLOBETROTTER's admixture probability curves for Europe clusters C53-C60. See Figure S7 legend for details.

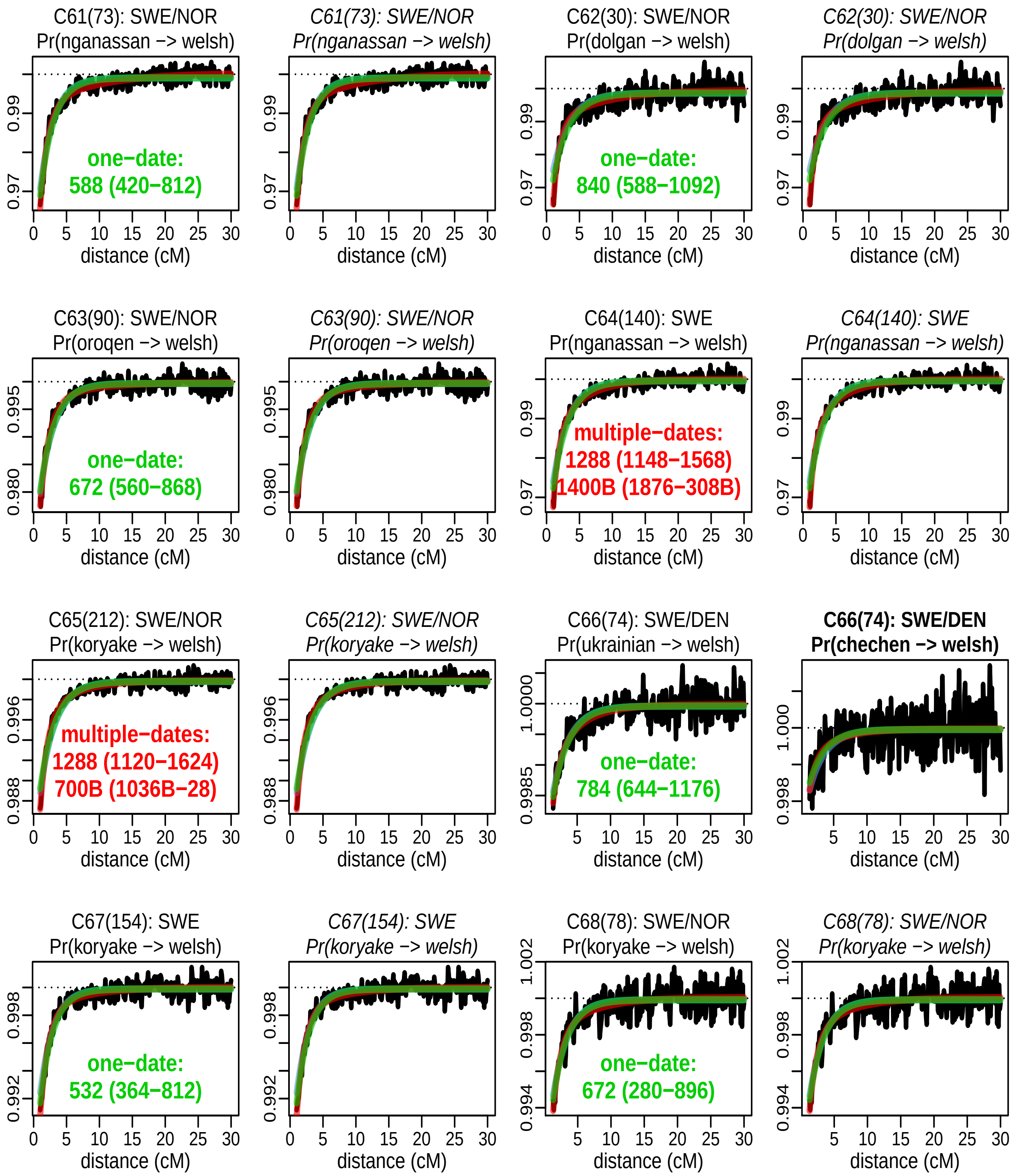

Figure S14: FastGLOBETROTTER's admixture probability curves for Europe clusters C61-C68. See Figure S7 legend for details.

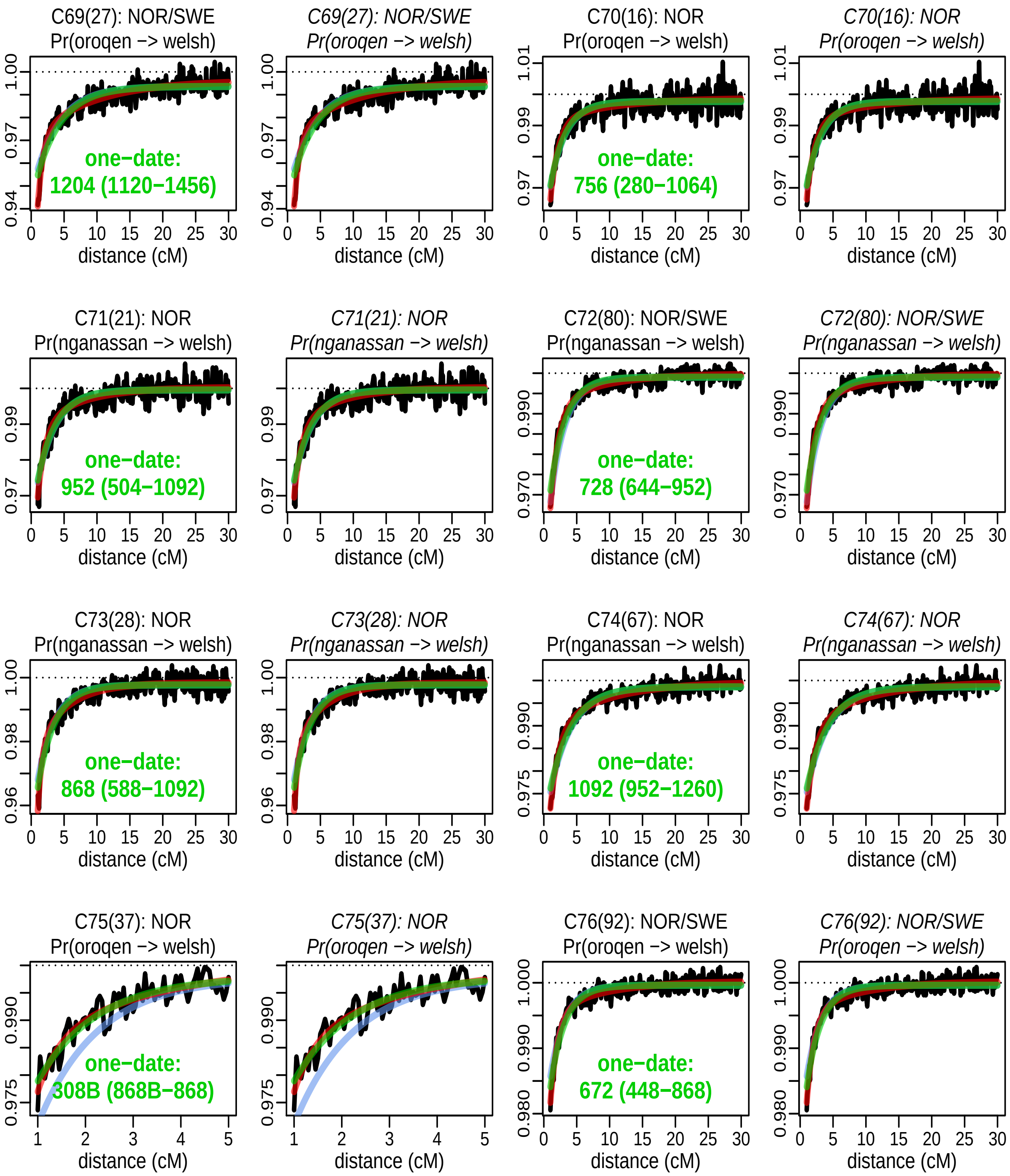

Figure S15: FastGLOBETROTTER's admixture probability curves for Europe clusters C69-C76. See Figure S7 legend for details.

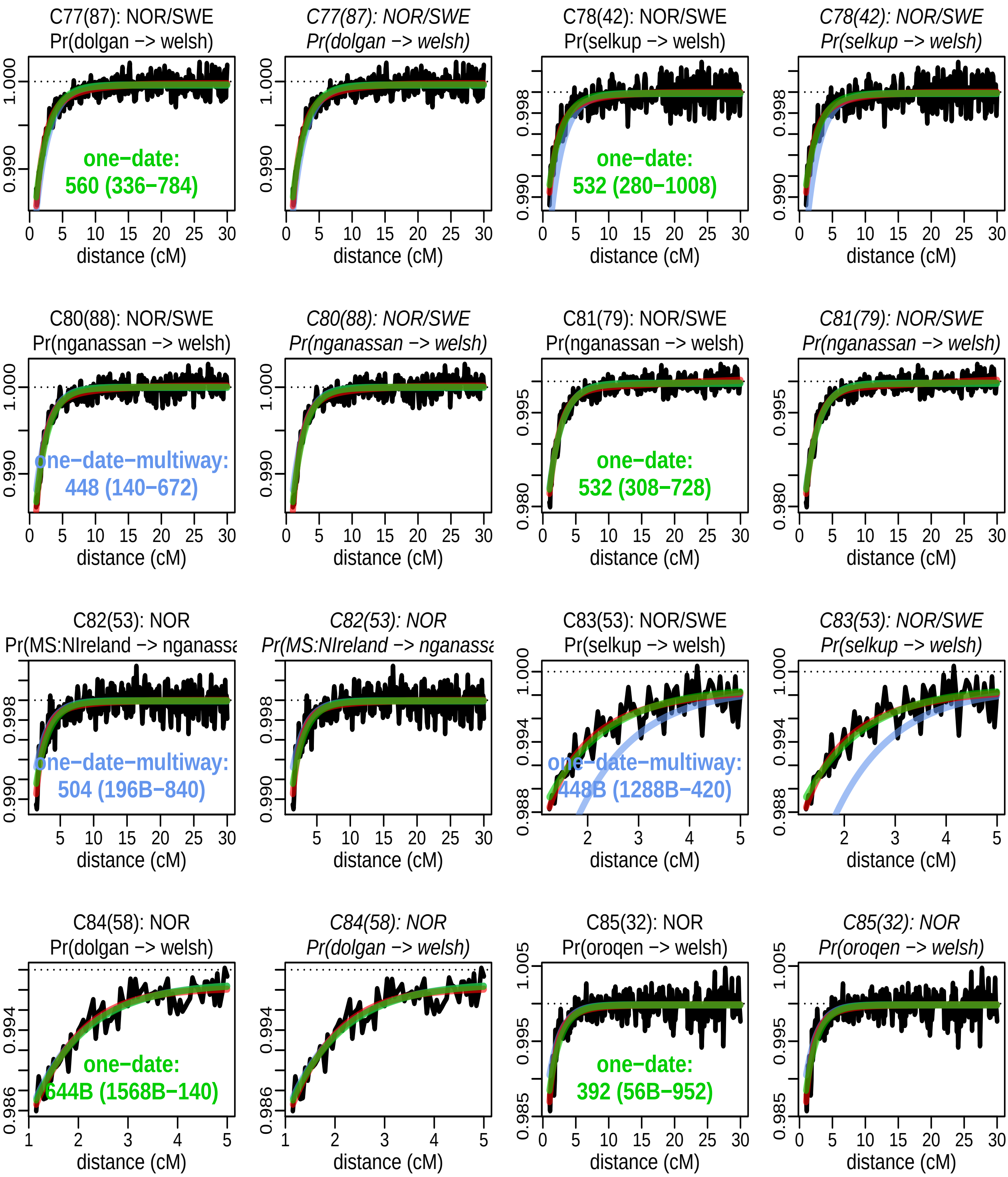

Figure S16: FastGLOBETROTTER's admixture probability curves for Europe clusters C77-C78 and C80-C85. See Figure S7 legend for details.

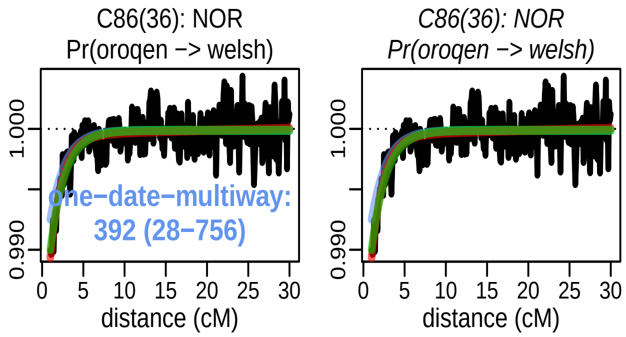

Figure S17: FastGLOBETROTTER's admixture probability curves for Europe cluster C86. See Figure S7 legend for details.

References

[1] G. Hellenthal, G.B.J. Busby, G. Band, J.F. Wilson, C. Capelli, D. Falush, and S. Myers. A genetic atlas of human admixture history. *Science*, 343:747–751, 2014.

[2] R.R. Hudson, M. Slatkin, and W.P. Maddison. Estimation of Levels of Gene Flow from DNA Sequence Data. *Genetics*, 132:583–589, 1992.

[3] D.M. Behar, B. Yunusbayev, M. Metspalu, E. Metspalu, S. Rosset, J. Parik, S. Rootsi, G. Chaubey, I. Kutuev, G. Yudkovsky, E.K. Khusnutdinova, O. Balanovsky, O. Semino, L. Pereira, D. Comas, D. Gurwitz, B. Bonne-Tamir, T. Parfitt, M.F. Hammer, K. Skorecki, and R. Villems. The genome-wide structure of the Jewish people. *Nature*, 466:238–242, 2010.

[4] G. Chaubey, M. Metspalu, Y. Choi, R. Mägi, I. G. Romero, P. Soares, M. van Oven, D. M. Behar, S. Rootsi, G. Hudjashov, C. B. Mallick, M. Karmin, M. Nelis, J. Parik, A. G. Reddy, E. Metspalu, G. van Driem, Y. Xue, C. Tyler-Smith, K. Thangaraj, L. Singh, M. Remm, M.B. Richards, MM. Lahr, M. Kayser, R. Villems, and T. Kivisild. Population genetic structure in Indian Austroasiatic speakers: the role of landscape barriers and sex-specific admixture. *Mol Biol and Evol*, 28(2):1013–1024, 2011.

[5] International HapMap Consortium. Integrating common and rare genetic variation in diverse human populations. *Nature*, 467:52–58, 2010.

[6] B.M. Henn, Gignoux C.R., M. Jobin, J.M. Granka, J.M. Macpherson, J.M. Kidd, L. Rodríguez-Botigué, S. Ramachandran, .L. Hon, A. Brisbin, A.A. Lin, P.A. Underhill, D. Comas, K.K. Kidd, P.J. Norman, P. Parham, C.D. Bustamante, J.L. Mountain, and M.W. Feldman. Hunter-gatherer genomic diversity suggests a southern African origin for modern humans. *Proc Natl Acad Sci USA*, 108(13):5154–5162, 2011.

[7] U. Hodoğlugil and R.W. Mahley. Turkish population structure and genetic ancestry reveal relatedness among Eurasian populations. *Annals Hum Genet*, 76(2):128–141, 2012.

[8] J.Z. Li, D.M. Absher, H. Tang, A.M. Southwick, A.M. Casto, S. Ramachandran, H.M. Cann, G.S. Barsh, M. Feldman, L.L. Cavalli-Sforza, and R.M. Myers. Worldwide human relationships inferred from genome-wide patterns of variation. *Science*, 319:1100–1104, 2008.

[9] Metspalu, M. and Romero, I.G. and Yunusbayev, B. and Chaubey, G. and Mallick, C.B. and Hudjashov, G. and Nelis, M. and Magi, R. and Metspalu, E. and Remm, M. and Pitchappan, R. and Singh, L. and Thangaraj, K and Villems, R. and Kivisild, T. Shared and unique components of human population structure and genome-wide signals of positive selection in South Asia. *Am J Hum Genet*, 89:731–744, 2011.

[10] M. Rasmussen, Y. Li, S. Lindgreen, J.S. Pedersen, A. Albrechtsen, I. Moltke, M. Metspalu, E. Metspalu, T. Kivisild, R. Gupta, M. Bertalan, K. Nielsen, M.T.P. Gilbert, Y. Wang, M. Raghavan, P.F. Campos, H.M. Kamp, A.S. Wilson, A. Gledhill, S. Tridico, M. Bunce, E.D. Lorenzen, J. Binladen, X. Guo, J. Zhao, X. Zhang, H. Zhang, Z. Li, M. Chen, L. Orlando, K. Kristiansen, M. Bak, N. Tommerup, C. Bendixen, T.L. Pierre, B. Grønnow, M. Meldgaard, C. Andreasen, S.A. Fedorova, L.P. Osipova, T.F.G. Higham, C.B. Ramsey, T.v.O. Hansen, F.C. Nielsen, M.H. Crawford, S. Brunak, T. Sicheritz-Pontén, R. Villems, R. Nielsen, A. Krogh, J. Wang, and E. Willerslev. Ancient human genome sequence of an extinct Palaeo-Eskimo. *Nature*, 463(7282):757–762, 2010.

[11] S. Sawcer, G. Hellenthal, M. Pirinen, C. Spencer, The International Multiple Sclerosis Genetics Consortium, and The Wellcome Trust Case Control Consortium. Genetic risk and a primary role for cell-mediated immune mechanisms in multiple sclerosis. *Nature*, 476:214–219, 2011.

[12] B. Yunusbayev, M. Metspalu, M. Järve, I. Kutuev, S. Rootsi, E. Metspalu, D.M. Behar, K. Varendi, H. Sahakyan, R. Khusainova, L. Yepiskoposyan, E.K. Khusnutdinova, P.A. Underhill, T. Kivisild, and R. Villems. The Caucasus as an Asymmetric Semipermeable Barrier to Ancient Human Migrations. *Mol Biol and Evol*, 29(1):359–365, 2012.

[13] G.K. Chen, P. Marjoram, and J.D. Wall. Fast and flexible simulation of DNA sequence data. *Genome Res*, 19:136–142, 2009.

[14] B. Efron and G. Gong. A Leisurely Look at the Bootstrap, the Jackknife, and Cross-Validation. *The American Statistician*, 37(1):36–48, 1983.
